# Characterization of in vitro transcriptional responses of dorsal root ganglia cultured in the presence and absence of blastema cells from regenerating salamander limbs

**DOI:** 10.1002/reg2.14

**Published:** 2014-07-14

**Authors:** Antony Athippozhy, Jeffrey Lehrberg, James R. Monaghan, David M. Gardiner, S. Randal Voss

**Affiliations:** ^1^ Department of Biology and Spinal Cord and Brain Injury Research CenterUniversity of KentuckyLexingtonKY40506USA; ^2^ Department of Developmental and Cell BiologyUniversity of California IrvineIrvineCA92697USA; ^3^ Department of BiologyNortheastern UniversityBostonMA02115USA

**Keywords:** Axolotl, blastema, dorsal root ganglia, limb regeneration, Mexican axolotl, nerve

## Abstract

During salamander limb regeneration, nerves provide signals that induce the formation of a mass of proliferative cells called the blastema. To better understand these signals, we developed a blastema−dorsal root ganglia (DRG) co‐culture model system to test the hypothesis that nerves differentially express genes in response to cues provided by the blastema. DRG with proximal and distal nerve trunks were isolated from axolotls (*Ambystoma mexicanum*), cultured for 5 days, and subjected to microarray analysis. Relative to freshly isolated DRG, 1541 Affymetrix probe sets were identified as differentially expressed and many of the predicted genes are known to function in injury and neurodevelopmental responses observed for mammalian DRG. We then cultured 5‐day DRG explants for an additional 5 days with or without co‐cultured blastema cells. On day 10, we identified 27 genes whose expression in cultured DRG was significantly affected by the presence or absence of blastema cells. Overall, our study established a DRG−blastema in vitro culture system and identified candidate genes for future investigations of axon regrowth, nerve−blastema signaling, and neural regulation of limb regeneration.

## Introduction

Salamanders and humans have structurally homologous tetrapod limbs, but only in salamanders is this structure capable of regeneration. When salamanders lose parts of their limbs from injuries, cells adjacent to the injury site are recruited to form a mass of proliferative mesenchymal cells called the blastema. The blastema grows and eventually becomes a self‐organizing structure that provides progenitor cells for regrowth and patterning of the missing limb (McCusker and Gardiner 2013). One of the earliest discoveries of an essential regulator of the process of blastema formation and subsequent limb regeneration was that severing the nerve supply either “retarded or entirely prevented” limb regeneration (Todd 1823). More than a century and a half later, researchers are still looking for the elusive factor(s) that the nerve provides in order to exert its effect on regeneration. Over the years many putative factors have been investigated, and now with the availability of new techniques and genomic resources it is possible to identify specific gene regulatory networks associated with this phenomenon.

Shortly after limb amputation, nerve fibers regenerate and grow distally into the injured limb tissues where they interact with the newly healed wound epithelium (Singer 1949; Thornton and Thornton 1970; Satoh et al. 2008). Singer (1978) proposed that these regenerating nerves supply trophic factors (referred to in the regeneration literature as “neurotrophic factor(s)”) independent of electrical transmission that support blastema cell proliferation during the early and mid phases of regeneration. Several molecules have been proposed as the putative nerve‐derived trophic factors, including fibroblast growth factors (FGF) (Mullen et al. 1996; Satoh et al. 2008), substance P (Globus et al. 1991), neuregulin (Wang et al. 2000), and transferrin (Mescher et al. 1997). Nerve‐derived signals in theory could stimulate blastema cell proliferation either directly or indirectly by signaling to non‐neuronal cells of the nerve sheath to release trophic factors. For example, anterior gradient protein 2 (*agr2)* is expressed in Schwann cells but not in neurons, and can rescue regeneration of partially innervated newt limbs (Kumar et al. 2007). Similarly, the early wound epithelium (WE) and later apical epithelial cap (AEC) appear to be direct targets of nerve signaling (Satoh et al. 2008, 2012) and they could signal secondarily to the underlying mesenchymal cells; for example, FGF8 produced in the WE/AEC would stimulate blastema cell proliferation as it does in developing limb buds (Han et al. 2001).

Several properties of the neurotrophic factor(s) have been identified over many decades of research. In the case of sensory neurons, the factor is thought to be produced in the cell bodies located in the dorsal root ganglia (DRG), transported distally along nerve fibers and released distally at sites where the sensory fibers interact with basal keratinocytes of the WE (Wallace 1972; Scadding 1988; Kiffmeyer et al. 1991; Satoh et al. 2008, 2012). The factor(s) is produced by sympathetic, motor, and sensory nerves innervating the limb (Singer 1952, 1974), as well as by the spinal cord (Boilly and Albert 1988), brain (Singer et al. 1976), and eye (Pietsch and Webber 1965). Finally, a critical threshold of nerve fibers is necessary for the limb to regenerate, such that if the number of nerve fibers innervating the limb is greater than this threshold, the limb will regenerate. Conversely, if the number of fibers is experimentally reduced below this threshold, the limb fails to regenerate (Singer 1952).

The properties above suggest that the neural signaling factor is transcribed by sensory neurons whose cell bodies are located in DRG (e.g., Satoh et al. 2008). How these cells respond to injury resulting from amputation as well as their subsequent interactions with cells of the regenerating limb blastema is complex. Initially, a nerve responds to and recovers from axotomy, a process that occurs during the first few days following amputation (Singer 1952). During this phase of regeneration, it is likely that genes encoding proteins necessary for cell survival, apoptosis, and neural development are transcribed. As axons regrow and re‐innervate tissues, it is also likely that the regenerating nerves respond to, as well as signal to, the target tissues in the blastema. Consistent with this model of bi‐directional signaling between nerves and blastema cells (both mesenchymal cells and keratinocytes of the WE/AEC) is the observation that, when DRG or spinal cord explants are co‐cultured with blastemas, there is an enhanced outgrowth of axons that orient towards the blastema (Bauduin et al. 2000; Tonge and LeClere 2000; Dmetrichuk et al. 2005). This response to the presence of a blastema in vitro suggests that a similar signaling mechanism operates in vivo to modulate transcription and translation of proteins that enhance and direct outgrowth of the regenerating nerve fibers. Unraveling the complexity of reciprocal nerve−blastema signaling is key to identifying the neurotrophic requirement for blastema formation and growth. To achieve this goal, we are developing experimental in vitro models that will allow us to identify the temporal sequence of regeneration‐specific transcriptional responses from both nerves and blastema cells.

In this study we focused on the response of regenerating DRG neurons and associated cells to signaling from the blastema. To do this, we modified the in vitro DRG−blastema co‐culture model of Tonge and LeClere (2000) to test the hypothesis that signaling from blastema cells regulates gene transcription by cells within the regenerating nerve. We isolated DRG along with their proximal and distal nerve trunks from axolotls (*Ambystoma mexicanum*), and cultured them in the presence or absence of explanted medium bud blastemas. Since the nerve is composed of many different cell types including Schwann cells, fibroblasts, and endothelial cells in addition to the neurons, we have identified changes in gene expression in the nerve as a whole. This reflects the situation in vivo in which all these cell types could potentially interact with and respond to signals originating from the blastema. We discovered that more than 1500 genes change expression in DRG and the nerve trunk during the first 5 days of culture, during which time the explanted DRG heal and begin to regenerate. After an additional 5 days of culture, a much smaller group of 27 genes were expressed differentially by DRG in response to the presence of co‐cultured blastema cells.

## Results

### Culture of dorsal root ganglia and blastema explants

**Figure 1:**
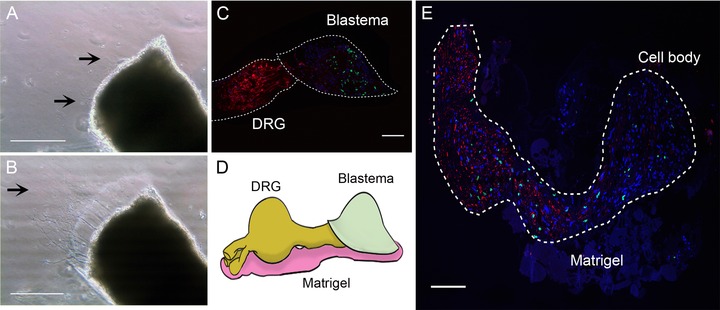
Axolotl dorsal root ganglia (DRG) in vitro. (A) A bright field image of a DRG in vitro 24 h after being explanted. Regenerating neural projections (arrows) are observed at the transected end of the nerve trunk. (B) Image of the same DRG after 4 days of culture in vitro. Neurite outgrowth (arrow) from the cut end of the nerve trunk was robust. (C) Fluorescent image of a sagittal section of a DRG−blastema co‐culture. The blastema was placed on the distal cut end of the DRG with the proximal region of the blastema coming into contact with the regenerating neurites. Nerve fibers were visualized by immunostaining of acetylated *α*‐tubulin (red), and cell nuclei were stained with 4',6‐diamidino‐2‐phenylindole (DAPI) (blue). Proliferating cells were detected with 5‐ethynyl‐2′‐deoxyuridine (EdU) (green). (D) Diagram of DRG−blastema co‐culture set‐up. The DRG and blastema were adhered to the bottom of the cell culture insert with a drop of growth factor reduced matrigel. (E) Immunofluorescence image of a longitudinal section of a DRG in vitro. The DRG was adhered to the cell culture with matrigel. Phosphorylated neurofilaments were visualized by immunostaining with RT97 (red). Cell nuclei were stained with DAPI (blue). Proliferating cells were detected with EdU (green). Scale bars 0.5 mm.

Most DRG explants survived after surgical excision and began to regenerate axons after about 24 h in culture (Fig. 1A). Initially, a few growing neurites were observed to be extending from the distal cut end of the nerve, and over the next several days of culture, increasing numbers of neurites were observed (Fig. 1B). Explants that did not exhibit initial neurite outgrowth at 24 h or exhibited continued outgrowth at 5 days were discarded and not used in the experiments.

In addition to regenerating neurites, the morphology of DRG (that were used experiments) appeared normal in histological sections (Fig. 1C and E). 4',6‐Diamidino‐2‐phenylindole (DAPI) stained nuclei within both the DRG and the distal nerve trunk appeared normal with little evidence of pyknosis (Fig. 1E). Cells within both the DRG and the nerve trunk were proliferating as evidenced by the presence of 5‐ethynyl‐2′‐deoxyuridine (EdU) positive nuclei (green). Proliferation of cells within both the DRG and the nerve trunk in response to peripheral nerve injury in vivo has been reported previously (Clemence et al. 1989; Zochodne 2012). Neurites were present in the nerve trunk in DRG explants that were cultured either alone (Fig. 1E) or in association with blastema explants (Fig. 1C) as evidenced by the presence of phosphorylated neurofilaments (stained with the NRT97 antibody, red). The appearance of the NRT97‐positive neurofilaments within cultured axolotl DRG was similar to what has been reported previously for injured peripheral nerves in vivo (Lawson et al. 1984; Bergman et al. 1999). Thus, axolotl DRG appear to be viable and morphologically comparable to DRG in vivo, as has been reported previously (Tonge and LeClere 2000),

After 5 days of culture in vitro, DRG that appeared healthy with extensive neurite outgrowth were selected for co‐culture with explanted medium bud stage blastemas (Fig. 1D). As with the explanted and cultured DRG, the blastema cells appeared healthy. Most nuclei appeared normal when observed in DAPI stained tissue sections, and blastema cells continued to proliferate as evidenced by the incorporation of EdU (Fig. 1C). Studies to further characterize the response of explanted blastema cells to culture conditions and to signals from DRG are in progress.

### Differentially expressed genes identified from in vitro cultured DRG

**Figure 2:**
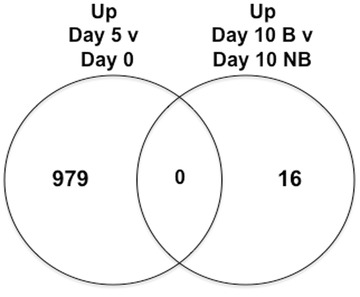
Venn diagram showing the number of upregulated, differentially expressed genes identified between time and treatment contrasts. Substantially more genes were identified as differentially expressed between day 0 and day 5 cultured dorsal root ganglia (DRG) than between day 10 DRG with blastema (10 B) and day 10 DRG without blastema (10 NB).

**Figure 3:**
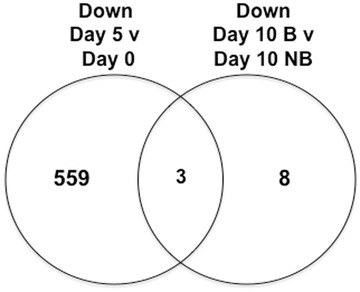
Venn diagram showing the number of downregulated, differentially expressed genes identified between time and treatment contrasts. Substantially more genes were identified as differentially expressed between day 0 and day 5 cultured dorsal root ganglia (DRG) than between day 10 DRG with blastema (10 B) and day 10 DRG without blastema (10 NB).

A total of 1541 probe sets were identified as differently expressed between DRG at the time of removal from the donor animal (day 0) and DRG that had been cultured in vitro for 5 days (Figs. 2, 3 and Table S1). Of this total, 1498 probe sets showed significant sequence identity to human RefSeq proteins (*E* ≤ 1 × 10^−7^) and we considered these to be salamander−human orthologous genes in the enrichment analyses described below. We note that >60 of these genes were identified by two to four independent probe sets. Thus, overall, statistical significance was validated for approximately 5% of the differentially expressed genes.

The majority of these probe sets (*N* = 979) presented higher transcript abundances at day 5 than day 0, a pattern indicating that expression of the corresponding genes was upregulated in response to injury (Fig. 2 and Table S1). For many of the genes associated with these probe sets, the magnitude of the change was dramatic, with 135 showing more than a 5‐fold increase in expression (Table S1). Of these upregulated genes, many are known to function in the regulation of inflammatory and innate immune responses (e.g., *lep*, *il8*, *il1b*, *tgfb1*, *irf1*, *thbs1*, *mdm2*, *hmox1*). Similarly, genes associated with cellular growth and developmental regulation (e.g., *bdnf*, *ctgfntf3*, *gadd45b*, *gadd45g*) and axon development and regrowth (e.g., *bmp2*, *smad1*, *creb1*, *bcl2l1*, *ankrd1*, *npy*, *robo1*) were upregulated. A number of over‐represented biological process and Panther protein class ontology terms were identified based on genes that presented higher transcript abundances at day 5 than day 0 (Table 1). These included immune system processes, RNA and nucleic acid metabolic processes, as well as the MAPKKK signaling cascade that were identified as significantly enriched biological processes. Also, several RNA protein class gene ontologies were identified as significant, including mRNA processing factor and RNA/ribonucleoprotein binding protein (Table 1).

**Table 1:** List of statistically significant (*P* < 0.05) over‐represented biological process (BP) and Panther protein class (PC) ontology terms for genes identified as differentially expressed in axolotl dorsal root ganglia between day 0 and day 5 of culture in vitro

Upregulated at day 5	Observed	Expected
Nucleobase, nucleoside, nucleotide and nucleic acid metabolic process: BP	193	118
RNA metabolic process: BP	61	22
mRNA processing	42	14
Nuclear mRNA splicing, via spliceosome: BP	34	10
Metabolic process: BP	343	283
Primary metabolic process: BP	326	268
MAPKKK cascade: BP	24	9
Response to stimulus: BP	52	30
Immune system process: BP	74	48
RNA binding protein: PC	85	40
Nucleic acid binding: PC	151	92
Ribonucleoprotein: PC	16	4
mRNA processing: PC	21	8
Downregulated at day 5		
Muscle contraction: BP	16	5
Cytoskeletal protein: PC	36	15
Extracellular matrix structural protein: PC	8	1
Actin family cytoskeletal protein: PC	20	8

In terms of genes that were downregulated (*N* = 562), only muscle contraction was identified as a statistically enriched biological process (Table 1). Downregulated genes associated with cytoskeletal and extracellular matrix protein classes were also identified as statistically enriched. As was observed for upregulated genes, some of the downregulated genes are well‐established regulators of cellular growth and development (*gas6* and *kit*). In addition, Schwann cell biomarkers (*pmp22*, *mbp*, *plp1*, *gfap*) showed lower transcript abundances at day 5 relative to day 0. The results indicate that, after DRG are excised and cultured for 5 days, transcript abundances change for >1500 genes. The predicted functions for these differentially expressed genes are consistent with the conclusion that cultured DRG mount robust injury and neurodevelopmental responses, including changes in cytoskeletal structure, the extracellular matrix, and RNA processes associated with transcriptional and post‐transcriptional regulation.

### Effect of blastema cell co‐culture on DRG transcription

We compared transcript abundance estimates between the two day 10 treatments (DRG co‐cultured with blastema cells or DRG cultured alone from day 5 to day 10). A total of 27 genes were identified as differentially expressed in DRG in response to the presence of a co‐cultured blastema (Table 2). Of these differentially expressed genes, 16 probe sets registered significantly higher transcript abundances in DRG−blastema co‐cultures. Eleven of these have predicted gene names: *areg*, *krt15*, *krt17*, *mall*, *cryba2*, *c3orf54*, *col22A1*, *marcks*, *chd3*, *kazald1*, and *tacc3*. A total of 11 probe sets registered lower transcript abundances in DRG that were co‐cultured with a blastema—*abat*, *smc2*, *znf697*, *gas6*, *napa*, *idh3g*, *dnm1l*, *ndc80*, *asap1*, *esco2*—and one unannotated probe (probe set ID *axo25121*). Of these 27 probe sets, the greatest expression difference was observed for *krt17* and *axo31729‐*f, which were expressed 7.8‐ and 4.2‐fold higher respectively in response to the presence of a co‐cultured blastema. These results show that the presence of a blastema affected transcription of relatively few but potentially important genes that are expressed by cultured DRG. We did not observe differences in the level of expression of the blastema marker genes *prrx1*, *msx2*, and *hoxa13* between DRG samples with or without co‐cultured blastemas, indicating that blastema cells did not contaminate the DRG samples.

**Table 2:** Probe sets registering significant fold changes in transcript abundances for dorsal root ganglia (DRG)−blastema co‐cultures. Fold change is measured as the ratio of day 10 DRG cultured with a blastema compared with day 10 DRG cultured without a blastema. Probe sets that are unannotated are displayed as the probe set ID with the prefix “axo”

Upregulated at day 10	Fold change	*P*‐value
Keratin 17	7.82	2.44E‐05
axo31729‐f	4.21	1.25E‐04
Kazal‐type serine peptidase inhibitor domain 1 precursor	3.14	4.47E‐03
axo31698‐f	2.68	3.90E‐05
Mal, T‐cell differentiation protein‐like	2.52	1.02E‐03
Amphiregulin preproprotein	2.19	1.15E‐03
Keratin 15	2.11	2.64E‐03
axo31329‐f	1.74	8.87E‐05
Myristoylated alanine‐rich protein kinase C substrate	1.44	9.77E‐03
Crystallin, beta A2	1.42	1.76E‐03
axo28274‐f	1.39	1.85E‐03
Similar to collagen, type XXII, alpha 1	1.39	8.02E‐03
CPEB‐associated factor Maskin	1.37	4.38E‐03
Chromodomain helicase DNA binding protein 3 isoform 1	1.36	1.01E‐02
axo30442‐f	1.28	4.22E‐03
Hypothetical protein LOC389119	1.28	1.18E‐02
Downregulated at day 10		
*N*‐ethylmaleimide‐sensitive factor attachment protein, alpha	1.79	5.72E‐03
Establishment of cohesion 1 homolog 2	1.69	1.24E‐02
Kinetochore associated 2	1.64	7.51E‐03
Structural maintenance of chromosomes 2	1.63	1.81E‐04
Development and differentiation enhancing factor 1	1.57	3.26E‐03
4‐Aminobutyrate aminotransferase precursor	1.57	2.15E‐03
Isocitrate dehydrogenase 3 (NAD+) gamma isoform a precursor	1.53	6.86E‐03
Zinc finger protein 697	1.52	1.11E‐02
axo25121‐f	1.48	3.30E‐03
Dynamin 1‐like isoform 3	1.42	2.21E‐03
Growth arrest‐specific 6 isoform 1	1.31	1.17E‐02

## Discussion

An unresolved question in regeneration biology concerns the nature of the functional relationship between nerves and blastema cells. It is well documented that nerves are required for limb regeneration in salamanders, and consequently most regeneration studies have focused more on the signals that nerves may be providing (referred to in the regeneration literature as neurotrophic factor(s)) rather than the signals that the blastema provides to induce and guide the regeneration of the nerve (Globus et al. 1991; Mullen et al. 1996; Mescher et al. 1997; Wang et al. 2000; Satoh et al. 2008). In order to identify the genes involved in this reciprocal interaction, we have been working to develop a nerve (DRG) and blastema co‐culture model. In this study we have used this model to test the hypothesis that blastema cells provide signals that regenerating nerves respond to. By culturing DRG we analyzed the transcriptional response not only of the neurons, but also the Schwann cells, fibroblasts, and other cells that are found in the nerve trunk that may be functionally important for the interaction between the nerve and the blastema in vivo. We observed that dissection and culture of DRG initiates dramatic changes in transcription after 5 days of in vitro culture that are consistent with the conclusion that nerves are recovering from injury and beginning to regenerate. At that point, the regenerating nerves respond to the presence of co‐cultured blastema cells and differentially express a relatively small number of genes that are targets for future functional studies. Below, we highlight several genes that code for proteins that are likely to be associated with cell growth and development, axon regrowth, and limb regeneration.

### Transcriptional analysis of limb regeneration processes using cultured salamander DRG

Salamanders are unique because they provide an opportunity to understand how tissues can be regenerated endogenously. If a salamander's limb or tail is amputated, this regeneration program is activated and the missing structures are regenerated. However, salamander regeneration is complex from a transcriptional perspective (e.g., Monaghan et al. 2007, 2009), involving thousands of gene expression changes. Accordingly, there is need to develop in vitro models that reduce the complexity of tissue regeneration and allow for an understanding of how the various tissues and cells respond to injury and pro‐regenerative signaling. The nerve (DRG) and blastema co‐culture model described here was utilized to test the hypothesis that there is reciprocal signaling between blastema cells and cells in the peripheral nerve that results in the differential transcription of genes required for limb regeneration.

Although there were specific changes in gene expression associated with interactions between DRG and blastemas, it is not known whether they occurred as a consequence of axonal retrograde transport of molecules from the blastema to DRG neuron cell bodies. It is possible that blastemas affected DRG transcription indirectly, for example via release of diffusible molecules such as retinoic acid (Scadding and Maden 1994; Prince and Carlone 2003.) and not via axon transport. Related to this question is the fact that the DRG consist of several different cell types (e.g., neurons, Schwann cells, fibroblasts, immune cells, and cells associated with the vasculature), and we do not have data indicating which specific cell types are involved in the observed transcriptional changes (e.g. in situ hybridization). The diversity of the gene expression responses that we observed is consistent with the conclusion that multiple cell types are involved in the response. For example, we observed a significant decrease in expression of Schwann‐cell‐associated transcripts (*pmp22*, *mbp*, *plp1*) after 5 days of in vitro DRG culture, suggesting either transcriptional repression of myelin‐associated proteins or progressive Schwann cell death. Similarly, cultured salamander DRG recapitulate transcriptional changes observed after mammalian sciatic nerve injury (Newton et al. 2000; Kubo et al. 2002; Xiao et al. 2002; Tanabe et al. 2003; Boeshore et al. 2004; Nilsson et al. 2005; Bosse et al. 2006; Stam et al. 2007), indicative of responses from immune cells (genes associated with inflammation) and from neurons (genes associated with nerve development and axonal regrowth).

### Candidate genes associated with axon regrowth and blastema cell proliferation

The DRG−blastema co‐culture model (in vitro) was designed to correspond to the time during limb regeneration when nerves have recovered from the injury of amputation and begun to function in the recruitment of proliferating blastemal cells (in vivo). To model this phase of regeneration, blastemas were placed in contact with neurites that had sprouted from the peripheral branch after 5 days of culture. Historically, nerve−blastema signaling has been modeled as a process where axons release molecules that directly stimulate blastema cell proliferation. However, nerve signaling may be indirect, with axons signaling wound epithelial cells, epithelial glands, or Schwann cells to secrete molecules to support blastema cell proliferation (Kumar et al. 2007, 2010; Satoh et al. 2012). We note that whether signaling occurs directly or indirectly, axon regrowth is a necessary first step in the genesis of the trophic effect required for blastema maturation, and axon regrowth after nerve injury requires transcription (Smith and Skene 1997).

In our study, DRG culture elicited a diverse transcriptional response involving many of the same gene expression changes observed in previous studies of cultured mammalian DRG as well as in DRG with axotomized peripheral nerves (Boeshore et al. 2004; Nilsson et al. 2005; Szpara et al. 2007). The vast majority of genes that were differentially expressed in cultured salamander DRG (compared with day 0) were expressed similarly in the presence or absence of a blastema. Thus, blastema co‐culture had a relatively minor effect on the overall DRG injury response program. Of the genes that were differentially expressed as a function of blastema co‐culture, several are predicted to regulate cellular growth and axon regrowth. The differentially expressed gene that exhibited the largest change in expression (up‐related in response to co‐cultured blastemas) was keratin 17 (*krt17*), which is rapidly induced in response to injury via the Akt/mTOR signaling pathway in mammals (Kim et al. 2006). Activation of the mTOR pathway after peripheral injury of murine DRG neurons is associated with axon regrowth and target innervation (Abe et al. 2010). Since *krt17* generally is associated with epithelial cells and appendages in mammals, and not DRG, further analysis of its regulation during axolotl regeneration is needed to determine its possible function in nerve−blastema interactions.

It is generally thought that the nerve requirement for limb regeneration is a consequence of one or a few trophic factors that are synthesized and secreted by nerves as they innervate the blastema niche (blastema cells and the overlying apical epithelium). Studies over the years have identified several characteristics of these presumptive trophic factors: they are secreted peptides or proteins; they are produced by sensory, motor, and autonomic nerves; they increase in level/activity in regenerating axons and after priming/conditional lesions; and they stimulate mitosis (Singer 1952; Kamrin and Singer 1959; Lebowitz and Singer 1970; Deck 1971; Globus and Liversage 1975; Singer et al. 1976; Globus and Vethamany‐Globus 1977; Choo et al. 1978; Carlone and Foret 1979; Boilly and Baudin 1988). We observed that expression of several trophic factors and neuropeptides increased significantly in cultured DRG whether or not they were co‐cultured with a blastema (*ntf3*, *batf3*, *npy*, *bdnf*, *npff*, *nts*, *ctgf*). Kumar et al. (2010) reported that ectopic expression of *agr2* rescues limb regeneration in partially innervated newt limbs, and expression is associated with Schwann and Leydig cells in the epidermis. We did not observe significant changes in *agr2* expression; however, a potential downstream target of *agr2*, *areg* (Dong et al. 2011), was upregulated modestly, but not significantly (∼1.5‐fold, *P* = 0.03), between day 0 and day 5 of culture, and then was expressed at a significantly higher level in DRG−blastema cultures compared with DRG cultured without a blastema. In mouse DRG, *areg* is one of the most highly upregulated genes in response to sciatic nerve transection and application of AREG to DRG cultures induces neurite outgrowth (Nilsson et al. 2005). As a member of the epidermal growth factory family, *areg* is a mitogen for Schwann cells and fibroblasts; the latter cell type gives rise to the majority of cells in the early axolotl blastema (Muneoka et al. 1986). Finally, *areg* is secreted by neurons and Schwann cells (Kimura et al. 1990) and thus could be produced locally at the wound site or in neural cell bodies and transported to blastema cells via regenerating nerves. Thus *areg* is a strong candidate for functioning as a neurotrophic factor given its pleiotropic potential to stimulate axon regrowth as well as blastema cell proliferation.

Our study identified *kazald1* as a second gene with properties that would be expected of a trophic factor that could stimulate proliferation of blastema cells. *Kazald1* expression was increased 3‐fold in day 10 DRG−blastema co‐cultures compared with day 10 DRG without blastemas. *Kazald1* is a secreted member of the insulin growth factor binding protein family that is transcriptionally upregulated during the early phase of bone formation and regeneration, and it is associated with proliferation of osteoblasts (Shibata et al. 2004).

Expression of a third signaling factor, *bmp2*, was upregulated 1.5‐fold in response to co‐culture with a blastema (with a *P*‐value of 0.015 which was greater than the cutoff of 0.0125 for genes listed in Table 2). *Bmp2* was significantly upregulated (nearly 3‐fold) during the initial period in culture (days 0−5) and its expression continued to increase when co‐cultured with a blastema. Members of the BMP family of growth factors are involved in the regulation of growth and pattern formation during both limb development and regeneration. Recently it has been demonstrated that ectopic limb blastemas can be induced in axolotls by treating wounds with a cocktail of BMP and FGF in vivo as a substitute for signaling from a nerve (Makanae et al. 2013). Further studies on the role of BMP2 signaling in the regulation of blastema cell proliferation are in progress. Previous studies demonstrated that expression of keratinocyte growth factor (*Fgf7*) is induced in DRG in response to limb amputation (Satoh et al. 2008). Since a probe set corresponding to the *Fgf7* gene is not present on the Amby_002 GeneChip, we could not document changes in *Fgf7* expression. We did not observe significant changes in the expression of other members of the FGF family, including *Fgf1*, *Fgf2*, *Fgf8* and *Fgf10*.

## Conclusion

Our results show that a robust transcriptional response is activated in cultured axolotl DRG that is comparable to the responses observed in mammalian DRG explanted into culture or after transection of peripheral nerves. Co‐culture with a blastema resulted in a low number of potentially important changes in gene expression in axolotl DRG. At least three of the differentially expressed genes that we identified encode secreted, mitogenic proteins, supporting classical ideas that nerves may provide one or a few factors that function in blastema formation and cell proliferation.

## Materials and methods

### Animal care and collection of DRG and blastema

The handling and surgical manipulation of axolotls was carried out according to University of California, Irvine (UCI), Animal Care and Use guidelines. Axolotls (*Ambystoma mexicanum*) measuring 15−20 cm from snout to tail tip were spawned at UCI or the Ambystoma Genetic Stock Center at the University of Kentucky. They were housed on a 12h light/dark cycle and fed ad libitum. DRG were collected post‐euthanasia by surgically removing the spinal nerves that innervated either the forelimb (spinal nerves 3, 4, and 5) or the hind limb (spinal nerves 15, 16, and 17). The nerves were severed where the dorsal and ventral nerve roots exit the spinal cord, and again 3 mm distal to the spinal ganglion.

### DRG and limb blastema co‐culture

DRG were cultured individually in 12‐well Nunc nunclon plates with 60% L‐15, 5% fetal bovine serum, 1% Insulin‐Transferrin‐Selenium, and gentamicin/amphotericin B (Sigma). Each DRG was attached to the bottom of the culture well by embedding it in a small drop (about 3 μL) of growth‐factor‐reduced BD matrigel (BD Biosciences). Only DRG explants that exhibited neurite outgrowth within the first 24 h of culture were used for subsequent experiments. DRG explants were cultured for 5 days and either collected for RNA extraction or assigned to two experimental treatments: (1) nerve−blastema co‐culture in which a medium bud blastema was placed directly on top of the regenerating region of the nerve, or (2) nerve culture without a co‐cultured blastema.

For the samples in which the DRG were co‐cultured with blastemas, we collected blastemas at the medium bud stage of regeneration from limbs that had been amputated several days earlier. The apical epithelium (AEC) was not removed prior to placing the blastemas adjacent to the cultured DRG such that the proximal cut end of the blastema covered newly sprouted neurites that were regenerating from the distal cut end of the DRG (Fig. 1C and D). The DRG and blastema were secured together with a small drop of growth‐factor‐reduced matrigel. For the DRG cultures without a blastema, procedures were the same as for the cultures with an added blastema, including removal of the medium, touching the surrounding matrigel with forceps, adding a drop of matrigel as described above, and then refilling the well with culture medium. Explants were cultured for an additional 5 days resulting in a total of 10 days in vitro after excision of the DRG from the donor animal. At 10 days of culture, the co‐cultured DRG were separated from the associated blastemas under a dissecting microscope to prevent blastema cell contamination and were collected for RNA extraction. A total of 15 DRG were pooled for each biological replicate and three replicates were analyzed at both the day 5 and day 10 time points. Three replicate control samples (day 0) were prepared by pooling non‐cultured DRG (12 per replicate) collected directly from euthanized salamanders.

### RNA isolation and microarray analysis

DRG were pooled within biological replicates prior to RNA isolation. RNA was isolated using Trizol (Invitrogen) in conjunction with the Nucleospin RNA XS Kit (Macherey‐Nagel) following the manufacturer's protocol. RNA quality was assessed using an ND‐1000 spectrophotometer (Nanodrop; Wilmington, DE) and a Bioanalyzer 2100 (Agilent; Santa Clara, CA). The 12 RNA samples that were obtained from the day 0, day 5, and day 10 treatments were analyzed using the Amby_002 GeneChip (Huggins et al. 2012). The University of Kentucky Microarray Core Facility generated biotin labeled cRNA targets for all samples and hybridized each to independent GeneChips. The GeneChips were scanned and processed using the RMA algorithm in Affymetrix's Expression Console software (Affymetrix, Santa Clara, CA).

### Statistical analysis of gene expression

Prior to statistical analyses, probe sets with low and variable expression values were filtered. Probe sets were filtered if they registered expression values below the maximum bottom quartile value (4.27 across all arrays) for one or more replicate arrays within a treatment. To identify differentially expressed genes, data were analyzed using one‐way ANOVA as implemented in JMP Genomics version 5.0 (SAS Institute, Cary, NC) and a significance cutoff of FDR = 0.05 (Benjamini and Hochberg 1995). Follow up *t*‐tests were used to detect significantly different changes in gene expression after the initial 5‐day period of DRG culture (day 0 vs. day 5) and between DRG cultured with and without blastemas at day 10, using a *P*‐value cutoff of 0.0125.

We used Panther (Thomas et al. 2003; Mi and Thomas 2009) to identify gene ontology terms that were statistically enriched in our lists of differentially expressed genes. All of the genes on the *Ambystoma* GeneChips with established orthologies to human protein coding sequences were used to generate expected values. We retained all over‐represented terms that were supported by >2 counts at a Bonferroni‐adjusted *α* of 0.05.

### Immunohistochemistry

Tissues were fixed for 3 h at room temperature in 4% paraformaldehyde and were then dehydrated in graded alcohol followed by xylene, embedded in paraplast, and sectioned at 5 μm. To perform immunohistochemistry sections were de‐paraffinized and rehydrated in Tris Buffered Saline with Tween (TBST). Sections were then incubated with anti‐acetylated α‐tubulin (Abcam, diluted 1:250) and anti‐RT97 (DSHB, diluted 1:250) overnight at 4°C. Sections were washed with TBST and incubated with anti‐mouse 594 (Abcam, diluted 1:250). Following secondary antibody treatment sections were washed with TBST and mounted with ProLong Gold Antifade with DAPI. To label proliferating cells, 80 μmol/L of 5‐ethynyl‐2′‐deoxyuridine (EdU) was added to culture media for 5 h. Following EdU labeling, tissues were processed as described above. Proliferating cells were then visualized using the Click‐It‐EdU Alexa‐Fluor 488 kit (Life Technologies).

## Supporting information

Additional Supporting Information may be found in the online version of this article at the publisher's website:

**Table S1**. List of 1541 probe sets that were identified as differentially expressed between dorsal root ganglia sampled on day 0 and day 5. Columns indicate if a gene was significantly upregulated or downregulated for a given statistical contrast. A “1” indicates that the probe set was differentially expressed and the corresponding change was detected, while a “0” indicates there was no significant change.
