# Supplementary material for: Characterization of in vitro transcriptional responses of dorsal root ganglia cultured in the presence and absence of blastema cells from regenerating salamander limbs
Source: Regeneration (Oxf). 2014 Jul 14;1(2):1–10. doi: 10.1002/reg2.14 (PMC4349419; doi:10.1002/reg2.14)
Supplement: Supplementary file 1 [file REG2-1-01-s001.pdf]

Probe Set ID	GENESYM	ANOVA p-value	p-value	Change D5 v D0	Ratio Day 5/Day0	p-value D10B v D10	Change D10B v Day 10	Ratio D10B/D10
axo00008-f	ADSL	0.002042	0.00173582	1	1.79687037	0.88996104	0	1.01973927
axo00012-r	TP53INP1	0.000121	0.00020745	1	2.17807807	0.87282906	0	1.02263691
axo00051-f	HAUS8	0.000144	0.00299702	1	1.87249907	0.99277664	0	0.99851223
axo00101-f	WDR4	0.000419	0.00026875	1	2.65276645	0.99737944	0	0.99940523
axo00108-f	WDR5	0.003121	0.00127655	1	1.47483204	0.303815	0	0.90971928
axo00150-r	MBP	0.000355	0.00174479	-1	0.52427416	0.85999475	0	1.02786918
axo00158-f	MED8	0.002626	0.00054454	-1	0.48992235	0.74481066	0	1.04867842
axo00180-f	HK2	0.000015	7.31E-06	1	8.47582617	0.98389917	0	1.00518752
axo00217-f	IFT122	0.005716	0.0029636	-1	0.68547851	0.01764224	0	1.31552834
axo00233-f	MYLK	0.001986	0.00041905	-1	0.50609004	0.13596293	0	1.23660751
axo00251-r	GNG2	0.000189	2.75E-05	-1	0.46273956	0.38843391	0	0.90950701
axo00255-r	CLIC6	9.668E-06	8.17E-06	-1	0.21779368	0.96744685	0	1.00758648
axo00270-f	CRYBA2	0.000139	0.42453178	0	1.07137584	0.00176152	1	1.42116165
axo00271-f	LPAR1	0.00001835	1.20E-05	-1	0.51385753	0.61706932	0	1.04346629
axo00277-f	COL6A3	0.000929	0.00020879	-1	0.30155464	0.98938762	0	0.99713909
axo00283-f	NARG1	0.003974	0.00109863	1	1.93377016	0.79105812	0	0.96133844
axo00298-f	PSAT1	0.000531	6.51E-05	1	4.15485367	0.89569328	0	1.02941877
axo00308-r	CCNT2	0.00036	7.58E-05	1	2.59101853	0.75653297	0	0.95428295
axo00310-r	U2AF1	0.000905	0.00063573	1	1.64256485	0.98296756	0	1.00221127
axo00332-r	MYL1	0.00004736	4.96E-05	-1	0.15966969	0.27780345	0	1.36151826
axo00336-f	BANP	0.00466	0.000699	1	2.23181431	0.31265496	0	1.19240142
axo00345-f	PTP4A2	2.855E-06	8.64E-07	1	2.52161846	0.66423154	0	0.96257443
axo00349-f	SDCBP2	0.0059	0.00640581	1	1.74391735	0.43215912	0	0.87623596
axo00407-f	TREX2	1.47E-07	2.40E-07	1	12.0931038	0.83173788	0	1.04458628
axo00408-f	TREX2	0.00005445	4.29E-05	1	3.05902522	0.15003743	0	0.77793634
axo00433-f	SPSB4	0.005064	0.00124584	1	2.19634141	0.22455212	0	0.7960743
axo00457-r	MXI1	0.003784	0.01208491	1	1.38314685	0.98493877	0	1.0020485
axo00475-f	RGS18	0.00008571	8.13E-05	-1	0.29367018	0.79084907	0	0.9493974

axo00513-r	HPRT1	0.005505	0.00110386	1	1.91948595	0.93742326	0	1.01160775
axo00520-f	WHSC1	0.000133	0.00014725	1	2.86945948	0.11859995	0	0.73939183
axo00532-f	YTHDC1	0.001859	0.0010305	1	1.94869639	0.58701415	0	0.92172013
axo00555-f	TTN	0.005348	0.00139606	-1	0.23968308	0.92266166	0	1.03288526
axo00626-f	ZNF483	0.00002943	1.47E-05	-1	0.42208623	0.67343474	0	0.95361996
axo00629-f	FAM108B1	0.000653	0.00024771	1	3.16284897	0.61364009	0	0.89805352
axo00668-f	NUP35	0.000435	0.00052645	1	1.83328744	0.76599231	0	1.03741514
axo00675-f	PYGO2	0.002782	0.00326422	1	1.39382361	0.74171971	0	1.02959955
axo00679-f	JOSD2	0.003754	0.00306913	1	4.36102522	0.96542142	0	0.98333757
axo00746-r	ADAT3	0.001449	0.00316556	1	1.34589077	0.53110705	0	1.05097911
axo00761-f	REP15	0.000743	0.00252493	-1	0.61719859	0.46706131	0	0.91317167
axo00772-r	ZMYND19	0.000698	0.00011559	1	2.71892873	0.59261224	0	0.91404958
axo00775-f	TLCD1	3.617E-06	4.05E-06	1	3.52980707	0.38077273	0	1.13495492
axo00797-r	BCL2L1	2.021E-06	4.73E-07	1	2.86982945	0.27770335	0	0.9007138
axo00809-f	PPP1R14B	0.0000187	2.15E-05	1	6.06358633	0.45984216	0	1.20166131
axo00810-f	C7orf11	0.00017	0.00042199	1	1.94332788	0.47481312	0	0.90947297
axo00832-f	C13orf27	0.003767	0.00173167	-1	0.51569203	0.59616416	0	1.08889537
axo00915-r	MAPKAPK5	0.004532	0.00096485	1	2.01369428	0.26150627	0	0.83549205
axo00926-f	SGSH	0.003197	0.00119823	-1	0.58527477	0.55352637	0	1.07580977
axo00943-f	MTFMT	0.004997	0.00839146	1	1.38253979	0.52916185	0	1.06641847
axo00949-r	MCFD2	0.000511	0.0022379	-1	0.66539134	0.1495996	0	0.85599246
axo00951-r	WDR36	0.001524	0.00024579	1	1.90188454	0.73079759	0	1.04155931
axo00979-f	ANGEL2	0.00004261	0.00001643	1	2.75776846	0.85308057	0	1.02507372
axo00995-f	ATP6V1C2	0.000937	0.00107473	1	2.25857528	0.71326494	0	0.9347592
axo01010-r	CCDC43	0.000137	0.00038868	1	2.38506183	0.25507258	0	1.22103651
axo01042-f	NACC2	0.003631	0.00048981	-1	0.62576824	0.49038811	0	1.06808293
axo01052-f	SESN3	0.00003928	7.4574E-05	-1	0.38288994	0.89894932	0	0.9809356
axo01061-r	ARNTL	0.005477	0.00255631	1	1.58115595	0.47181008	0	0.91809032
axo01084-r	C1orf51	0.00044	0.00016068	1	3.05819415	0.65120753	0	0.91550796
axo01094-f	FAM82A1	0.00078	0.00058834	-1	0.55833659	0.62562645	0	0.94268565

axo01107-f	THAP6	0.003697	0.00095762	1	1.78567765	0.64105757	0	1.06188363
axo01171-r	C11orf66	0.00006724	0.00031385	-1	0.48964656	0.51243637	0	1.09380154
axo01176-f	CCDC11	0.00004125	1.67E-05	1	2.78947573	0.69946144	0	1.05387201
axo01180-f	CCDC46	0.00006422	8.45E-06	-1	0.5355112	0.06837326	0	1.16413981
axo01221-f	SLC2A12	0.000763	0.0002894	-1	0.38396215	0.81411484	0	1.04316377
axo01243-r	ZSCAN20	0.005229	0.00148874	1	2.11239017	0.52324185	0	0.89257584
axo01268-f	IDS	0.000663	0.00025017	-1	0.26572748	0.92704809	0	0.97792225
axo01276-r	RTKN2	0.0000843	3.78E-05	-1	0.43642732	0.78935464	0	1.03277382
axo01279-f	UCMA	0.000215	0.00059532	-1	0.30707721	0.17225579	0	1.41919812
axo01280-f	LACE1	0.004759	0.00261833	-1	0.65221823	0.31275898	0	0.89245459
axo01288-f	SLC15A4	0.000763	0.00138661	1	2.0824454	0.75945493	0	0.94888161
axo01307-f	TIGD3	0.000362	0.00077326	-1	0.57351442	0.62097923	0	0.9424376
axo01312-r	TRAF5	7.92E-08	3.93E-08	1	5.80751376	0.23179611	0	0.86217502
axo01321-f	GSTZ1	0.003812	0.0012953	-1	0.55204776	0.93990041	0	1.01038958
axo01322-r	HMGA1	0.00001582	1.36E-05	1	8.75121905	0.696037	0	1.1159002
axo01328-r	CTSL1	0.001508	0.00036389	1	2.21590598	0.93352983	0	1.01291047
axo01337-f	ZNRF2	0.000236	0.00002579	1	3.59098676	0.79698171	0	0.95528051
axo01345-f	ACOT11	0.000725	0.00012955	-1	0.3536301	0.23451285	0	0.80493582
axo01398-f	FBXO34	0.005221	0.00158424	1	1.6862	0.22646277	0	0.85537385
axo01421-f	OBFC2A	0.00002934	0.00001735	1	3.28260843	0.40636146	0	1.14252468
axo01423-r	TARS	0.00007975	1.08E-05	1	2.7727733	0.67462541	0	0.94746398
axo01440-f	C12orf45	0.000195	0.00012303	1	2.53974503	0.67052935	0	1.06921106
axo01461-r	C1orf93	0.000158	0.00081444	1	1.72271295	0.58940344	0	1.06593047
axo01503-f	ZNF572	0.00001081	3.72E-06	1	4.6139744	0.6250571	0	1.08716808
axo01526-f	RDH12	0.000117	0.00014633	-1	0.56775261	0.44485246	0	0.92735845
axo01556-f	C3orf21	0.000331	0.00203132	-1	0.57310816	0.2154826	0	0.83752278
axo01557-r	SCFD2	0.002234	0.01206871	-1	0.69769835	0.24627633	0	0.86539759
axo01595-r	RNF168	0.005384	0.00345385	1	1.68539646	0.95545121	0	0.99220881
axo01613-f	THAP8	0.000352	4.83E-05	1	2.41624766	0.23512901	0	0.84975744
axo01620-r	OSTalpha	0.003819	0.00135052	-1	0.38216406	0.45725847	0	0.84497779

axo01638-f	POLR1D	0.000874	0.00091436	1	1.65509497	0.46443775	0	1.08549017
axo01648-r	HEPACAM	0.002955	0.00203917	-1	0.51157985	0.75307296	0	1.05358545
axo01694-f	D2HGDH	0.001919	0.00940695	-1	0.64029915	0.79157543	0	0.9630734
axo01696-f	FAM133B	0.000726	0.00043587	-1	0.58680741	0.31499697	0	1.11511255
axo01730-f	RBM45	0.000182	9.92E-05	1	1.90349705	0.72520212	0	0.96356418
axo01752-f	ZNF488	0.001588	0.00191202	-1	0.67104108	0.06280483	0	1.22073778
axo01779-r	TMEM92	0.00000129	9.73E-07	1	10.611843	0.18381486	0	1.37064346
axo01795-f	COL29A1	0.00099	0.0003715	-1	0.35979	0.14946716	0	1.35259038
axo01799-f	SNX33	0.000273	6.18E-05	-1	0.38465279	0.7734566	0	1.04342362
axo01804-r	HYAL1	0.003548	0.00322468	1	1.71542851	0.66182141	0	1.06495588
axo01815-f	RNF19B	0.00001539	3.97E-05	1	2.28507576	0.82195949	0	1.02759492
axo01842-f	MED19	0.00455	0.00350659	1	1.43436282	0.59753585	0	1.05293764
axo01871-f	IKIP	0.000692	0.00020454	-1	0.54966201	0.36745307	0	0.90595021
axo01896-f	C10orf46	0.00171	0.00137288	1	1.54192579	0.43793631	0	1.08266207
axo01920-f	ITGB1BP3	0.004421	0.00493584	1	2.90347981	0.85053595	0	1.05886207
axo01922-r	HNRNPH2	0.005327	0.00255205	1	2.42727348	0.60467119	0	0.88852976
axo01935-f	TGIF1	0.00001972	9.52E-06	1	10.4353205	0.61300332	0	1.1591313
axo01939-r	SGK3	0.00024	0.00046515	-1	0.28120445	0.98865807	0	0.99640396
axo01943-r	RASSF1	9.83E-08	1.02E-07	1	13.7608149	0.87806641	0	0.97023514
axo01959-r	TRIM39	0.005175	0.00261626	1	1.94001795	0.05882652	0	0.70171025
axo01975-r	ABTB1	0.00005565	0.00023476	-1	0.66421521	0.89558289	0	0.99024746
axo02001-f	SPAG1	0.000235	8.54E-05	1	2.62201148	0.80646352	0	0.96275236
axo02028-f	APBB2	0.005842	0.00355525	-1	0.75109814	0.10524328	0	0.8744582
axo02052-f	TGIF1	0.002307	0.00115193	1	3.87173443	0.74227789	0	1.10655334
axo02076-f	MMGT1	0.00087	0.00067349	1	1.49779654	0.45872076	0	1.06591889
axo02081-f	DCUN1D3	0.003299	0.00084735	1	1.79992651	0.46671926	0	0.91013282
axo02083-f	CYP4F22	0.000439	0.00052927	-1	0.53533851	0.32052647	0	0.8784802
axo02086-f	TSHZ2	0.0000465	0.00001621	1	3.85017227	0.91001598	0	1.02024408
axo02095-f	CCDC117	0.000571	0.00021138	-1	0.54533828	0.36299012	0	0.9034978
axo02097-r	GLUL	0.000147	0.00013502	1	2.40653933	0.41731483	0	1.13110014

axo02104-f	VKORC1L1	0.000132	0.00009817	1	2.82855718	0.61538302	0	0.91771261
axo02109-r	GABRG1	0.003954	0.0026612	-1	0.71108569	0.03302164	0	1.23608932
axo02114-f	GTF2IRD2	0.000233	0.0001155	1	2.57880377	0.36944934	0	0.86523224
axo02115-f	GTF2IRD2	0.000502	0.0002318	1	2.29458195	0.79139997	0	1.04089701
axo02126-f	C3orf58	0.000749	0.00321216	-1	0.69058122	0.76283967	0	0.9707493
axo02134-r	EPHX4	0.000349	0.00740985	-1	0.74082156	0.16071935	0	0.8733285
axo02163-f	RNF149	0.001689	0.00108205	-1	0.63058481	0.62181531	0	0.94984784
axo02273-f	CSTF1	0.001089	0.00025704	1	1.67370508	0.4408306	0	0.92825237
axo02277-r	LDLRAD3	0.001359	0.00050337	1	2.19075882	0.64012749	0	1.07730319
axo02292-f	ADCK5	0.000358	0.00022799	-1	0.51369372	0.88821923	0	1.01717036
axo02319-f	C8orf42	0.0000557	1.47E-05	1	2.39094841	0.10606687	0	0.82156066
axo02345-f	LYG2	0.003208	0.01103212	1	2.05052974	0.58231379	0	1.13969859
axo02394-f	MIDN	6.329E-06	1.06E-06	1	3.47544408	0.30475313	0	0.88062742
axo02410-f	SULT1A1	0.003002	0.00250574	-1	0.36558328	0.79251102	0	1.06992945
axo02412-f	SNRPD2	0.000284	0.00014588	1	1.69318154	0.27144814	0	1.10811427
axo02420-f	PPM1B	0.00006576	0.00003066	1	3.61825311	0.9880867	0	1.00272547
axo02462-f	SLC35B2	0.001416	0.00067746	-1	0.51183416	0.49008892	0	0.90622634
axo02467-f	ZNF598	0.00001685	0.00001277	1	2.41086968	0.7094938	0	0.95878816
axo02532-f	WDR69	0.00004357	3.32E-05	1	4.75468028	0.64440041	0	1.1090912
axo02545-f	TIAL1	0.000906	0.00062804	1	2.40761048	0.73081367	0	0.93883552
axo02551-r	SFXN2	0.000175	7.43E-05	1	2.44083764	0.30047752	0	0.86070892
axo02566-f	WDR20	0.000223	3.63E-05	1	3.30633216	0.84229278	0	0.96614034
axo02583-r	KCNMB2	0.00513	0.00266729	-1	0.40423842	0.6258225	0	1.12120079
axo02591-f	ALDH1L2	0.001727	0.00059569	-1	0.35369834	0.20781644	0	0.75407068
axo02595-r	RFC2	0.00001263	0.00012101	1	2.04754288	0.78213406	0	0.96726292
axo02622-f	PRDX1	0.003609	0.00500863	1	1.93316306	0.81271094	0	1.04561615
axo02623-r	PRDX1	0.003736	0.00547321	1	1.95145023	0.73911261	0	1.06679034
axo02624-f	PRDX1	0.000952	0.00141529	1	2.1340096	0.86621703	0	1.03032061
axo02636-f	METTL2A	0.000681	0.00023484	1	1.98440523	0.91920048	0	1.01276702
axo02653-r	DPY19L4	0.000695	0.0011865	1	2.14119027	0.39698591	0	1.16151786

axo02687-f	STARD5	0.001167	0.00045666	-1	0.69986602	0.1287356	0	0.89139102
axo02704-f	ZFAND2A	0.000406	0.00012111	1	2.75532405	0.96933197	0	1.0065536
axo02731-f	BCL9L	0.000278	6.08E-05	1	2.31340416	0.32942583	0	0.87884176
axo02759-r	PCNA	0.00305	0.00427026	1	2.62051312	0.84055012	0	1.05535619
axo02761-r	PCNA	0.000188	0.000325	1	2.20115387	0.78785636	0	1.04132952
axo02762-f	UBE2E1	0.001174	0.00409184	-1	0.56731634	0.28825335	0	0.84276657
axo02772-f	ANKDD1A	0.000038	5.77E-06	1	3.40496193	0.95067534	0	1.00889008
axo02775-f	CREM	1.357E-06	1.04E-06	1	7.06482912	0.23995745	0	1.25855651
axo02780-r	SUMF1	0.000441	0.00011675	-1	0.63305379	0.44824624	0	0.94294618
axo02785-r	SLC6A15	0.001069	0.00016705	1	1.97998523	0.35709902	0	0.89373127
axo02791-f	ATF4	0.00008019	3.15E-05	1	1.67650486	0.17631179	0	1.11001564
axo02795-f	FILIP1L	0.001171	0.00062363	1	2.88510735	0.31166484	0	1.25745404
axo02813-f	HEATR3	0.002245	0.00109972	1	2.60529065	0.4932188	0	0.86099335
axo02821-f	BIRC3	6.64E-07	4.37E-07	1	4.85468268	0.87338201	0	0.97812901
axo02823-f	IRF2BP2	0.002081	0.00106071	1	2.0769541	0.27311136	0	0.83078419
axo02833-r	DLGAP4	0.00002	1.11E-05	1	2.35150652	0.38440441	0	1.10016736
axo02864-r	RLIM	0.00228	0.00101265	1	1.89582877	0.15549043	0	0.80768949
axo02870-r	C6orf145	0.000336	5.8159E-05	1	2.10608744	0.37210111	0	0.90125879
axo02877-f	RNF14	3.82E-08	1.98E-08	1	3.29607712	0.66206556	0	1.03361578
axo02902-r	RBPMS2	0.004778	0.00205089	-1	0.32951644	0.65900585	0	1.12940532
axo02922-r	B3GALTL	0.002116	0.01118587	-1	0.57811283	0.25578875	0	0.80873484
axo02937-f	LBR	0.000221	0.00050287	-1	0.2662215	0.99250326	0	0.9974928
axo02962-f	ACPL2	0.00004926	2.40E-05	-1	0.31446405	0.44282467	0	0.88322342
axo02977-f	AQP1	0.000362	0.00028737	-1	0.25352443	0.34551906	0	0.78011569
axo02988-r	BCO2	0.000237	0.00647612	-1	0.48395965	0.43833265	0	0.84335795
axo03000-r	ING1	0.000988	0.00015363	1	1.63363763	0.87592548	0	1.01333486
axo03013-r	RSRC2	0.000274	0.00010989	1	1.89664105	0.56924594	0	0.94096307
axo03054-f	PDE4B	1.85E-07	6.11E-08	1	4.98657644	0.16555155	0	1.18153579
axo03089-f	KIT	0.000306	0.00036397	-1	0.27104851	0.79534192	0	0.93654226
axo03113-f	FAM89A	0.00002117	4.04E-06	1	4.35776682	0.75629551	0	1.05270655

axo03124-r	VWC2	0.00287	0.00210759	-1	0.48088595	0.5380871	0	0.89315223
axo03170-f	PRICKLE2	0.000101	2.62E-05	1	2.87960847	0.1635945	0	0.80573277
axo03225-r	MKNK2	0.001368	0.00208718	1	1.77651106	0.09829701	0	1.28854537
axo03230-f	TMEM189	0.0000162	1.69E-05	-1	0.45390533	0.64640526	0	0.95297702
axo03238-f	PMEPA1	0.003943	0.00601854	1	1.94406753	0.95929085	0	1.01003713
axo03245-r	BRE	0.000234	0.00015747	-1	0.66265262	0.29292357	0	0.92565639
axo03251-r	MAP1D	0.00005543	8.60E-06	-1	0.51718094	0.3721123	0	0.92916819
axo03262-f	SPRY1	0.001785	0.00759574	-1	0.66644451	0.01992373	0	0.71421153
axo03332-f	PPHLN1	0.001884	0.00268308	1	1.97303653	0.48234217	0	0.88282537
axo03334-f	EGFL7	0.000212	0.00018683	-1	0.51608097	0.05327783	0	1.28504748
axo03340-f	NDRG2	9.399E-06	1.72E-05	-1	0.33908856	0.48336395	0	0.90331099
axo03375-f	RBBP8	0.000732	0.00052667	-1	0.52564936	0.26015945	0	0.85881304
axo03391-f	SHC4	0.004367	0.00175502	1	4.53008148	0.68303712	0	0.86109998
axo03393-f	PDLIM7	0.005254	0.00595948	1	2.492035	0.62937976	0	1.13913442
axo03395-f	C3orf54	0.000133	5.09E-05	-1	0.58371539	0.01184708	1	1.27582902
axo03397-f	ACSL3	0.002365	0.01121001	-1	0.65648517	0.81057779	0	0.96726717
axo03400-r	RBM12B	0.000075	3.01E-05	1	2.0054324	0.55398112	0	0.94291524
axo03402-r	IYD	0.000834	0.00760318	-1	0.62704809	0.12375742	0	1.2647571
axo03408-f	RCAN1	4.48E-07	2.18E-07	1	4.96247557	0.68815818	0	0.948772
axo03422-f	LEP	0.000251	0.00012284	1	37.3525975	0.53897995	0	1.45831563
axo03429-f	KEAP1	0.001251	0.00030089	1	2.9010222	0.81633712	0	1.04779126
axo03441-f	EFEMP1	0.001009	0.00039951	-1	0.41815537	0.82478949	0	0.96288206
axo03447-f	PARD3B	0.00005636	2.27E-05	-1	0.32434977	0.56718034	0	0.91495604
axo03456-f	RASL11A	1.41E-07	9.00E-08	1	8.55546736	0.32245432	0	0.85086805
axo03466-r	QKI	0.000488	0.00014428	-1	0.29733077	0.38828718	0	1.2007263
axo03490-f	SYTL2	0.00009771	7.20E-05	-1	0.41704289	0.11225307	0	1.26325539
axo03573-f	FAM150A	0.000125	0.00044833	1	2.39248465	0.959452	0	0.99122307
axo03584-r	SGCG	0.000605	0.00150457	-1	0.31164749	0.94655901	0	1.01860521
axo03595-f	B4GALT4	0.000406	0.00053339	1	1.79418128	0.40580494	0	0.90417504
axo03643-r	TXNRD3	0.000446	6.3297E-05	1	1.88164787	0.33068813	0	0.90705588

axo03660-f	LOC653884	0.00000566	6.05E-06	1	2.54115316	0.76467632		0	1.03340087
axo03665-f	COL22A1	0.002452	0.63671427	0	0.95259794	0.00801519		1	1.39119791
axo03667-r	LOC653506	0.000926	0.00063973	1	2.7621417	0.56986254		0	1.12920415
axo03672-f	PICK1	0.006023	0.00252984	1	1.74851681	0.5395531		0	1.09241018
axo03686-f	LOC1001335	0.000433	9.68E-05	1	3.72529154	0.87830031		0	0.96768987
axo03736-f	SGCB	0.000152	0.00996294	-1	0.75125955	0.08403689		0	0.84142116
axo03824-r	CREBZF	0.000164	0.00018305	1	1.98967206	0.19519238		0	0.84814882
axo03837-f	LOC1001310	0.00005116	3.69E-05	1	3.27496162	0.87997389		0	0.97435787
axo03846-f	PRMT5	0.002096	0.0021605	1	1.60123753	0.20032954		0	0.85474639
axo03927-f	LOC1001282	3.786E-06	3.15E-06	-1	0.18705546	0.3447017		0	1.19359633
axo03985-r	YIF1B	0.003008	0.009439	1	1.54899269	0.89621359		0	1.0183833
axo03989-f	LOC652595	0.000544	0.0008421	1	2.40035702	0.48965041		0	1.14208762
axo04111-f	LOC255411	0.002399	0.00064236	-1	0.57856595	0.12969639		0	1.20226256
axo04155-r	DEDD	0.000795	0.00019447	1	2.24464454	0.26355925		0	0.84668995
axo04156-f	DEDD	0.00033	0.00013647	1	1.68954878	0.93016135		0	1.00785424
axo04166-r	CCRK	0.000112	0.00041094	-1	0.61375013	0.16098203		0	0.86785144
axo04194-r	C3orf57	0.00006649	0.00785608	-1	0.6396215	0.43119187		0	0.89555276
axo04229-f	PAPD5	0.001349	0.00046016	1	2.8796943	0.73140546		0	0.92993321
axo04237-f	MAOA	0.000378	0.00046914	-1	0.32494685	0.57343873		0	1.13613531
axo04240-f	ZBTB8A	0.000826	9.15E-05	-1	0.49492289	0.98316741		0	0.9976009
axo04262-r	PPAN-P2RY1	0.000244	7.59E-05	1	2.91138776	0.58958826		0	1.09623695
axo04302-r	PSPC1	0.00009568	9.19E-05	1	2.03675625	0.35535498		0	0.89678248
axo04303-f	TFAP2A	0.002103	0.00096981	1	3.06341113	0.18504832		0	1.41082724
axo04310-f	FILIP1L	0.0000492	0.00002717	1	4.21357752	0.43962359		0	1.17093717
axo04316-f	PSAP	0.00002945	0.00002144	-1	0.24570498	0.66257211		0	1.08724092
axo04350-r	FRS2	0.001455	0.00056097	1	1.83050452	0.26826214		0	0.86784923
axo04387-f	C2orf86	0.000833	0.00105756	-1	0.68401791	0.4330543		0	0.93444911
axo04388-f	FAM159A	0.00005127	3.81E-05	1	5.00868078	0.71381761		0	0.91736112
axo04405-f	SLC12A2	0.0015	0.00759976	-1	0.4505763	0.55324694		0	0.86389687
axo04428-f	RCC1	0.000357	0.00045368	1	2.11056267	0.91492661		0	0.98423541

axo04436-r	DBNDD2	0.002508	0.0032395	1	2.4240722	0.83992711	0	1.04867551
axo04453-f	TNFRSF1A	0.00005449	0.00013147	1	2.09460373	0.90806795	0	0.98563815
axo04476-f	ZNF391	0.001266	0.00045058	-1	0.57252066	0.71601699	0	1.04132591
axo04495-f	ZNF436	0.000608	0.00042422	1	2.16076923	0.49627624	0	1.1103727
axo04536-f	HNRNPC	0.003939	0.00754764	1	1.32144832	0.50518053	0	1.0592477
axo04542-f	NFKB2	0.00001967	2.08E-05	1	4.07894108	0.54603116	0	0.89044187
axo04548-f	SLC39A7	0.000111	0.00016158	1	1.69130088	0.44720704	0	0.93190836
axo04561-f	SFRS1	0.004551	0.00204468	1	2.6919644	0.65644787	0	0.89638889
axo04570-f	CDC16	0.001224	0.00090529	-1	0.64598534	0.74308077	0	0.96902021
axo04579-f	KTN1	0.0000883	8.41E-05	-1	0.43824828	0.08274295	0	1.28250075
axo04597-f	GLB1	0.001676	0.00114272	-1	0.49581518	0.77398503	0	0.95529177
axo04611-r	LAMA2	0.0000106	7.84E-06	-1	0.2404612	0.68047123	0	0.93119099
axo04673-f	KDM6B	0.00004874	2.22E-05	1	4.79444117	0.7169482	0	1.08092218
axo04679-r	PEX6	0.000805	0.00089887	-1	0.59449188	0.19076881	0	0.85562013
axo04711-r	ZNF697	0.000302	0.0015327	-1	0.55773318	0.01109068	-1	0.65701794
axo04719-r	PFKM	0.00275	0.00337498	-1	0.2835893	0.97523267	0	1.01050981
axo04739-f	CLEC2L	0.003649	0.00085851	-1	0.54114908	0.13802189	0	1.23388135
axo04785-f	JMJD6	0.004376	0.00477586	1	2.32370082	0.84122876	0	1.04899902
axo04812-r	CASC1	0.00003341	1.2064E-05	-1	0.44454191	0.32172721	0	1.11047917
axo04824-r	C6orf182	0.005518	0.00138374	1	1.98454645	0.22392462	0	0.81705608
axo04834-f	EARS2	0.001666	0.00038161	1	1.63829028	0.28652712	0	0.89988507
axo04865-f	MRAS	0.002512	0.00048808	1	2.44811953	0.72758048	0	0.93902838
axo04891-f	PMP22	0.000291	0.00043685	-1	0.16085905	0.31500621	0	0.68826259
axo04892-f	ABCF1	0.003475	0.00668227	1	1.5281922	0.57833853	0	0.93124757
axo04905-f	ACTA1	0.001995	0.00106791	-1	0.26146366	0.4865594	0	1.23627269
axo04914-f	FUT2	0.002709	0.00218355	-1	0.60551545	0.22125256	0	0.85266385
axo04964-r	TXNDC2	0.00006317	0.00005462	1	1.76715265	0.64510631	0	0.96072582
axo04976-f	C1orf43	0.00058	0.00020151	1	1.77967031	0.74547389	0	0.96699679
axo04978-f	OBSCN	0.000786	0.00044354	-1	0.66492829	0.64924961	0	0.96363943
axo04979-f	MID1	0.000248	0.00018897	1	2.47497966	0.93155161	0	1.0139056

axo05046-f	ACTN1	0.002954	0.0073798	1	1.89558349	0.85241014	0	1.03699409
axo05057-f	HIGD1A	0.000534	0.00028304	1	1.72084442	0.84327687	0	0.98011753
axo05064-r	GOLSYN	0.004461	0.00348143	1	1.5322443	0.01252214	0	0.71137245
axo05068-f	CYP4B1	0.000159	0.00044755	1	1.82054113	0.98131071	0	0.99721489
axo05148-r	MED7	0.000373	0.00083448	1	1.82162374	0.86115586	0	1.02299612
axo05178-f	C14orf159	0.001734	0.00047027	-1	0.54248344	0.66847251	0	0.94871062
axo05186-r	C8orf41	0.005258	0.00704375	1	1.47231769	0.56601886	0	1.07010251
axo05187-f	ZFAND5	0.000343	0.00012797	1	1.77125673	0.44252839	0	1.07818395
axo05194-r	SNX18	2.919E-06	4.42E-06	1	2.96640175	0.98916627	0	1.0016742
axo05207-f	NTF3	0.000156	0.00016314	1	4.26288952	0.45290167	0	0.8250505
axo05214-f	ELOVL7	0.00003948	2.72E-05	-1	0.16920503	0.66784113	0	0.89848874
axo05220-r	PDE4D	0.00079	0.00070493	1	1.90560222	0.44779767	0	0.90021323
axo05221-r	PDE4D	0.003289	0.00107308	1	2.61664583	0.7624294	0	0.93666171
axo05223-f	SMN1	0.004539	0.00303518	1	2.6779778	0.99417923	0	1.00188969
axo05253-f	MYBBP1A	0.000236	0.00004182	1	2.31778755	0.61279604	0	1.06503585
axo05267-f	UGT2A2	0.00009819	0.0002913	-1	0.24842249	0.2499939	0	0.73236394
axo05305-r	CCNA1	0.00054	0.0076201	1	1.78380552	0.42633226	0	0.86619835
axo05309-r	DDX54	0.00007527	4.17E-05	1	2.60249489	0.83886477	0	0.97177665
axo05328-f	BRD2	0.002023	0.00098879	1	2.30705969	0.7372682	0	0.93950039
axo05333-r	RP11-544M2	0.00595	0.00990066	1	1.95650211	0.56082165	0	1.13514243
axo05362-f	TGM1	0.000181	0.00038112	1	2.56399006	0.11903042	0	1.35638776
axo05376-r	SLC44A3	0.002303	0.00296314	-1	0.52875183	0.4208753	0	0.87234497
axo05428-f	GLRX	0.000381	0.00322989	1	1.38221304	0.88152726	0	0.98727461
axo05429-r	ADFP	0.000289	0.00038661	-1	0.24126474	0.89972229	0	0.96567882
axo05430-f	ADFP	3.08E-07	7.94E-08	1	10.0726729	0.69976392	0	1.06754977
axo05449-f	NCOA7	0.001562	0.00144594	1	2.64280443	0.66433843	0	0.9054007
axo05461-f	ZBTB1	0.000703	0.0002008	-1	0.3362223	0.24807392	0	0.79194218
axo05465-f	BCOR	0.00002165	8.10E-06	1	4.38193778	0.84940385	0	0.96648908
axo05468-r	CLDN19	0.00003145	0.00002335	-1	0.17767029	0.63024078	0	1.12171487
axo05517-f	CEP55	0.001248	0.00176676	1	1.6423918	0.79896994	0	0.96988191

axo05557-f	ACADM	0.0000385	0.00003855	-1	0.33722483	0.46316844	0	0.88883262
axo05558-r	ACADM	0.00028	0.00037037	1	1.96144018	0.9955504	0	0.99927246
axo05562-f	NR2C1	0.003972	0.00404313	1	1.60649631	0.10235756	0	0.79525788
axo05587-f	ZNF84	0.004003	0.00073299	-1	0.64188941	0.46781123	0	0.93293534
axo05597-r	ABAT	0.003227	0.12783858	0	1.19960824	0.00214616	-1	0.63691599
axo05604-f	FLNC	0.000191	0.00029037	1	2.62599833	0.57490969	0	1.1078442
axo05621-r	FAM189A2	0.000347	0.00105084	-1	0.44220789	0.17548061	0	0.77108742
axo05676-f	UGT8	0.001294	0.00085812	-1	0.07004525	0.39580558	0	0.60629689
axo05697-f	C13orf31	0.000238	0.00034068	1	3.51465886	0.80761242	0	1.06048803
axo05706-f	ABCC2	0.000555	0.00017591	1	2.49419685	0.84419238	0	0.96889104
axo05735-f	RRAD	5.915E-06	9.85E-07	1	9.9257125	0.17638197	0	0.73155082
axo05742-r	MARK3	0.0009	0.00031779	1	1.73537767	0.05810315	0	0.80355889
axo05745-f	LRRC32	0.005416	0.00239831	1	5.97525383	0.38826447	0	0.67147581
axo05782-f	CLK3	0.000097	0.00006101	1	1.69789511	0.92594932	0	1.0076072
axo05786-f	CRYZ	0.003913	0.00239347	-1	0.54034922	0.74100814	0	1.05299384
axo05789-f	DOCK9	0.000667	0.00393436	-1	0.48099751	0.23870221	0	0.78254177
axo05801-f	MOV10	9.094E-06	9.33E-06	1	2.72402385	0.96127015	0	1.00602117
axo05813-f	NR1H3	0.001053	0.00091381	1	2.28555996	0.19366842	0	1.2797497
axo05819-r	FAM108A1	0.001198	0.00211922	1	1.80424718	0.54688501	0	1.09325277
axo05844-r	SSFA2	0.000459	0.00131892	1	1.72054107	0.64414567	0	1.06004438
axo05876-r	IPCEF1	0.003534	0.00799544	-1	0.67394153	0.96221977	0	0.99423215
axo05885-f	DNMT1	0.005847	0.00102977	-1	0.60557715	0.17331997	0	0.85196594
axo05886-f	DNMT1	0.000443	0.00009019	-1	0.46404512	0.06082914	0	0.77415356
axo05895-f	ZNF286A	0.004506	0.00518831	-1	0.70956201	0.07624316	0	0.82686784
axo05906-r	TMEM201	0.00002948	1.51E-05	1	4.95011142	0.82598985	0	1.04699581
axo05907-r	ERVWE1	0.00132	0.00109984	1	1.69256394	0.01297496	0	0.704768
axo05926-f	HYOU1	0.000871	0.00047908	1	2.12412156	0.39336863	0	0.87678924
axo05948-f	RFESD	0.005189	0.00612878	-1	0.62755175	0.66197968	0	0.94134796
axo05953-f	TMEM106B	0.002962	0.00220935	-1	0.57026866	0.36245966	0	0.87746714
axo05982-f	LEPREL1	0.000297	0.00085945	1	2.30213377	0.89043584	0	1.02530775

axo06014-r	KRT17	0.00005421	0.80514036	0	1.07264003	0.00002438	1	7.82428723
axo06018-r	SARDH	0.001224	0.00019123	-1	0.58980325	0.70158916	0	1.03673895
axo06129-f	DNAJB5	0.00002541	1.16E-05	1	5.04369371	0.68448112	0	0.91985774
axo06152-f	DPF1	0.005482	0.00341746	-1	0.675304	0.08052103	0	0.8190401
axo06161-f	ZDHHC3	0.00116	0.00027962	1	2.27672441	0.82008655	0	0.96563218
axo06177-f	NDRG1	0.000023	0.00004668	-1	0.2101163	0.95475006	0	1.01328453
axo06178-f	NDRG1	0.000637	0.00014691	-1	0.49877659	0.71366599	0	1.04498169
axo06213-f	OXCT1	0.003478	0.01029135	-1	0.59843799	0.28805315	0	0.83340269
axo06225-f	PENK	0.00019	7.6365E-05	1	2.73863753	0.37509166	0	1.15539621
axo06236-r	TP53INP1	0.001449	0.002784	1	2.04230972	0.93069213	0	1.01622745
axo06315-f	C10orf32	0.00172	0.00150313	-1	0.54258623	0.60448096	0	0.92754038
axo06327-f	LITAF	0.00007949	0.00037595	1	1.75930222	0.33222507	0	0.89670168
axo06334-f	TEK	0.000131	0.00012238	-1	0.29789538	0.06302169	0	1.51476256
axo06397-r	ARFGAP3	0.000175	3.65E-05	1	2.26445102	0.86111254	0	1.02092956
axo06402-f	GTF2H1	0.000363	0.00136143	1	2.12205248	0.89791387	0	0.97780657
axo06466-f	CLP1	0.002486	0.00257917	1	1.62239038	0.42914409	0	1.10473704
axo06473-f	SPHK1	0.001403	0.00047057	1	1.69757267	0.03498095	0	0.7770184
axo06493-f	CHID1	0.001847	0.00227922	-1	0.60044028	0.07756219	0	0.78181581
axo06546-r	BDNF	0.000011	5.03E-06	1	9.87702344	0.64700377	0	1.12892006
axo06567-r	TCTN2	0.000981	0.00228791	-1	0.66852512	0.04217557	0	0.79431345
axo06578-f	TRAFD1	0.00007	2.07E-05	1	1.95591725	0.53607638	0	0.94484964
axo06589-f	WEE1	3.656E-06	6.12E-06	1	3.27248066	0.12963867	0	1.2520134
axo06594-f	F10	0.003777	0.01087371	-1	0.37114813	0.39066396	0	1.32870147
axo06611-f	NAT10	0.00568	0.00113376	1	1.73243833	0.35647785	0	0.88932481
axo06643-f	CCNL2	0.001133	0.00055907	1	5.44122568	0.86854202	0	1.05915348
axo06664-r	MX1	0.004835	0.00299371	1	4.25971724	0.65140927	0	0.84150281
axo06677-f	RAB34	0.003818	0.0063677	1	3.10195265	0.61651712	0	1.18484554
axo06701-f	NEK6	0.001279	0.00027399	-1	0.5651693	0.35907106	0	0.90517125
axo06706-f	HBA2	0.004117	0.01002344	-1	0.26112455	0.01348634	0	3.55581845
axo06707-f	EDEM2	0.000194	5.06E-05	1	2.22936576	0.31788765	0	1.13179787

axo06725-r	NR2F2	0.000206	0.00010076	-1	0.31675633	0.31312998	0	0.82267224
axo06758-f	CDK6	0.00002653	1.32E-05	1	4.18527571	0.46106634	0	1.1474055
axo06759-r	CDK6	0.000592	0.00418574	1	1.66826908	0.81322468	0	1.03408373
axo06810-r	YPEL3	0.003357	0.00244009	-1	0.63244006	0.65825264	0	0.9496034
axo06812-f	RAI14	0.003906	0.00147755	1	2.22208551	0.08133721	0	0.70078283
axo06832-f	LYAR	0.000185	0.0001268	1	2.04098842	0.72623312	0	1.04315967
axo06840-f	ROBO1	0.000117	0.00008517	1	4.23014759	0.76567331	0	1.07156795
axo06859-f	MKI67	0.001804	0.00024476	-1	0.32406355	0.60612884	0	0.89823697
axo06876-f	SNCA	0.0000582	0.00030537	-1	0.36938265	0.2564599	0	1.24719035
axo06885-f	PARP9	0.002971	0.00257597	1	1.78037482	0.60420329	0	0.92580505
axo06886-f	PLP1	0.0000724	0.00006621	-1	0.16590715	0.54502894	0	1.18619566
axo06887-f	PARP9	0.003471	0.00159375	1	2.2759744	0.30484169	0	0.81343727
axo06906-f	AIFM3	0.000278	0.00046121	-1	0.48811387	0.30049408	0	1.16470937
axo06909-f	ZNF674	0.000233	0.00060293	1	1.49561862	0.70977009	0	0.96935925
axo06918-f	AGXT2L1	0.00006843	9.78E-06	1	13.7407451	0.7970474	0	1.08733262
axo06938-f	ANXA11	0.005952	0.0018458	-1	0.48387814	0.8831695	0	0.97437566
axo06939-f	RFXAP	0.000234	0.00055537	-1	0.5946129	0.43279018	0	0.91867067
axo06947-f	HDAC10	0.000783	0.00039318	-1	0.42109374	0.12756937	0	0.76031175
axo06995-f	BCAT2	0.000806	0.0002106	1	2.15536868	0.40520034	0	1.124261
axo07001-f	BMP2	5.636E-06	5.39E-05	1	2.68775506	0.01520348	0	1.53562363
axo07014-r	C1QBP	0.001226	0.00090049	1	1.68426677	0.85111893	0	0.97878688
axo07054-f	CDK9	0.000015	2.32E-05	1	2.74261351	0.56676369	0	1.08324632
axo07075-r	CIRBP	0.002374	0.00317611	1	2.59846927	0.0993219	0	1.56566512
axo07100-f	CLDN7	0.00000163	2.72E-06	1	10.2206568	0.16994228	0	1.43357263
axo07145-f	DHX15	0.002146	0.00057347	1	2.18765666	0.0375869	0	0.68606095
axo07152-f	DKC1	0.000222	0.00010686	1	2.24074633	0.56710432	0	1.07989671
axo07205-f	IL1B	0.000182	0.00017232	1	35.19758	0.24873432	0	2.10884491
axo07213-f	S1PR1	2.219E-06	5.54E-06	-1	0.29072197	0.07228675	0	1.32551684
axo07231-f	SLC11A1	0.000035	6.31E-05	1	6.19257885	0.29213771	0	1.35792927
axo07244-r	ELAVL1	0.00022	0.00041835	1	2.5559058	0.87901143	0	0.97224692

axo07245-r	ELAVL1	0.00014	0.00002539	1	3.46422187	0.39268694	0	1.1619535
axo07247-r	EMP2	0.000628	0.00020084	-1	0.33495983	0.57157658	0	1.11802827
axo07251-f	GPX1	0.00001029	7.75E-06	1	3.18456577	0.27477419	0	1.17083951
axo07263-r	FBL	4.081E-06	1.13E-06	1	3.66347558	0.99078601	0	1.0014613
axo07273-r	FAAH	0.000503	0.0007447	-1	0.19546606	0.94854022	0	1.02271063
axo07287-f	FHL1	0.004398	0.00145456	-1	0.43008081	0.16946223	0	0.75106656
axo07306-r	IL8	1.026E-06	1.02E-06	1	91.0134379	0.35296823	0	1.51127199
axo07325-f	GCLC	0.001136	0.00040738	1	2.54978511	0.31638746	0	0.82777849
axo07333-f	GSTA4	0.003114	0.00809878	-1	0.66199352	0.70304767	0	1.05018003
axo07335-r	GTF2B	0.000349	0.00017317	1	2.24069352	0.47788511	0	0.90321895
axo07379-f	IL10RA	0.000173	0.00012152	1	6.50375991	0.73165576	0	1.11406494
axo07381-f	ING2	0.000043	9.15E-06	1	2.38139565	0.53905078	0	1.06850303
axo07382-f	CXCL10	0.00008827	0.00008848	1	7.75923274	0.48446271	0	1.26354759
axo07395-f	SERPINE1	0.000585	0.00054936	1	7.56517436	0.84319781	0	0.9213884
axo07456-r	APCS	0.00397	0.00570158	-1	0.54093894	0.81016931	0	1.0440417
axo07473-f	AREG	0.000166	0.02721338	0	1.56636923	0.00114857	1	2.18894848
axo07476-f	ARF4	0.00001545	9.58E-06	1	2.18784419	0.931187	0	0.99166861
axo07479-f	ARF6	6.426E-06	6.80E-06	1	2.31143693	0.62045276	0	1.05093978
axo07486-f	NCAM1	0.000683	0.00079566	-1	0.35335486	0.79481307	0	0.94336305
axo07488-f	NCAM1	0.00001961	0.00001221	-1	0.31788077	0.20139332	0	1.21490598
axo07491-f	ATP12A	3.93E-08	6.93E-08	-1	0.20781279	0.86747146	0	0.9812754
axo07521-r	BCL6	0.003476	0.00105417	1	4.12959033	0.12913299	0	0.59879441
axo07528-r	BICD1	0.001394	0.00069365	1	2.99847442	0.94840094	0	1.01511112
axo07565-r	CAPNS1	0.004555	0.00077028	-1	0.59708297	0.23685138	0	0.8731673
axo07582-r	CD63	0.00007344	4.60E-05	1	1.68545331	0.90378952	0	0.99065182
axo07584-r	IL8RA	0.002351	0.00248133	-1	0.52867145	0.40107266	0	1.14889344
axo07599-f	GSR	0.00003368	2.80E-05	1	2.7146239	0.42857432	0	0.89341352
axo07601-r	CDH11	0.001509	0.00324259	-1	0.62885646	0.36615418	0	0.89248573
axo07603-f	CDK7	0.000259	0.00016953	1	2.39774295	0.98198267	0	0.99655108
axo07606-f	CDO1	0.00001124	3.99E-06	-1	0.20661038	0.61774597	0	0.91508268

axo07622-f	CHN1	0.004213	0.0022781	1	4.35976589	0.85676963	0	0.93538098
axo07625-f	CKM	0.000434	0.00017724	-1	0.16546083	0.11489929	0	1.70523748
axo07628-f	CNN3	0.001886	0.00162363	-1	0.62416311	0.39640056	0	0.90737071
axo07638-f	COL2A1	0.000786	0.00047423	-1	0.24751731	0.27045459	0	1.37486885
axo07643-f	COL4A1	0.001748	0.00054808	-1	0.41144457	0.96526811	0	0.99213155
axo07662-r	SLC31A1	0.004539	0.00412312	1	1.5018076	0.72977877	0	0.961843
axo07671-f	CPA1	0.004168	0.0006703	-1	0.42363568	0.46475194	0	1.14311879
axo07673-f	TGFB1	9.50E-07	6.82E-07	1	5.49598983	0.8898753	0	1.0219838
axo07679-f	CPT1A	0.000579	0.00270574	-1	0.36726942	0.89736625	0	0.96720415
axo07691-f	CSNK2A1	0.002225	0.0004889	1	1.75502713	0.50457097	0	0.92645812
axo07699-r	CTGF	0.000419	0.00013248	1	7.1723058	0.92628253	0	1.03139479
axo07719-f	GADD45A	0.002476	0.0023393	1	2.07135668	0.96105555	0	1.0090078
axo07739-r	HBEGF	0.00001112	7.66E-06	1	3.57381264	0.95189846	0	1.00931045
axo07740-f	DUSP7	0.00002432	1.33E-05	1	4.90751723	0.5533809	0	0.88486136
axo07747-r	E2F4	0.000156	0.00007824	1	5.22657554	0.8730087	0	1.04288783
axo07756-f	EGR1	0.00003	2.45E-05	1	5.38079745	0.7641315	0	1.07234565
axo07767-f	EIF4E	8.256E-06	1.97E-06	1	2.49801707	0.86822158	0	0.98438415
axo07783-f	EVPL	0.000083	0.00025337	-1	0.2904412	0.23577858	0	1.32373377
axo07824-r	FLT1	0.000222	0.00014505	-1	0.42185419	0.23243777	0	1.20157997
axo07829-r	AFF2	0.00524	0.0021601	-1	0.58164266	0.9835584	0	1.0027857
axo07832-r	FRK	0.000013	5.41E-06	1	3.27749898	0.96757775	0	0.99442192
axo07843-r	KDSR	0.002563	0.00679566	-1	0.68472187	0.17792945	0	0.85124324
axo07844-r	FYN	0.000439	0.00011576	-1	0.29093256	0.34855848	0	1.2183427
axo07846-r	GAB1	0.001477	0.00188613	-1	0.42995562	0.88877946	0	0.97159435
axo07856-r	GBP1	0.004322	0.00228454	1	3.76752391	0.49503005	0	0.79434446
axo07858-f	GBP1	0.003199	0.0008066	1	4.54948634	0.35726607	0	0.73529071
axo07863-r	GFAP	0.00026	0.00158728	-1	0.59998874	0.87514472	0	0.9810852
axo07868-f	GCLM	0.00005361	5.10E-05	1	2.10809517	0.60559604	0	0.94323555
axo07903-r	GSPT1	0.003732	0.0017865	1	1.52146731	0.32418165	0	0.9023381
axo07920-r	HCK	0.00009476	0.00008552	-1	0.42843582	0.17157218	0	1.21605291

axo07926-f	HLA-DPB1	0.002901	0.00060048	-1	0.30678716	0.56857834	0	0.86887066
axo07929-r	HMOX1	1.82E-08	8.04E-09	1	20.6786199	0.8368352	0	1.03651429
axo07938-r	ALDH2	0.0000661	0.00014947	-1	0.63607259	0.19113702	0	0.89888181
axo07972-f	IL6ST	0.001245	0.00029575	1	2.22818136	0.04040757	0	0.70716233
axo07978-r	IRF1	4.84E-08	2.88E-08	1	13.5284189	0.96245728	0	1.00808224
axo07979-r	IRF2	1.419E-06	2.18E-06	1	3.50825513	0.79228468	0	1.03527928
axo07980-r	IRF2	2.55E-08	5.15E-08	1	3.29282232	0.64860414	0	0.96259
axo07981-f	ISL1	0.000745	0.00316704	-1	0.30740318	0.40679663	0	0.76827373
axo08026-r	KCNJ10	0.00002277	1.30E-05	-1	0.31419933	0.91491522	0	1.01598235
axo08037-f	KPNA2	0.00002881	9.32E-06	1	2.66390629	0.75682964	0	0.96317732
axo08041-r	KPNA4	0.000837	0.00038626	1	1.96229236	0.46090514	0	0.90642528
axo08052-f	KRT15	0.005636	0.03185163	0	0.6262195	0.00264234	1	2.11148744
axo08062-f	LAMB2	0.003362	0.00571317	-1	0.32133484	0.78567667	0	0.91373201
axo08067-r	LAMP2	0.001382	0.00025254	-1	0.50827306	0.80746509	0	0.97003699
axo08080-r	LECT2	0.001505	0.00059204	1	2.33710033	0.86242762	0	1.0308597
axo08094-f	LMO1	0.000241	0.00128052	-1	0.56506536	0.62442061	0	0.93727437
axo08099-f	LOXL2	0.001718	0.00423253	1	2.60460633	0.90430414	0	0.96858037
axo08111-f	LRP1	0.00157	0.0003992	-1	0.38459431	0.74228325	0	1.06360269
axo08115-f	LRP5	0.001089	0.00023551	-1	0.53227425	0.78132304	0	1.03253045
axo08122-f	EPCAM	0.003232	0.00230479	1	6.792092	0.14650772	0	2.09768638
axo08123-f	EPCAM	0.00179	0.00129247	1	6.06045593	0.10174594	0	2.0776846
axo08129-f	MARCKS	0.003205	0.00567365	-1	0.67042615	0.00976762	1	1.43744225
axo08144-f	MAS1	0.000684	0.00028129	1	4.39623549	0.34432925	0	1.30718621
axo08156-r	MDM2	4.09E-08	5.23E-08	1	9.52821522	0.76820737	0	0.95450611
axo08173-f	MIF	0.00071	0.00064454	-1	0.67818307	0.77327137	0	0.97685071
axo08175-f	MMP1	0.001449	0.0026032	1	9.70020117	0.5249889	0	1.45404979
axo08178-f	MMP3	0.000208	0.00174682	1	10.1357715	0.02433407	0	4.25108665
axo08180-f	MMP3	0.00008342	0.00017166	1	63.6707123	0.31548791	0	2.11798445
axo08181-f	MMP3	0.00019	0.00264908	1	8.53308844	0.04963672	0	3.32859959
axo08187-f	MMP19	0.00001	4.30E-06	1	9.23335955	0.95266737	0	1.01501473

axo08238-r	MYH3	0.000171	9.17E-05	-1	0.44847651	0.25813794	0	1.16408064
axo08250-f	MYH11	0.000399	0.00023764	-1	0.41679898	0.94390275	0	0.98880984
axo08251-f	MYH11	0.001087	0.00088359	-1	0.22466745	0.18234102	0	1.57784957
axo08275-r	NEK2	0.000577	0.00018287	1	4.01095098	0.37609254	0	0.80111356
axo08280-r	NFKBIB	0.00001171	3.11E-05	1	1.71551689	0.11734301	0	1.13680121
axo08295-f	NRAS	0.00074	0.00046139	1	1.98601979	0.28656753	0	0.86036257
axo08300-f	NUP88	0.00009863	4.06E-05	1	1.67502156	0.85455195	0	0.98622496
axo08309-f	ODC1	0.005986	0.00164507	1	2.35622231	0.91161514	0	1.02300746
axo08338-r	PAK1	0.00004185	0.00006904	1	5.59234358	0.62512719	0	0.8760558
axo08357-r	PDCD2	0.002186	0.00139874	1	1.51774	0.32407385	0	0.90617044
axo08386-r	CALCB	0.000012	5.31E-05	1	2.35657239	0.10776295	0	1.25094353
axo08389-f	PGM1	0.002872	0.00178938	-1	0.63305379	0.81858055	0	1.02574586
axo08392-f	PI3	0.00000048	3.16E-07	-1	0.13290249	0.77995601	0	1.04904435
axo08428-f	5-Sep	0.005746	0.00315379	1	3.22798826	0.82718203	0	0.93453331
axo08440-f	PPAT	8.681E-06	5.93E-06	1	2.45352505	0.94810601	0	1.00686714
axo08451-f	PPP1CC	0.000118	5.35E-05	1	2.2711138	0.74942181	0	0.96097002
axo08463-f	CHRNA5	0.000743	0.005443	-1	0.56059003	0.2228785	0	0.80867262
axo08487-r	MAPK6	0.000316	5.0647E-05	1	2.42158224	0.8876716	0	0.98135929
axo08491-f	MAPK13	8.941E-06	8.09E-05	1	2.50094269	0.45537189	0	1.11707393
axo08496-r	PRPS1	0.004454	0.000818	1	1.7370027	0.15797426	0	0.83722463
axo08499-f	PRPSAP2	0.00348	0.00078463	1	1.84055603	0.34848322	0	0.88186435
axo08507-r	PSMA1	0.000145	7.10E-05	1	3.89190996	0.95155986	0	1.01303475
axo08514-f	PSMA7	0.002397	0.00232106	1	1.45810371	0.77783928	0	1.02722105
axo08542-r	PSMD8	0.001067	0.00063545	1	1.67789604	0.87663091	0	1.01690154
axo08546-f	PSMD12	0.000776	0.00030954	1	1.87615194	0.66361199	0	0.94930264
axo08549-f	PTBP1	0.000172	5.07E-05	1	3.18323059	0.3020212	0	0.83098442
axo08602-f	PVALB	0.000262	0.00020251	-1	0.08999581	0.56774774	0	1.28194882
axo08603-f	PVALB	0.00003932	0.00003231	-1	0.10387572	0.46889191	0	1.26773218
axo08631-r	RAD23B	0.002898	0.00520306	-1	0.69648909	0.16727765	0	0.85989932
axo08646-f	RASA1	0.0019	0.00078804	1	3.76592256	0.50258479	0	0.82425159

axo08657-r	RBMS1	0.000196	8.23E-05	1	2.51805652	0.40945616	0	0.88345793
axo08666-r	RDX	0.000318	9.4533E-05	-1	0.57671882	0.54998737	0	0.94744887
axo08667-f	RDX	0.000682	0.00032619	-1	0.47864104	0.4585002	0	0.89980877
axo08686-r	RGS2	0.00006216	2.84E-05	1	4.0287157	0.45795951	0	1.15884424
axo08695-r	RGS16	0.000295	0.00011746	1	2.92145891	0.61399193	0	0.91310057
axo08714-r	RYK	0.00062	0.00018314	1	1.93699335	0.73722735	0	0.96145506
axo08720-r	SAFB	0.001467	0.0003417	1	2.05560111	0.27124713	0	0.8547533
axo08721-r	SAFB	0.005076	0.0024551	1	1.74380413	0.64463215	0	0.9365665
axo08731-f	SAT1	0.000162	3.5376E-05	1	1.61580153	0.6896463	0	1.02813283
axo08744-f	SDC4	2.25E-08	1.83E-08	1	43.4398736	0.93170075	0	0.97997035
axo08757-f	SFRP2	0.004335	0.00185436	-1	0.30371716	0.41832323	0	1.26979036
axo08759-r	SFRP4	0.003854	0.00363704	-1	0.31944268	0.76049915	0	0.9099953
axo08763-f	SFRS3	0.000982	0.00093842	1	1.80412171	0.69938677	0	1.05166606
axo08764-f	DHFR	0.00001571	4.45E-06	1	2.68964419	0.62912845	0	0.9469348
axo08768-f	SH3BGRL	0.00005648	0.00032026	-1	0.57194038	0.46237408	0	0.92389662
axo08825-f	SNRPA1	0.001469	0.00217788	1	2.74222446	0.60259165	0	1.14105105
axo08834-f	SOAT1	0.00001011	1.04E-05	1	5.55933762	0.43940708	0	0.84505804
axo08835-f	SOD3	0.001258	0.00136748	-1	0.29538415	0.90929795	0	0.96819709
axo08844-f	UAP1	0.002514	0.00064709	1	2.21894053	0.43916411	0	0.87727253
axo08845-f	UAP1	0.000421	0.00012481	1	2.92266809	0.73501783	0	1.06318157
axo08850-f	GABRA4	0.00004955	0.00034903	-1	0.49153373	0.76446941	0	0.95983323
axo08870-f	CETP	0.001528	0.00437896	-1	0.411396	0.77781819	0	1.07254115
axo08880-r	SSB	0.00005482	1.33E-05	1	2.05561488	0.75116415	0	1.0298803
axo08897-f	STX5	0.000302	0.00009905	1	2.30415324	0.86558334	0	1.02338335
axo08961-f	TGFBR2	0.003958	0.00084949	-1	0.59647848	0.30861833	0	0.8892005
axo08967-f	THBS1	3.865E-06	1.57E-06	1	26.0057727	0.99001562	0	1.00412509
axo08969-f	THBS2	0.00009425	0.00048787	1	3.33922969	0.87398589	0	1.03922161
axo08979-f	TIMP1	4.50E-08	5.81E-08	1	12.3540742	0.30495231	0	1.20630941
axo08984-f	GAS6	0.000141	0.00063975	-1	0.65177022	0.01166068	-1	0.76405655
axo09003-f	GGCX	0.001909	0.00051059	-1	0.44900103	0.09259318	0	0.745136

axo09013-r	TNNI2	0.00007717	7.03E-05	-1	0.32454243	0.30607602	0	1.20392735
axo09045-f	PHLDA2	0.004067	0.00391147	1	5.24410292	0.98057259	0	1.01110559
axo09071-r	UBE2D2	4.02E-07	2.79E-07	1	2.88293172	0.95500562	0	1.00500778
axo09083-r	UGCG	0.00062	0.00009553	1	3.17319607	0.61375262	0	0.90892931
axo09084-f	UGDH	0.001501	0.00185564	1	4.13132722	0.38706762	0	1.35617623
axo09097-r	VAV2	0.00427	0.0024871	1	1.98167906	0.49101227	0	0.88563982
axo09110-f	VIM	0.00002988	0.00009434	1	6.55500554	0.67835152	0	0.88049314
axo09121-f	XPO1	0.000142	0.00039582	1	1.65901738	0.43913644	0	0.92516954
axo09136-r	ZIC1	0.002057	0.00052898	1	2.19474957	0.03963519	0	0.69121096
axo09137-f	ZNF268	0.001357	0.00225188	-1	0.54458104	0.26144621	0	0.83779857
axo09139-f	ZNF268	0.005851	0.00215306	-1	0.637532	0.13207605	0	0.8357301
axo09144-f	ZNF268	0.001256	0.00042967	1	1.95410998	0.02586658	0	0.7129328
axo09177-f	CXCR4	2.78E-08	9.14E-08	1	5.34554854	0.07978712	0	1.26726069
axo09194-f	HMGA2	5.438E-06	0.00004756	1	4.3487959	0.24632537	0	1.30204622
axo09204-r	ARD1A	0.00183	0.00117179	1	1.69721696	0.76579106	0	0.96472523
axo09211-f	TRRAP	0.002988	0.00079761	1	4.33000893	0.59373691	0	1.18472728
axo09216-f	GSTP1	0.000551	0.00115593	-1	0.39512654	0.70769713	0	1.08252674
axo09230-f	HIST2H4A	0.000315	0.0011337	1	3.29192469	0.12672836	0	1.55040021
axo09233-r	GSTT1	0.005183	0.00219004	-1	0.50191408	0.28526311	0	1.20874255
axo09247-f	BFSP2	0.000108	6.38E-05	-1	0.50418333	0.59607116	0	1.05825698
axo09260-r	ZNF282	0.003755	0.00443193	1	1.62679937	0.01694021	0	0.68356953
axo09275-r	DHX16	0.000047	2.38E-05	-1	0.49634334	0.36503109	0	0.91466795
axo09285-r	TPST1	0.00000165	1.19E-06	1	2.58303645	0.58014408	0	0.94948712
axo09286-f	GUCY1B3	3.384E-06	5.77E-06	-1	0.34721728	0.1390354	0	0.82376724
axo09299-f	RAE1	0.003014	0.00185725	1	2.12741919	0.49651894	0	0.88117125
axo09309-f	HMGCR	0.001124	0.00042024	1	2.60247926	0.95670879	0	1.01028337
axo09316-f	PPM1D	0.005791	0.00346133	1	1.59703028	0.62848705	0	0.94067852
axo09360-r	BCAS1	0.000147	0.00015374	-1	0.23387156	0.77981704	0	0.93209463
axo09380-r	YARS	0.002578	0.00196386	1	1.64294403	0.79077454	0	0.96812148
axo09383-f	PDXK	0.002872	0.00116497	-1	0.43504976	0.54214826	0	1.12344228

axo09429-f	OASL	5.575E-06	3.84E-06	1	15.5892325	0.69264476	0	0.88553015
axo09431-f	AOC3	0.004415	0.00218786	-1	0.28849876	0.53307328	0	0.82250765
axo09471-f	EIF3J	0.002582	0.00052179	1	1.6323646	0.83559258	0	1.02087295
axo09477-r	VAMP4	0.005864	0.00861305	-1	0.68296616	0.86459021	0	1.02062107
axo09490-f	B3GALT2	0.00011	0.00023237	-1	0.45993185	0.2982037	0	0.85939761
axo09494-f	ABCC3	0.000027	2.18E-05	1	6.62000512	0.89333314	0	1.03512739
axo09505-r	EED	0.00004376	0.00076093	1	1.61803708	0.07701564	0	0.82031808
axo09529-f	FADD	4.016E-06	1.64E-05	1	4.79146114	0.41060863	0	1.18868792
axo09533-r	NAPA	0.001024	0.20587398	0	0.79922263	0.00571592	-1	0.5588806
axo09535-f	MPDZ	0.00001621	2.54E-05	-1	0.24038565	0.18013104	0	0.75735626
axo09537-f	RIOK3	0.00002934	1.84E-05	1	2.87786783	0.98358528	0	0.99710223
axo09539-f	MATN4	0.004299	0.00508339	-1	0.23721163	0.06254969	0	2.3214325
axo09555-f	IL1RL2	0.00001646	2.05E-05	1	2.78657439	0.41462616	0	1.12190538
axo09563-f	FGF18	0.000375	0.000114	1	4.10834784	0.88348562	0	0.96611734
axo09573-f	GMPS	0.002838	0.00060355	1	1.98211362	0.4241386	0	0.89124666
axo09575-f	TMEM11	0.000181	0.00033103	1	1.50350222	0.08175761	0	0.86321031
axo09606-f	SQSTM1	0.00002453	5.40E-05	1	4.20893433	0.89308554	0	0.97110374
axo09621-r	EIF2B5	0.001004	0.0015592	1	2.16566536	0.99491303	0	0.9988325
axo09622-f	EIF2S2	0.000118	5.62E-05	1	1.70405276	0.71294618	0	0.97043938
axo09646-f	MBD2	0.00006079	3.63E-05	1	2.41334304	0.99579005	0	0.99932787
axo09654-f	NPY	0.0004	0.00170864	1	2.07603102	0.58191848	0	0.90713887
axo09688-f	NPR3	0.000319	0.00144168	1	1.81165595	0.43800347	0	1.11601365
axo09732-r	BNIP3	0.001545	0.00234576	-1	0.58111812	0.66929507	0	0.9429196
axo09744-r	AP2S1	0.005143	0.00817568	-1	0.71513458	0.67823108	0	0.95751257
axo09745-f	PLK3	0.003519	0.00159258	1	3.0197965	0.9481183	0	1.01725521
axo09747-f	CRY1	0.003786	0.00093762	1	3.71148362	0.28266151	0	0.72617391
axo09755-f	DDIT3	4.60E-10	4.40E-10	1	6.87071385	0.44515753	0	1.0655291
axo09766-r	EIF4EBP2	0.000242	0.00002971	1	2.28121696	0.36223286	0	0.89719097
axo09779-r	FDX1	0.00001928	1.18E-05	1	3.12807116	0.60922306	0	1.07721782
axo09798-r	IDH3G	0.001335	0.42534028	0	0.90094295	0.00685655	-1	0.65226494

axo09810-f	RAB1A	0.000122	0.00010999	1	1.7300859	0.59976848	0	0.95314438
axo09816-f	PLAT	0.004982	0.00139119	1	1.67950024	0.66149824	0	0.94819126
axo09826-r	SNRPD3	0.002649	0.0020969	1	1.82430464	0.47925363	0	1.11280952
axo09827-f	TANK	0.000478	0.00032825	1	2.76129431	0.65435234	0	1.09106496
axo09862-f	STK17B	0.005279	0.00139803	1	2.56214655	0.77111876	0	1.06609475
axo09921-f	CARTPT	0.000228	0.00166017	1	2.2138774	0.51799023	0	1.1324042
axo09935-f	ACTL6A	0.00003858	4.36E-05	1	2.43856033	0.60600164	0	0.93384132
axo10016-f	CREB1	0.000132	0.00007422	1	2.676667	0.99846866	0	0.99970199
axo10023-f	PPIC	0.005607	0.00645424	-1	0.6271214	0.96223253	0	1.0066043
axo10042-f	DDX11	0.000514	0.00014786	-1	0.34031925	0.7065032	0	1.07254834
axo10052-f	DUSP5	0.001326	0.0012138	1	6.1123098	0.71413592	0	1.16426059
axo10086-f	EPS8	0.001767	0.00467707	-1	0.61201843	0.20413041	0	0.83229796
axo10093-r	ACSL4	0.000563	0.00021889	1	4.28291684	0.849637	0	0.95132377
axo10150-r	HSF2	0.00015	9.61E-05	-1	0.60057015	0.0949668	0	0.861097
axo10191-f	NEB	0.001412	0.00155717	-1	0.4482947	0.19087546	0	1.29757806
axo10219-r	NFKBIE	0.00012	0.00031003	1	2.42011511	0.53367177	0	1.11088335
axo10221-f	YBX1	0.000323	0.00060846	1	1.6878293	0.31594318	0	0.8942741
axo10254-r	TRA2B	0.001061	0.00059077	1	2.13911395	0.59332562	0	0.91902329
axo10257-r	SNRPA	0.000189	6.22E-05	1	1.68543774	0.41114851	0	1.06975222
axo10281-f	VIPR1	0.00221	0.00581708	1	1.86002541	0.06961098	0	0.69742146
axo10298-f	CDK2AP1	0.001325	0.00131253	1	2.55155251	0.73050289	0	1.07768932
axo10303-f	COIL	0.000122	7.19E-05	1	2.90392461	0.72551637	0	1.06077009
axo10306-f	C21orf33	0.00007424	0.00168566	-1	0.74840616	0.03615226	0	0.84843929
axo10311-f	SDPR	0.00003772	0.00002605	-1	0.35588261	0.98223911	0	0.99680739
axo10352-f	SLC16A6	0.001652	0.00092097	1	2.16521507	0.39875509	0	1.15709795
axo10353-f	SLC16A6	0.002603	0.00048264	1	2.98439811	0.95554857	0	0.9878382
axo10354-f	SLC16A6	0.004388	0.00149064	1	3.46557322	0.43383366	0	1.26223803
axo10356-f	SLC16A6	0.003982	0.00130728	1	3.28640664	0.4713291	0	1.22231659
axo10366-r	RRP9	0.002979	0.0005889	1	2.146714	0.84055643	0	1.03224875
axo10383-f	SFRS2IP	0.00183	0.00065402	1	1.79251559	0.23416029	0	0.85988562

axo10389-r	ZW10	0.004914	0.00198426	-1	0.60906608	0.19350378	0	0.84713101
axo10393-f	ZBED1	0.003544	0.00753149	-1	0.48603328	0.27833562	0	0.78106436
axo10395-f	ETF1	0.00005433	2.50E-05	1	2.4917051	0.5907926	0	0.93383744
axo10400-r	SLC33A1	0.00008332	0.00004629	1	2.30834789	0.81437718	0	1.02972326
axo10405-r	MTA2	0.00002948	0.00033239	1	1.64695789	0.396431	0	1.08584211
axo10407-r	NOLC1	0.00000261	9.09E-07	1	4.64511184	0.60960499	0	0.92768055
axo10415-r	TBRG4	0.00061	0.00028141	1	3.77967014	0.56432693	0	0.86550596
axo10418-f	CRLF1	5.17E-07	5.81E-07	1	19.4721102	0.76973964	0	0.92417844
axo10470-r	SNRNP40	0.000332	0.00072256	1	2.08633332	0.88819065	0	1.022198
axo10481-r	CYP7B1	0.000115	0.00079399	-1	0.42857373	0.68353167	0	0.92810611
axo10495-f	MED26	8.746E-06	8.62E-06	1	2.48265765	0.99078456	0	1.00128476
axo10500-r	EIF2AK3	0.000288	0.00014013	1	3.88634506	0.43091821	0	0.83060761
axo10537-r	ITM2A	0.0000708	0.00012636	-1	0.62743446	0.07726408	0	0.85976841
axo10569-r	WTAP	0.000117	8.29E-05	1	1.74700774	0.37021511	0	1.08509899
axo10587-f	C21orf2	0.000872	0.00377044	-1	0.60374253	0.26032699	0	0.85226067
axo10688-r	PDE1C	0.000848	0.00321893	1	2.02510166	0.42657334	0	1.16334688
axo10691-r	PFN1	0.001498	0.0018565	1	1.33489537	0.6843164	0	0.97169582
axo10713-f	RELN	0.004836	0.00199086	-1	0.1908495	0.93257993	0	1.03507837
axo10725-f	RAPSN	0.003888	0.00442652	1	3.8375645	0.65606742	0	1.18316067
axo10743-f	XBP1	0.002513	0.00058264	1	1.82845273	0.99171025	0	0.99870927
axo10748-f	TRIM25	0.00006147	0.00004315	1	3.10160579	0.699491	0	0.93728086
axo10770-f	LGI1	0.000224	0.00005594	-1	0.28256474	0.74654798	0	1.06439994
axo10775-r	ISG15	0.001072	0.00042831	1	3.4888865	0.76226246	0	0.92787991
axo10787-f	COL7A1	0.00069	0.00224157	1	2.53138393	0.35869757	0	1.24411053
axo10812-f	NUP153	0.004523	0.00208956	1	1.91861113	0.51728587	0	0.89965598
axo10817-f	CLEC2B	0.0000398	4.87E-05	1	2.93503356	0.86437819	0	0.97280013
axo10842-f	ACTC1	0.001492	0.00160559	-1	0.22305025	0.57270896	0	1.22539539
axo10855-r	RND3	0.0001	7.74E-05	1	2.67140738	0.80223669	0	1.03985569
axo10859-r	ATP5G2	0.003183	0.00388795	-1	0.76763018	0.67989346	0	1.03042298
axo10896-f	ELK3	7.772E-06	1.2498E-05	1	3.18559604	0.54100693	0	0.91277425

axo10897-r	ELK3	0.002903	0.00552527	1	2.34801181	0.433122	0	0.82099841
axo10906-f	ETS1	7.25E-07	5.79E-07	1	4.09055425	0.78479563	0	1.03549673
axo10910-f	ETS2	0.000996	0.00018608	1	2.3848359	0.50529731	0	1.10928674
axo10912-f	F2RL1	5.30E-08	5.94E-08	1	13.5202195	0.48449603	0	1.13965014
axo10913-f	EWSR1	0.000124	0.00023389	1	2.00788039	0.65684114	0	0.94481471
axo10951-f	GPD1	0.000604	0.00039243	-1	0.56832943	0.04679891	0	1.27685851
axo10968-f	HIST1H1B	3.852E-06	2.89E-06	1	9.15672693	0.24788292	0	1.33177466
axo10972-r	H3F3B	0.004264	0.00692418	1	1.67526965	0.44012425	0	1.1298203
axo10994-r	NCKAP1L	0.001697	0.00112787	-1	0.29391706	0.63370056	0	1.14193985
axo11000-f	HSPA5	0.00001969	7.54E-06	1	4.02705916	0.31816165	0	0.8420435
axo11025-f	NFIL3	7.18E-09	3.49E-09	1	9.6075972	0.9605821	0	0.99413017
axo11030-f	PHF2	0.000462	0.0001108	-1	0.41042988	0.49358926	0	1.10775512
axo11031-r	PHF2	0.000165	4.31E-05	-1	0.41560848	0.72397997	0	0.95510505
axo11064-f	STAT2	0.003891	0.00317216	-1	0.61814677	0.72460268	0	0.95601224
axo11077-f	YES1	0.000728	0.00040624	1	1.87365933	0.09466344	0	0.80088851
axo11082-f	MALL	0.001295	0.77556488	0	1.06073014	0.00102242	1	2.52342289
axo11085-r	MLF2	0.005998	0.00094638	-1	0.56192455	0.3807146	0	1.12042028
axo11131-f	ABCA1	0.005455	0.0037629	1	2.41490315	0.75518744	0	0.92778172
axo11164-f	HSPG2	0.003513	0.00152065	-1	0.42562162	0.76638517	0	1.06193711
axo11204-r	LMAN1	0.003196	0.01028733	1	1.56666675	0.22961659	0	0.83383471
axo11219-r	NIT1	0.001458	0.00180179	-1	0.47565496	0.71919284	0	1.06711501
axo11230-r	REST	0.004267	0.00120685	1	2.32235255	0.64529835	0	0.91479074
axo11237-f	ACSM3	0.00001219	2.55E-06	-1	0.38731251	0.63991444	0	1.04870265
axo11254-r	TERF2	0.000687	0.00205944	1	1.60113246	0.32677598	0	1.12425009
axo11258-f	TFCP2	0.001804	0.01033682	-1	0.69069356	0.80898508	0	0.97135193
axo11260-r	NR2F1	0.001429	0.00065597	-1	0.38179232	0.81741174	0	1.04771888
axo11262-f	KLF10	9.28E-12	6.40E-12	1	14.6846638	0.21763033	0	0.909486
axo11295-f	DNM1L	0.001627	0.04861522	0	1.21571411	0.00221456	-1	0.70039046
axo11299-f	TSSC4	0.002588	0.00047727	1	2.06234973	0.64250535	0	0.93450373
axo11317-r	TSPAN3	0.002477	0.01191225	1	1.5046963	0.17142389	0	1.2163578

axo11341-f	ARIH1	0.001947	0.00247487	1	1.74317692	0.76978925	0	1.04233811
axo11351-f	CLEC3A	0.000141	0.00015759	-1	0.39155331	0.73374588	0	1.05700949
axo11353-f	EBI3	0.00002276	7.65E-05	1	5.01774446	0.92770207	0	1.02344838
axo11393-f	THOC4	0.00092	0.00026488	1	1.87034792	0.68113844	0	1.04905962
axo11401-f	MPHOSPH10	0.000792	0.00013688	1	2.60493733	0.74340952	0	0.94790168
axo11408-r	NUTF2	0.000166	0.00046351	1	1.78140926	0.72549365	0	1.04144742
axo11410-f	EIF1	0.00003511	2.39E-05	1	1.56213593	0.66440946	0	1.02712849
axo11416-r	DDX39	3.16E-07	2.54E-07	1	3.49336406	0.7392231	0	1.03516925
axo11437-f	GRB10	0.00487	0.00213036	1	2.28697564	0.91539963	0	1.02209054
axo11464-r	SDCCAG10	0.000661	0.00065399	-1	0.65642268	0.87937872	0	0.98670998
axo11466-r	SMNDC1	0.005753	0.00167718	1	1.40959756	0.85400507	0	0.98495747
axo11488-f	SMAD3	0.003014	0.00426005	1	1.74195658	0.87630603	0	1.02428486
axo11493-r	MANBA	0.000109	0.00014117	-1	0.49373312	0.73420931	0	0.95975362
axo11515-f	MCM7	0.000427	0.00974627	1	1.86238456	0.13081741	0	0.72566789
axo11534-r	MLL	0.002225	0.00752048	1	1.53572795	0.12338319	0	1.24074875
axo11578-r	HNRNPM	0.000523	0.00010318	1	2.65088913	0.29613155	0	0.84172255
axo11600-f	VPS72	0.000716	0.00015529	-1	0.60034318	0.37863112	0	0.92373034
axo11609-f	TUBA1A	0.00239	0.00079971	-1	0.45771146	0.41578103	0	0.86992564
axo11615-f	MANF	0.000015	1.36E-05	1	2.44518451	0.3669784	0	1.11204615
axo11659-f	MAP3K15	0.002061	0.00142173	-1	0.63949102	0.89015232	0	0.98565432
axo11716-r	NDC80	0.001611	0.00603917	-1	0.59664774	0.00751199	-1	0.60836891
axo11722-f	CROP	0.00002242	2.38E-05	1	1.88678234	0.30346542	0	1.09686806
axo11723-r	CROP	0.000573	0.00339639	-1	0.59921584	0.12509069	0	0.79939272
axo11725-f	CROP	0.003389	0.00074184	1	1.5312484	0.8036875	0	1.02285596
axo11726-r	SPON1	0.000217	0.00043603	-1	0.29367798	0.61758877	0	1.12968431
axo11749-r	PIM3	0.00000498	3.04E-06	1	3.51927682	0.81635105	0	1.03230313
axo11770-f	2-Sep	0.002527	0.00739746	-1	0.67941297	0.46814515	0	0.91687587
axo11795-r	NTS	0.00002451	4.41E-05	1	11.0141659	0.97968686	0	0.99099728
axo11800-f	OAS3	0.000784	0.00100916	1	2.20333928	0.42388128	0	0.86676392
axo11802-r	OAS3	1.54E-07	6.78E-08	1	14.4886238	0.08598083	0	0.69943958

axo11819-f	PCSK5	0.000151	0.00067269	1	2.23387584	0.17152841	0	0.78439179
axo11847-f	POLE	3.021E-06	1.82E-06	-1	0.42290768	0.04370806	0	0.81947111
axo11884-r	PSME1	0.000201	0.00049661	1	1.5756022	0.97881664	0	0.99757001
axo11904-f	SFRS6	0.001018	0.00026555	1	1.54337289	0.49096304	0	0.94542833
axo11919-f	TFDP2	0.002488	0.00160519	-1	0.43017761	0.58157159	0	0.89439643
axo11930-f	TSG101	0.001045	0.00050244	1	2.25563974	0.40322859	0	0.86950727
axo11940-f	ANP32A	0.001945	0.00118239	-1	0.56454233	0.85465352	0	0.97641746
axo11946-f	SRPX	0.003501	0.00127339	-1	0.36862752	0.99971585	0	1.00008179
axo11972-r	RAN	0.00078	0.00018046	1	1.64403764	0.842396	0	0.98270252
axo11973-r	TIMM23	0.001826	0.00130748	1	1.74011904	0.71547772	0	0.95426465
axo11993-f	COG5	0.000534	0.00114666	-1	0.68390223	0.38541009	0	0.92657393
axo12003-f	SEC23A	0.000245	0.00738685	-1	0.74096159	0.25867295	0	0.89860853
axo12010-f	SYNCRIP	0.000207	0.0001107	1	2.90667748	0.33663545	0	0.84014874
axo12031-f	IPO7	0.00124	0.00021952	1	1.96143066	0.54464224	0	0.9281801
axo12032-f	NOP56	0.000714	0.00017965	1	1.81255319	0.20097214	0	1.15033708
axo12033-r	ATG7	0.0000247	0.00120232	-1	0.65629302	0.11393398	0	0.84996166
axo12040-f	ANP32B	0.000477	0.00057432	1	1.83049437	0.80023044	0	1.03200741
axo12082-r	CCT2	0.000509	0.00016823	1	1.7065902	0.6719111	0	0.96102997
axo12097-f	SMC2	0.0000193	0.46188922	0	0.93735233	0.00018148	-1	0.61190319
axo12106-r	TBL3	0.000871	0.00017157	1	2.11962787	0.78743658	0	0.96506722
axo12114-r	RBCK1	0.00003022	4.39E-05	1	1.64449277	0.32741363	0	0.92871832
axo12126-r	TXNIP	2.15E-07	1.45E-07	1	3.25463705	0.31831112	0	0.9100157
axo12147-f	PSMC4	0.002308	0.00133298	1	1.52787516	0.67205653	0	1.04265606
axo12153-f	RELB	2.109E-06	1.17E-06	1	4.7270059	0.32229092	0	0.8570435
axo12159-f	SLC2A1	0.001157	0.00044201	1	3.35552848	0.68587173	0	1.10247555
axo12168-f	ASTL	0.000605	0.00393027	1	1.84381341	0.42972319	0	0.87410369
axo12174-f	TFPI2	0.000344	0.00015016	1	7.00222458	0.21461785	0	1.54276608
axo12175-f	TFPI2	0.002635	0.00086331	1	6.4757876	0.40205661	0	1.41493783
axo12191-f	TUSC4	0.004329	0.00583669	-1	0.70359396	0.56170066	0	0.94151936
axo12210-f	CGREF1	0.001172	0.00127205	-1	0.37794329	0.73076465	0	1.08034992

axo12214-r	CSPG5	0.001183	0.00111578	-1	0.45817511	0.59247597	0	0.90934228
axo12238-r	SULT1C4	0.003382	0.00332774	-1	0.65595148	0.58408134	0	0.93985103
axo12253-f	EPSTI1	0.002736	0.00081517	1	5.77219618	0.61358367	0	0.82499084
axo12269-f	PLK2	0.00002151	9.47E-06	1	3.8400737	0.44189375	0	1.13869129
axo12270-f	PLK2	0.000825	0.000346	1	5.46134766	0.97448646	0	1.01047806
axo12279-r	VAMP5	0.000827	0.00030354	1	2.47762298	0.70445564	0	1.06735074
axo12296-r	UTP14A	0.00192	0.00041501	1	2.4586397	0.75877289	0	0.94698031
axo12297-r	C7orf16	0.00005433	5.08E-05	1	3.31576095	0.62120421	0	0.91413174
axo12320-f	FGL2	0.002425	0.00136532	1	1.97695633	0.08349773	0	0.74265256
axo12324-f	LYVE1	0.00483	0.00653758	-1	0.47372509	0.42354739	0	1.19912606
axo12335-f	GADD45G	3.45E-07	2.08E-07	1	8.48471774	0.80801903	0	0.95871019
axo12343-f	SMPDL3A	0.000868	0.0028723	-1	0.41039982	0.57572623	0	0.87731996
axo12362-f	DIAPH2	0.000189	0.00359656	-1	0.59536102	0.92647405	0	1.01300667
axo12394-f	IFRD2	0.00001489	5.09E-06	1	3.84256767	0.96502939	0	1.00681876
axo12402-f	DDX18	0.00003656	7.20E-06	1	2.30194921	0.81701455	0	1.023407
axo12424-f	EHD1	0.001309	0.00050677	1	2.15246334	0.91406002	0	1.01686536
axo12429-f	UGT2A1	0.00114	0.00124259	-1	0.41077188	0.99606226	0	0.9989936
axo12435-r	SF3A3	0.004037	0.002269	1	1.53154278	0.88562151	0	0.984719
axo12439-f	BTG3	0.000316	0.0002449	1	14.7055958	0.48117865	0	1.42186583
axo12450-f	PNRC1	0.000138	0.00055815	1	3.11679514	0.52141778	0	1.16288089
axo12457-r	IFI44L	0.00007975	0.00037102	1	2.22751838	0.81919595	0	1.03615991
axo12458-f	IFI44L	5.58E-09	5.34E-09	1	6.15432088	0.80662833	0	1.02481725
axo12462-f	PKIA	0.000334	0.00015372	1	2.53936011	0.1970163	0	0.80476214
axo12463-f	EBNA1BP2	8.774E-06	4.67E-06	1	3.21646502	0.68629237	0	1.05551226
axo12477-r	CCNI	0.001854	0.00972053	-1	0.61575855	0.8037078	0	1.03947616
axo12495-f	SLC38A3	0.003341	0.00265965	-1	0.66527866	0.18943006	0	1.15502173
axo12518-f	RAB35	0.00004756	2.98E-05	1	1.96668604	0.49678601	0	1.06794203
axo12522-f	STON1	7.414E-06	3.77E-06	-1	0.17851786	0.17730359	0	0.76205481
axo12556-r	SFRS1	0.00005732	8.68E-05	1	2.38389716	0.98915675	0	1.00190033
axo12557-f	SFRS5	0.000501	0.00024442	1	1.86307618	0.97568998	0	1.00348334

axo12558-f	SFRS5	0.003899	0.00114673	1	2.57027311	0.37853398	0	0.82501428
axo12589-r	ZNF182	0.00031	8.05E-05	1	2.32007482	0.88577718	0	0.98091339
axo12594-f	ZNF22	0.000375	8.98E-05	1	1.908666	0.89100422	0	0.98578983
axo12605-f	ADAMTS1	0.000682	0.00018648	1	3.93752204	0.86467279	0	0.95945076
axo12625-f	DDX52	0.001738	0.00053587	1	1.61932324	0.73747209	0	1.03355106
axo12732-f	SMAD1	0.00182	0.00064391	1	2.16364429	0.72919438	0	0.94555547
axo12734-f	ZXDB	0.00003721	0.00001993	1	2.80005791	0.81583342	0	0.96816577
axo12781-r	DUSP10	6.89E-09	3.99E-09	1	10.8028245	0.82386299	0	0.97208452
axo12793-f	RNF139	0.002603	0.00128098	1	2.15345125	0.57553893	0	0.90505853
axo12821-r	VPS45	0.00004819	0.00066499	-1	0.69885513	0.04891157	0	0.84821094
axo12826-f	GPR182	0.002655	0.00365167	-1	0.35585005	0.26568162	0	1.37955216
axo12841-r	GABARAP	0.001515	0.00315824	-1	0.6774157	0.93145496	0	0.9911807
axo12874-r	SH3BGR	0.000238	7.47E-05	-1	0.4523271	0.87505399	0	0.98048695
axo12880-f	AP4E1	0.005782	0.00236656	1	2.746212	0.33739557	0	0.77807691
axo12885-f	MMRN1	8.37E-07	1.01E-05	-1	0.28637888	0.03968398	0	1.43268522
axo12888-r	CELA3B	0.00002386	8.32E-06	-1	0.48158098	0.32038596	0	0.91323496
axo12916-f	ATF5	0.001278	0.0019668	1	2.08843368	0.60831255	0	1.09781402
axo12924-r	HDHD1A	0.00152	0.00522384	-1	0.73946136	0.33687803	0	0.91820297
axo12925-f	ELL2	0.001676	0.00121719	1	3.72706591	0.59480246	0	1.17490898
axo12926-r	ELL2	0.000853	0.00081858	1	4.42388536	0.57446225	0	1.19943945
axo12948-f	ANGPTL2	0.00007561	7.39E-05	1	7.64238886	0.63224837	0	0.85695519
axo12963-f	CA14	0.001657	0.00197006	-1	0.34976157	0.71161759	0	1.1004648
axo12998-r	FBXO4	0.001887	0.00738917	1	1.77838868	0.2820775	0	1.21517015
axo13050-f	RRAS2	3.348E-06	3.80E-06	1	2.18357386	0.45347791	0	0.93594998
axo13056-f	HEY1	0.001385	0.00322003	-1	0.60603373	0.26221536	0	0.85726214
axo13089-r	G3BP2	0.003994	0.00089894	1	1.97379068	0.54976512	0	0.91405698
axo13104-f	LRRC29	0.001212	0.00333115	1	1.50713641	0.13217804	0	1.19134699
axo13133-f	MMD	0.00017	0.00003951	1	2.40337982	0.73785496	0	1.04394521
axo13156-f	TSPAN15	0.000436	0.0007423	1	2.17392274	0.42085789	0	1.14508551
axo13159-f	GTPBP4	0.005941	0.00085555	1	1.76700158	0.93270104	0	1.01050514

axo13198-f	IFIT5	0.00315	0.00149969	1	4.29041539	0.58679541	0	0.82855536
axo13199-f	IFIT5	0.000122	0.00007763	1	5.95729747	0.28560497	0	0.73226614
axo13200-f	IFIT5	0.000312	0.00021907	1	5.587255	0.37370845	0	0.75381618
axo13209-r	RSU1	0.00383	0.00110385	-1	0.60161259	0.28479111	0	0.88133149
axo13227-f	STEAP1	0.000035	3.96E-05	1	8.37771806	0.81917005	0	1.0737112
axo13231-r	TIMM10	0.000242	0.00017066	1	1.6314348	0.9523608	0	1.00513318
axo13245-f	TNPO3	0.000121	0.00037319	1	1.78280717	0.66786071	0	1.0495331
axo13260-r	ACAN	0.004265	0.0039658	-1	0.55152493	0.04442659	0	1.44274834
axo13263-f	FLRT2	0.005481	0.00494737	-1	0.65478492	0.56309378	0	0.93210497
axo13265-r	PDCD6	0.000074	7.0717E-05	1	2.09959345	0.58507322	0	1.06609795
axo13270-f	ATXN10	0.00005483	0.00165272	-1	0.64883115	0.10948365	0	0.83735889
axo13272-f	DNAJC15	0.0000106	7.38E-06	1	5.32605872	0.29692739	0	1.24072782
axo13286-r	HTRA2	0.000592	0.00030438	-1	0.57315821	0.12430774	0	0.84188806
axo13303-r	SAP30BP	0.001343	0.00093922	1	1.50637713	0.35913126	0	1.08830882
axo13307-r	BRD7	0.000186	0.00021976	1	2.0926103	0.51308973	0	0.91544006
axo13321-f	MAP1LC3C	0.005252	0.00763403	1	2.42588865	0.92118754	0	1.02727967
axo13324-f	RBM15B	0.000636	0.00031371	1	2.07182909	0.84429406	0	0.97322683
axo13329-f	MYLPF	0.00001572	6.65E-06	-1	0.12170286	0.09081651	0	1.57901112
axo13349-f	UBIAD1	8.515E-06	3.54E-06	1	5.59456386	0.63941163	0	0.91423545
axo13352-r	SNX10	1.83E-07	2.97E-07	1	4.46448367	0.52070026	0	1.08520931
axo13375-f	PCOLCE2	0.000952	0.0018755	1	2.4657582	0.85680597	0	1.04053464
axo13407-r	STOML2	0.003156	0.00388459	1	1.61129576	0.57908989	0	1.07566338
axo13449-f	OSTM1	0.000225	0.00013711	-1	0.49791947	0.31136154	0	0.88378376
axo13455-f	METTL7A	0.0000458	1.79E-05	-1	0.26065095	0.46447574	0	0.8757409
axo13460-r	SNX24	2.085E-06	0.00000319	-1	0.35958814	0.15271701	0	0.84466216
axo13479-f	MCTS1	0.004117	0.00261077	1	1.5844623	0.61971826	0	1.06074901
axo13510-r	CHD3	0.002427	0.00123371	-1	0.64528751	0.01009279	1	1.36438424
axo13527-f	HNRNPL	0.001025	0.00091044	1	1.66028011	0.74765577	0	0.96477204
axo13546-f	CNIH4	0.000296	0.00141199	1	1.86163469	0.50352655	0	1.10329353
axo13548-r	TMEM208	0.000826	0.00388757	1	1.50812917	0.41048304	0	1.09872017

axo13599-f	SLC25A13	0.002576	0.00984966	-1	0.66794289	0.03674164	0	0.73674554
axo13621-f	MGAT4B	0.00081	0.00038901	-1	0.52842489	0.3199511	0	0.88071735
axo13643-f	ETHE1	0.003988	0.00252844	1	2.28161599	0.7139755	0	1.08052491
axo13655-f	PDSS1	0.0026	0.00216639	1	1.75610933	0.92412444	0	0.98672069
axo13677-f	IL17RA	0.004472	0.00400507	1	2.47078818	0.56724322	0	0.86638069
axo13697-f	OPTC	0.000132	0.00015732	-1	0.3439058	0.61637059	0	1.09776684
axo13717-r	GNL3	4.523E-06	8.19E-07	1	2.48573012	0.98102117	0	0.9979663
axo13731-f	DNAJC2	0.00001419	5.62E-06	1	3.00500349	0.97031929	0	1.00477792
axo13732-f	ACAD8	0.000409	0.00024917	-1	0.53958457	0.89128193	0	0.98460866
axo13733-f	C1orf107	0.00000986	8.13E-05	1	2.40476736	0.1103498	0	1.27055427
axo13739-f	ANKRD1	2.05E-07	6.81E-08	1	29.5421994	0.51449751	0	0.85171146
axo13742-f	GHITM	0.000509	0.00015743	1	1.67827213	0.87807408	0	0.98632158
axo13780-f	SESN1	0.001724	0.00175421	-1	0.37888239	0.4419082	0	0.83279001
axo13782-r	PDCD4	0.000248	0.00072061	-1	0.49386147	0.91998613	0	0.98507354
axo13813-f	ZNF330	0.00293	0.0031287	1	1.51178188	0.89570155	0	0.98579302
axo13814-f	RAB30	0.00029	0.00696526	1	1.39323343	0.72614569	0	0.96542806
axo13836-r	CNOT2	0.00037	0.00068902	1	1.57145613	0.9922776	0	1.00092277
axo13848-f	SENP1	0.001011	0.00044601	1	1.84134037	0.18983359	0	0.84662442
axo13860-r	BRD1	7.359E-06	7.01E-06	1	2.89503943	0.76155593	0	1.03935632
axo13864-f	LMCD1	0.00569	0.00286474	-1	0.40295557	0.33467717	0	0.7913576
axo13874-f	EXT1	0.000609	0.00102764	1	1.67937374	0.32401739	0	0.88952394
axo13878-f	DNTTIP2	0.003587	0.00078149	1	1.93421701	0.57478925	0	0.92303953
axo13903-r	FAF2	0.000167	0.00007715	1	1.84870795	0.60352366	0	1.05226033
axo13968-r	LRRC14	0.003457	0.00137188	1	2.15776587	0.74736376	0	0.94384095
axo13990-f	HERPUD1	1.617E-06	1.20E-06	1	2.32625621	0.8751667	0	0.98707526
axo14052-r	KIAA0247	3.45E-07	1.63E-07	1	26.4823455	0.41356121	0	1.24018585
axo14063-f	MTSS1	0.00032	0.00177896	1	2.43684924	0.34867256	0	1.22957892
axo14150-f	ZC3H11A	0.000132	7.8163E-05	1	2.79761351	0.42685001	0	1.14106686
axo14186-r	SIAH1	0.0000166	1.65E-05	1	2.30927929	0.19816552	0	0.86207721
axo14232-f	NAV3	0.000258	0.01234601	1	1.60983928	0.4493131	0	1.13059456

axo14255-f	KIAA0831	0.000377	0.00119579	1	1.82691773	0.68915409	0	1.0567548
axo14285-f	DIS3	0.005714	0.00163699	-1	0.65421179	0.30723447	0	0.89905898
axo14298-r	MTMR15	0.000693	0.00028594	-1	0.42120895	0.32660377	0	0.84966812
axo14343-f	SPEN	0.002746	0.00099368	1	1.80148878	0.59963178	0	0.9332294
axo14355-f	SPEN	0.002305	0.00768135	1	1.85108824	0.97261399	0	1.00651593
axo14359-r	SPEN	0.00281	0.00200442	1	2.44103278	0.74411401	0	0.93058811
axo14364-r	SPEN	0.001523	0.00189605	1	2.00221097	0.93348493	0	1.01425237
axo14410-f	PDXDC1	0.002837	0.00177509	1	1.76055789	0.3511588	0	0.87766158
axo14425-f	PDS5B	0.003419	0.00211035	1	1.70577339	0.67177054	0	0.94513086
axo14439-f	SDC1	1.803E-06	1.34E-06	1	17.6784433	0.75468607	0	0.91463836
axo14487-f	PPRC1	6.897E-06	5.21E-06	1	5.94688015	0.2914145	0	0.79954622
axo14488-f	PPRC1	0.00002876	1.69E-05	1	7.46844729	0.45851184	0	0.81879375
axo14498-r	PEG10	0.001663	0.00102446	-1	0.39601372	0.23791737	0	0.77641667
axo14538-f	SIPA1L3	0.000542	0.0003052	1	4.28431038	0.90840406	0	0.96887805
axo14607-f	F5	0.005121	0.00700548	-1	0.3733595	0.80027908	0	0.9273201
axo14617-f	WDR43	2.949E-06	8.43E-07	1	3.33836878	0.98503327	0	0.99787038
axo14628-r	ZNF182	0.001949	0.000327	1	2.75259814	0.30847551	0	0.81708685
axo14629-f	ZNF182	0.004345	0.00646752	1	1.50661034	0.86770951	0	0.97984582
axo14632-r	ZNF182	0.005352	0.00146043	1	2.27717947	0.13414373	0	0.73523516
axo14640-r	STAB1	0.000143	0.00045284	-1	0.46676491	0.38205026	0	1.14486593
axo14670-f	PMPCA	0.003828	0.00181186	1	1.71484875	0.63087087	0	0.9387236
axo14672-f	ACSBG1	0.00006641	0.00110442	-1	0.33081021	0.31393607	0	0.77258701
axo14692-f	BAT2D1	0.001001	0.00191254	1	1.60593927	0.68835261	0	0.95441547
axo14749-f	OTUD3	0.000104	0.00006947	1	2.07664172	0.47040534	0	1.08727886
axo14878-f	IFRD1	2.298E-06	1.16E-06	1	3.50620091	0.36492954	0	0.89280685
axo14900-r	FEM1B	0.000119	0.00008158	1	2.02811482	0.96712688	0	0.99536024
axo14984-f	TRIM13	0.00531	0.00425177	1	1.67747624	0.15982841	0	0.80831508
axo14991-f	SUMF2	0.002481	0.00345198	1	1.70548019	0.44448016	0	0.89476579
axo15044-f	ZNF521	0.001492	0.00978436	-1	0.56396479	0.47020777	0	0.87400272
axo15061-r	SAMHD1	0.001855	0.00340973	1	2.52074352	0.68546834	0	0.90423918

axo15086-r	TIPARP	0.00000116	6.03E-07	1	15.9163615	0.66514957	0	0.89636818
axo15090-f	C20orf4	0.002366	0.00304378	-1	0.61906494	0.44638948	0	0.90713489
axo15158-f	HERC4	4.296E-06	2.70E-06	1	27.4940653	0.79841217	0	0.91311301
axo15161-f	TOR1AIP1	0.004898	0.00807966	1	1.38382131	0.36827881	0	0.91156578
axo15163-f	C1orf77	0.000148	5.05E-05	1	2.01856027	0.60019581	0	0.9457189
axo15207-r	RSL1D1	0.000726	0.00026347	1	2.27780672	0.63087386	0	1.07648832
axo15214-f	GTPBP5	0.000623	0.00323945	-1	0.65098761	0.01825884	0	0.73055848
axo15225-r	FAM184B	0.000868	0.00059935	-1	0.54658433	0.94268102	0	0.99105727
axo15231-r	CPAMD8	0.00007953	0.00034788	-1	0.50316855	0.59708741	0	0.93214545
axo15258-f	MMADHC	0.004517	0.00292561	1	1.76035289	0.65392963	0	0.93559821
axo15263-f	PPPDE2	0.004517	0.00953747	1	1.53720581	0.9732919	0	1.00461125
axo15267-r	RRM2B	0.00345	0.00052944	1	1.91516762	0.31024394	0	0.87127839
axo15281-r	WARS2	0.001096	0.000291	1	1.87398101	0.88198029	0	1.01765884
axo15297-f	AZIN1	0.001556	0.00037618	1	1.68723002	0.31039286	0	0.8995321
axo15302-f	PCF11	0.00001288	6.50E-06	1	2.97501031	0.19852306	0	0.84073575
axo15307-f	PRLH	0.000205	0.00889787	1	1.95391946	0.69328577	0	1.08730373
axo15352-r	TXNDC12	0.001172	0.00156434	-1	0.6278405	0.74609576	0	0.96478095
axo15365-f	NOP58	5.066E-06	2.90E-06	1	3.06214656	0.44738627	0	1.09779855
axo15369-r	TRMT6	0.000344	0.00013478	1	2.99122479	0.98783803	0	0.99716628
axo15375-r	C7orf20	0.005185	0.00292638	1	1.55970876	0.8565356	0	1.02122918
axo15434-f	FBN1	0.000937	0.00078066	-1	0.34725531	0.3315303	0	0.79773768
axo15443-r	UBE2J1	0.000119	3.62E-05	1	1.9162733	0.37702497	0	0.91867088
axo15449-f	METTL9	0.002406	0.00155655	1	1.37778808	0.61470877	0	0.96223199
axo15473-r	MRPL11	0.000667	0.00029551	-1	0.70333472	0.4456412	0	0.95004011
axo15474-f	RRP15	0.001724	0.00110284	1	1.90447674	0.6467264	0	1.06920365
axo15500-r	PTRH2	0.00009823	1.26E-05	1	2.3218273	0.32787257	0	1.11405387
axo15507-f	PALLD	2.296E-06	1.01E-06	1	4.22873162	0.41725367	0	1.12129043
axo15509-f	RBM7	0.000574	0.00040492	1	1.85938647	0.59353166	0	0.9366985
axo15521-f	SAR1B	0.004012	0.00200941	1	2.48821991	0.50567259	0	1.16328156
axo15537-f	NUB1	0.000492	0.00024065	-1	0.47598049	0.8410984	0	1.02764407

axo15545-f	TMEM66	0.00002031	5.42E-05	1	2.00375298	0.85561932	0	1.01939958
axo15546-f	TMEM66	0.000795	0.00070933	1	2.06210053	0.30725274	0	0.85142577
axo15555-f	PGCP	0.002255	0.00130558	-1	0.53176707	0.42929425	0	1.12438361
axo15558-f	CHCHD2	0.000843	0.00036249	1	1.51106599	0.88332208	0	0.98835872
axo15587-f	CERCAM	0.000314	0.00032217	-1	0.49820705	0.25225516	0	0.8546054
axo15588-f	CERCAM	0.000723	0.00033158	-1	0.42660605	0.63842203	0	0.92601534
axo15596-f	HN1	0.000292	0.00010661	1	1.78609234	0.95442719	0	1.00548391
axo15619-f	DDX41	0.000344	0.00046752	-1	0.60416102	0.03522878	0	0.78692897
axo15631-f	PADI3	0.000761	0.00095843	-1	0.49924585	0.85980979	0	0.97324325
axo15633-r	GPRC5B	0.00125	0.0003103	-1	0.52651395	0.64265873	0	0.9449326
axo15667-r	PLEKHO1	8.71E-08	4.75E-08	1	4.66466346	0.33769256	0	1.11041734
axo15685-r	HP1BP3	0.00008156	8.27E-05	-1	0.50596132	0.46334369	0	0.92218088
axo15687-f	TRAP1	0.002103	0.00039834	1	1.74118601	0.26357891	0	0.88213355
axo15688-f	PCYOX1	0.001051	0.00090811	-1	0.58432422	0.71703366	0	0.95807312
axo15702-r	DNAJB11	0.001061	0.00106985	1	2.11413231	0.52536361	0	0.89777137
axo15704-r	LCMT1	0.001757	0.00671548	-1	0.78305615	0.17180093	0	0.89995327
axo15749-f	ZDHHC2	0.00000209	3.78E-06	-1	0.31925188	0.03988176	0	1.34090869
axo15751-r	LIMA1	3.43E-07	4.56E-07	1	3.64595478	0.59888032	0	1.06230153
axo15793-f	PLEK2	0.002787	0.00125369	1	2.63356974	0.66140613	0	1.10282739
axo15853-f	NINJ2	0.002245	0.00182733	-1	0.28981269	0.886514	0	1.04388057
axo15858-f	SIRT6	0.000465	0.0002553	1	1.87890354	0.39129033	0	0.90330744
axo15887-f	GCNT4	1.188E-06	6.37E-07	1	3.90923957	0.1377721	0	1.21694643
axo15912-f	MEX3C	0.000908	0.00106185	1	1.6818414	0.01412427	0	0.71303609
axo15914-r	C21orf66	0.000549	0.00040482	1	1.68293136	0.79775935	0	0.97414493
axo15915-f	TNFRSF12A	0.00006258	2.17E-05	1	3.09583089	0.31697499	0	1.17120315
axo15936-f	ADD3	0.00005922	0.00022841	-1	0.57301521	0.43285656	0	0.922469
axo15947-f	F11R	0.001346	0.00145643	-1	0.59067345	0.96727788	0	0.99494292
axo15988-f	MVP	0.005297	0.00604527	-1	0.52985518	0.75379569	0	0.94287167
axo15998-f	SURF2	0.004183	0.00462738	1	2.06312896	0.39468283	0	1.19346479
axo16014-f	WBSCR22	0.00015	0.00014562	1	1.97317056	0.57829827	0	1.06753843

axo16047-f	PARP14	0.003825	0.00203826	1	4.67554541	0.28422152	0	0.6567182
axo16053-f	FAM20A	0.001372	0.00867677	1	1.69083905	0.84473962	0	1.03286617
axo16083-r	RNF111	0.005293	0.00594466	1	2.03304291	0.39242362	0	0.83383914
axo16103-r	C17orf59	0.005762	0.00090637	-1	0.6715779	0.40484741	0	1.07683584
axo16125-f	HAUS6	0.00003825	0.0012716	1	1.47187552	0.26355909	0	0.90228889
axo16178-f	VPS13C	0.00003042	0.0000263	-1	0.3031792	0.95054475	0	1.0103569
axo16202-f	ERMN	0.001535	0.0012321	-1	0.1908245	0.65571673	0	1.18534005
axo16226-f	RETSAT	0.00007623	8.27E-05	1	2.03032592	0.91599894	0	1.01201476
axo16228-r	NSUN2	0.000435	8.06E-05	1	2.46975626	0.90701208	0	1.01697602
axo16233-f	ALKBH5	0.00107	0.00079101	1	1.78682251	0.67745428	0	1.05351535
axo16245-f	ELOVL2	0.001434	0.00514197	-1	0.57328337	0.86665107	0	1.02714439
axo16246-f	PXK	0.004624	0.00143975	1	1.834434	0.38816446	0	1.13332136
axo16264-f	SEMA4C	0.001246	0.00032546	-1	0.52018607	0.19929141	0	1.18172718
axo16271-f	BTBD2	0.000525	0.00063357	-1	0.57862237	0.70513849	0	1.04425641
axo16274-f	CMTM6	0.000302	0.00262524	1	1.86638334	0.61046369	0	1.08565095
axo16286-r	CHCHD3	0.001085	0.00864566	-1	0.75385032	0.2693235	0	0.90373393
axo16293-f	ARHGAP28	4.559E-06	3.25E-06	1	4.76852804	0.69759405	0	0.93547997
axo16299-f	RHBDL2	0.00003541	0.00004743	1	13.5207162	0.67797081	0	1.17614913
axo16327-r	TMEM127	0.005037	0.00191194	1	1.73256122	0.19930231	0	1.19756365
axo16330-f	TMEM160	0.001099	0.01097023	1	1.36727585	0.30083858	0	0.89648872
axo16356-r	PINX1	0.00008563	0.00004371	-1	0.46373365	0.63922788	0	0.94782459
axo16377-f	DDX27	0.002138	0.00186751	1	1.67823142	0.62287566	0	1.06442134
axo16391-f	HERC6	4.73E-07	2.58E-07	1	49.2651314	0.81788073	0	0.92818224
axo16406-f	PRPF39	0.001248	0.00044451	1	2.18622972	0.76453987	0	1.04772662
axo16414-r	TTC38	0.00013	0.00031754	-1	0.5707705	0.49450052	0	1.07639829
axo16436-r	CDCA4	0.000424	0.00027757	1	2.48145048	0.69790888	0	1.06827025
axo16472-f	TAPBPL	0.001197	0.0067367	1	1.45834191	0.88407168	0	1.01665886
axo16503-f	WDR70	0.00126	0.0004536	-1	0.46380791	0.75711432	0	0.95368496
axo16532-f	PRPF38B	0.000566	0.00030127	1	2.63987387	0.84928617	0	0.96579618
axo16622-r	KLHL11	0.004557	0.00133777	1	1.89618667	0.61739549	0	1.07743711

axo16695-r	KLHDC8A	0.001443	0.00032407	-1	0.29076872	0.20284353	0	1.3671198
axo16702-f	LRRC1	1.556E-06	5.61E-07	1	2.69076053	0.6335183	0	1.04374913
axo16732-r	KIRREL	0.000396	0.00010566	-1	0.36531722	0.9150515	0	0.98244985
axo16742-f	NEIL3	7.056E-06	0.00215898	1	1.83632659	0.59714677	0	0.92256001
axo16769-f	ECHDC2	0.004336	0.00067147	-1	0.49270465	0.32362869	0	0.86015367
axo16808-f	PI4K2B	0.001288	0.00141416	1	2.49880675	0.66182062	0	0.9101959
axo16809-r	PI4K2B	0.00001256	4.99E-06	1	3.38852089	0.84136532	0	0.97229994
axo16831-f	RIOK2	0.000832	0.0002553	1	1.86969162	0.78966091	0	0.96962625
axo16843-r	LARP6	0.00575	0.00175498	1	3.24605475	0.64085745	0	1.14263662
axo16845-f	ABCF3	0.001802	0.00036378	1	2.42962165	0.46543729	0	0.88071919
axo16846-f	UFSP2	0.002219	0.01119428	1	1.36833328	0.83656125	0	0.97889589
axo16852-r	CXorf15	0.000013	1.21E-05	1	2.14769779	0.59023313	0	1.05411846
axo16863-f	C1orf103	0.000162	5.90E-05	1	2.2956388	0.28366502	0	0.86857761
axo16865-r	SYNJ2BP	0.000562	0.00037313	1	1.43141632	0.55882044	0	0.95980063
axo16896-f	CHST11	0.000045	0.00011268	1	2.34604876	0.27417353	0	1.17344102
axo16930-r	EAPP	0.001182	0.00301762	1	1.75155627	0.8148136	0	1.03510922
axo16935-f	PPP2R2D	0.003748	0.00126019	1	1.67316919	0.54033926	0	0.92946557
axo16938-r	ITFG2	0.004032	0.00094325	-1	0.54894011	0.71540082	0	0.95278878
axo16942-f	USE1	0.000129	0.00031474	1	1.9892717	0.73998817	0	0.95753093
axo16957-r	ASAP1	0.004327	0.00867146	1	1.46759341	0.00325989	-1	0.63547914
axo16974-r	LRRC59	0.000279	0.00088795	1	1.64923692	0.3714064	0	0.90500646
axo16990-r	CHST12	0.000379	0.00014203	1	1.84585471	0.31566446	0	1.11411668
axo16995-f	H2AFY2	0.00357	0.01048267	1	1.34721801	0.76281929	0	0.97109881
axo17005-f	BATF3	1.20E-08	9.23E-09	1	35.7120158	0.2197504	0	1.30495284
axo17012-r	GALK1	0.001696	0.0004674	-1	0.66750706	0.44513077	0	0.93926598
axo17017-f	MLL5	0.001819	0.00156635	-1	0.55962953	0.08404119	0	1.29444141
axo17020-r	MLL5	0.000229	0.00021231	-1	0.33424194	0.81030471	0	1.0485815
axo17028-f	CMAS	0.001136	0.00895034	1	1.39937052	0.87388107	0	0.98328281
axo17042-f	RBBP6	0.003157	0.00113627	1	2.43303206	0.45535527	0	0.85856861
axo17092-r	CYCS	0.000822	0.00015952	1	1.81865682	0.78665386	0	1.02855338

axo17093-f	ERRFI1	0.00002756	1.63E-05	1	7.54630982	0.85643272	0	0.9529713
axo17099-f	UBB	0.004599	0.00156566	1	2.19155264	0.96712341	0	0.99235622
axo17100-f	UBB	0.005819	0.00194249	1	2.08632802	0.58967859	0	0.90668689
axo17102-f	UBB	0.000267	8.4394E-05	1	2.87479617	0.39687166	0	0.864038
axo17103-f	UBB	0.002112	0.00048549	1	2.00068864	0.41868915	0	0.8916689
axo17179-r	CPVL	0.00001762	0.00042954	-1	0.67946933	0.41952027	0	0.93919958
axo17185-f	RHOF	0.000657	0.00051802	1	2.59730121	0.98429043	0	0.99619771
axo17191-f	ELP4	0.000161	0.00125404	-1	0.63273691	0.17564405	0	0.86110595
axo17192-f	MTRF1L	0.006014	0.00181106	1	1.80234892	0.9215946	0	0.98603425
axo17197-f	PUS7	0.004188	0.00204451	1	1.73976969	0.28476162	0	0.85996449
axo17206-f	ASNSD1	0.003975	0.00112477	1	1.45347978	0.80638692	0	1.02096518
axo17243-f	PAF1	0.000291	4.54E-05	1	1.67923716	0.76583341	0	1.0232526
axo17285-r	RBMXL1	0.005737	0.00134968	1	1.99926078	0.85840064	0	0.97169582
axo17286-r	IRGC	0.000015	4.65E-06	1	8.55561166	0.86258999	0	1.04328501
axo17290-f	IRGC	5.48E-07	1.97E-07	1	9.83033785	0.8774691	0	1.02881149
axo17302-f	PARD3	0.001087	0.00288316	1	1.96505905	0.62254616	0	0.9164887
axo17346-r	KCMF1	0.000121	3.5271E-05	1	1.91782533	0.16369302	0	0.87133013
axo17354-r	AGPAT4	0.000249	0.00028763	1	1.79661838	0.4076944	0	1.09697349
axo17363-r	SH3GLB2	0.000341	0.00105745	-1	0.61049865	0.2856822	0	0.88543195
axo17421-f	CTBP1	0.000153	4.77E-05	-1	0.50300082	0.62126518	0	0.95020893
axo17431-r	PRDM9	0.003238	0.00205471	1	1.82816084	0.94866061	0	0.99044218
axo17453-r	CABC1	0.000666	0.00072411	-1	0.52310266	0.68430495	0	0.94537001
axo17458-f	CCNL1	1.683E-06	1.03E-06	1	6.58459786	0.91016004	0	1.02071446
axo17461-f	CXCR7	0.000292	8.82E-05	1	4.23323037	0.29647936	0	1.28391231
axo17463-f	CIAPIN1	0.001214	0.00939164	-1	0.76974545	0.40136861	0	0.93117227
axo17516-f	UTP3	0.002446	0.00064087	1	1.67580777	0.53946852	0	0.93539222
axo17545-f	ATP13A1	0.005776	0.00173597	1	1.91943362	0.37082752	0	0.86619815
axo17557-f	SALL4	0.000642	0.00013222	1	7.94749571	0.64735999	0	1.17539962
axo17579-f	RGMB	0.001999	0.00228039	1	2.18724324	0.57156078	0	1.11870527
axo17591-r	C20orf3	0.001068	0.00092089	-1	0.46323548	0.63566105	0	0.92261075

axo17624-f	GALNTL1	0.004939	0.00245279	1	1.95277762	0.44074283	0	0.87531953
axo17661-r	RIMKLB	0.002206	0.00850901	1	1.49655953	0.97776436	0	1.00352299
axo17748-f	SEMA6A	0.000417	0.00017962	-1	0.30910209	0.60615248	0	0.89813715
axo17755-f	ARRDC3	0.004716	0.0052073	1	2.83262836	0.56165313	0	1.19129854
axo17779-f	KIAA1430	0.001477	0.00287093	1	1.6580927	0.21059803	0	1.18760028
axo17796-f	KIAA1462	3.408E-06	4.67E-06	-1	0.36581843	0.72617877	0	1.04110819
axo17825-f	LRCH2	0.000112	5.6864E-05	1	2.3763725	0.74604324	0	0.95799011
axo17829-r	LRRN1	0.000608	0.00012236	1	10.7962122	0.44670199	0	0.7344813
axo17865-f	FXR1	0.005231	0.01029542	1	1.50020744	0.06438329	0	0.7656939
axo17885-f	NIN	0.0000101	1.18E-05	-1	0.46061937	0.46306269	0	0.92944947
axo17893-r	CACHD1	0.002525	0.0011432	-1	0.33563661	0.67075337	0	1.1114325
axo17897-f	LRRC4C	0.000494	0.00018068	1	4.32341203	0.94583819	0	1.01769764
axo17913-r	KIAA1618	0.00009178	0.00003931	1	10.3539547	0.87231649	0	0.94659071
axo17949-f	DHRS4	0.001595	0.00066448	1	11.2337835	0.39787948	0	0.64581999
axo17950-f	DHRS4	0.000367	0.00015187	-1	0.46827178	0.20709987	0	0.84170932
axo17955-f	FAM53A	0.002678	0.00158907	-1	0.67283594	0.78489621	0	0.97456028
axo17962-r	SNTB1	0.00002125	2.76E-05	-1	0.45594846	0.27546613	0	0.88359531
axo17968-f	ZNFX1	0.000361	0.00013918	1	2.92835677	0.38433974	0	0.84983991
axo17987-f	HIST1H2AG	8.759E-06	1.18E-05	1	4.58850231	0.7828804	0	0.94806201
axo18008-f	PLSCR1	0.003099	0.0004713	-1	0.44271372	0.46689909	0	0.88664388
axo18010-f	MRPS12	0.002198	0.0046825	1	1.35123006	0.51785309	0	1.05707177
axo18026-f	RNASEL	0.000855	0.00118353	1	1.93268475	0.04639171	0	0.71666328
axo18039-f	XRCC5	0.00001561	0.00019214	-1	0.5953397	0.30811641	0	0.90791386
axo18041-r	CCNO	0.0016	0.00091418	1	4.18896065	0.84008624	0	1.06554117
axo18073-r	RHOU	0.001329	0.00184489	1	2.20548406	0.74906553	0	1.06363513
axo18142-f	SCPEP1	0.005957	0.00372099	-1	0.63382304	0.53183886	0	0.92477908
axo18146-f	PDLIM2	0.000138	0.00010336	-1	0.34923079	0.35483743	0	1.17781477
axo18147-f	ZNF350	0.004593	0.00160624	-1	0.65486359	0.98179245	0	0.99770163
axo18196-f	SLTM	0.000551	0.00017168	1	2.23363481	0.59856581	0	0.92804286
axo18217-r	BCAN	0.001194	0.00162905	1	6.46910741	0.69328436	0	1.19300686

axo18223-r	GRIK2	0.00001321	6.62E-05	-1	0.40461172	0.7330996	0	0.95300344
axo18227-r	DCUN1D2	0.000502	0.00047132	1	1.76043708	0.28678009	0	0.88313935
axo18230-r	ZNF148	0.000437	0.00013037	1	3.08225666	0.46068469	0	0.8671419
axo18250-f	ITM2B	0.000863	0.00094865	-1	0.66052115	0.45792106	0	0.93342111
axo18253-r	CUZD1	2.48E-07	1.83E-06	1	8.84947989	0.9566669	0	1.01223692
axo18258-f	SDF2L1	0.000962	0.00144533	1	1.84609014	0.90048326	0	1.01814521
axo18300-f	CA7	0.00075	0.00745371	1	1.62796611	0.15307482	0	1.25208196
axo18303-r	CHP2	0.004678	0.00334673	-1	0.54974558	0.90635	0	1.018967
axo18314-f	CARS	0.001105	0.00058711	1	1.97258345	0.81746204	0	0.96813938
axo18324-r	LHPP	0.00033	0.00010764	-1	0.44603794	0.88100953	0	0.9802251
axo18347-f	RRAGC	0.001528	0.00212688	1	1.7798093	0.58141289	0	1.08288247
axo18356-f	IFIH1	0.004506	0.00169762	1	5.30547038	0.69617153	0	0.8545973
axo18371-f	NCAPG	0.00009	0.00141917	-1	0.58374425	0.07967327	0	0.78676935
axo18376-f	OSGEPL1	0.000146	0.00194525	-1	0.70292419	0.03020912	0	0.80801072
axo18412-f	DDX19B	0.000349	9.26E-05	1	2.0913618	0.28658601	0	0.87687514
axo18420-f	GZF1	3.30E-07	1.73E-07	1	3.67874158	0.82942934	0	0.97792676
axo18435-f	TTC31	0.001394	0.00099676	1	2.45969483	0.61043855	0	0.90253201
axo18456-r	VPS52	0.000291	3.74E-05	-1	0.56797552	0.86259049	0	0.98587798
axo18457-f	MMP16	0.000131	3.86E-05	1	3.19695255	0.13946264	0	0.76689307
axo18461-r	PNKD	0.000953	0.00080086	1	3.20068419	0.52471075	0	1.17476105
axo18501-f	SMYD3	0.00000102	2.68E-07	1	16.1802837	0.75835096	0	1.07410622
axo18502-r	SMYD3	0.000434	0.00015944	1	4.59384549	0.99046084	0	1.003169
axo18504-f	SMYD3	0.001692	0.000739	1	3.18286507	0.65699815	0	0.8958042
axo18508-f	MOSC1	0.004778	0.00458542	-1	0.53619673	0.18271394	0	0.78288411
axo18525-f	ZNF574	0.001134	0.00063255	1	1.79762239	0.19285104	0	1.1812017
axo18536-f	MTHFSD	0.000217	0.00045949	1	2.13335245	0.56934913	0	0.91693286
axo18545-f	OSBPL11	0.000566	0.00519211	-1	0.71457962	0.40136751	0	0.92085272
axo18582-f	CBFB	0.002124	0.00121865	1	3.90996493	0.85935405	0	0.94620673
axo18620-f	UPF3B	0.002282	0.01134943	1	1.35664069	0.58153333	0	0.94557732
axo18661-f	RASL11B	0.000395	0.00030577	1	5.4739465	0.71251028	0	1.1260657

axo18669-r	HOXB9	0.004942	0.00500813	-1	0.43529027	0.31628249	0	1.27657061
axo18688-r	PDCL3	0.000162	0.00006782	1	2.34128247	0.77136249	0	0.96208793
axo18697-f	NOC4L	0.001907	0.0019178	1	1.68222576	0.51454114	0	0.91955302
axo18706-r	KDELC1	0.00109	0.00075233	-1	0.61424197	0.62286368	0	0.9498158
axo18717-r	WDR77	0.002728	0.00713108	1	1.52285298	0.39203498	0	0.89467359
axo18729-f	DHX58	0.000722	0.00063652	1	1.47526419	0.57263396	0	0.95491507
axo18741-f	TNIP2	0.000407	0.00020977	1	4.04599349	0.44466898	0	1.21540938
axo18742-f	PLEKHF1	0.002195	0.00029721	-1	0.46241529	0.80988762	0	0.96563977
axo18752-r	CRELD2	0.0000974	8.11E-05	1	3.54873866	0.97905766	0	1.00531573
axo18766-r	NPPC	0.0000293	1.48E-05	1	11.9380917	0.35307204	0	1.35873741
axo18795-f	CDC73	0.00028	0.00010792	1	1.72092155	0.06792961	0	0.83585098
axo18806-r	MUL1	0.002435	0.00046492	1	1.8472174	0.52566725	0	0.92438302
axo18817-f	LASS4	0.00299	0.00118318	-1	0.6694318	0.9204175	0	1.00917334
axo18821-r	ACSS3	0.00005612	5.22E-05	-1	0.50303151	0.80890038	0	1.02546553
axo18823-f	MPZL1	0.000726	0.00126246	1	2.32498488	0.28129628	0	1.24052086
axo18867-f	SFRS14	0.00162	0.00046994	1	2.38023937	0.41535892	0	0.86605267
axo18877-f	PPP1R3B	0.002509	0.00121612	1	1.44579433	0.65563826	0	0.96292455
axo18901-r	C11orf61	0.001505	0.00086913	1	1.75406069	0.13980816	0	0.82556631
axo18940-f	ESCO2	0.000182	0.00553427	1	1.84061642	0.01240341	-1	0.59043658
axo18975-f	SNIP1	0.000072	3.02E-05	1	2.95587856	0.69148564	0	0.94071743
axo18984-r	C15orf29	0.002223	0.00291609	1	1.66818621	0.65584586	0	1.06167461
axo19026-r	PRR5	0.000094	3.88E-05	-1	0.39492912	0.25693889	0	0.85307786
axo19053-f	CCDC134	0.001151	0.0028415	-1	0.55656111	0.06350935	0	0.73306143
axo19084-r	MRM1	0.000622	0.00027279	1	2.23668951	0.59637592	0	0.92319992
axo19085-f	SPEF2	0.000723	0.00312301	-1	0.39810595	0.32899551	0	0.78396908
axo19101-r	DENND2D	3.63E-08	2.04E-08	1	9.62241485	0.47837219	0	0.9021313
axo19102-f	WDR76	9.35E-08	6.13E-07	1	4.52781601	0.66945649	0	1.06076837
axo19104-r	GPR177	0.005659	0.00554577	1	2.06907891	0.60110644	0	0.89483794
axo19125-r	C14orf28	0.002196	0.00764026	-1	0.64366994	0.04929113	0	0.7441309
axo19135-r	FBXL18	0.000211	0.00108037	-1	0.66676133	0.36472034	0	0.91907744

axo19186-f	BXDC5	0.000285	8.48E-05	1	1.97625759	0.61998907	0	1.05603428
axo19196-f	TOE1	0.000131	4.62E-05	1	1.80064777	0.82907291	0	1.01910709
axo19216-r	MOGAT2	0.003798	0.00277075	-1	0.50571039	0.910251	0	0.98028988
axo19219-r	C16orf59	0.00001565	0.00002616	-1	0.5765644	0.03075165	0	0.82846462
axo19220-f	ZXDC	0.001557	0.00096094	1	2.51482905	0.71280793	0	0.92748831
axo19228-r	MUS81	0.004391	0.00670861	-1	0.54347667	0.95726002	0	1.00985022
axo19229-f	MUS81	0.002972	0.00225566	-1	0.54278484	0.20143876	0	1.22693629
axo19242-f	FHOD3	0.000103	0.00017621	1	2.93764826	0.98432311	0	1.00372937
axo19256-r	COQ10B	0.000076	7.26E-05	1	2.73272446	0.82356525	0	1.03593609
axo19297-f	PLEKHO2	4.272E-06	6.12E-06	1	3.81465786	0.11875034	0	1.29986488
axo19313-f	TRAF3IP3	0.00545	0.002254	-1	0.27435004	0.5754174	0	1.20099821
axo19314-f	REEP4	0.00003447	9.40E-05	1	1.98207012	0.81160403	0	0.97382583
axo19330-f	ITIH5	0.000139	9.72E-05	-1	0.24977119	0.5549005	0	0.87387672
axo19345-r	ZFP2	0.005142	0.00865285	-1	0.57050773	0.36830081	0	0.85047319
axo19363-f	SLC25A21	0.003868	0.00307422	-1	0.61268184	0.11030233	0	1.24690049
axo19367-f	DUSP16	1.726E-06	1.16E-06	1	2.53921989	0.21864918	0	0.88995975
axo19400-f	ZBP1	8.177E-06	0.00001051	1	4.85783111	0.7531365	0	0.93957181
axo19414-r	HM13	0.000205	0.00310143	1	1.6808908	0.89774086	0	0.98255245
axo19422-f	STMN4	0.000009	8.94E-06	1	3.69811734	0.54985666	0	0.9076282
axo19431-r	FAM117A	0.003567	0.0008307	-1	0.60219265	0.64320464	0	0.9501468
axo19454-r	TRIM8	0.001402	0.00032606	1	6.20713131	0.5857085	0	0.82609981
axo19458-f	LBH	0.001408	0.00295681	-1	0.5480154	0.5543802	0	0.91037825
axo19472-f	KAZALD1	0.000825	0.01659932	0	2.45791382	0.00446957	1	3.13914862
axo19480-f	CCNL2	8.47E-08	2.34E-08	1	4.36697418	0.53907771	0	1.06048925
axo19483-f	TMEM49	0.000151	0.00016224	1	3.66842638	0.36458928	0	1.23310872
axo19509-f	MSH6	0.004134	0.00182038	-1	0.60528017	0.08030124	0	0.79327262
axo19512-f	HYI	0.003211	0.00307895	-1	0.37739111	0.72298146	0	1.09555102
axo19524-f	HNRNPA2B1	0.00172	0.00164283	-1	0.6188693	0.63906606	0	0.94733943
axo19585-f	RTP3	0.000071	5.19E-05	1	5.91635382	0.63962752	0	1.13474935
axo19587-f	RTP3	0.000822	0.00071565	1	2.51200267	0.7978119	0	0.9511352

axo19639-r	TEKT3	0.00001821	4.32E-06	-1	0.35553208	0.99228496	0	1.0011307
axo19651-f	ESYT3	0.005385	0.00093587	1	1.50278644	0.38122765	0	1.08343255
axo19688-f	FANCD2	0.000761	0.00011909	-1	0.49980676	0.92269482	0	1.01132428
axo19706-f	FAM172A	0.003576	0.00049186	1	2.29567381	0.82640566	0	1.03741275
axo19711-f	B3GNT5	2.718E-06	7.15E-07	1	6.20302096	0.46909467	0	1.13208975
axo19746-r	C16orf48	0.005122	0.00914683	-1	0.68267776	0.13272579	0	0.82409906
axo19769-r	ACAD11	0.000937	0.0001546	-1	0.52655957	0.84154102	0	1.02244058
axo19775-r	PHAX	0.004156	0.00389332	-1	0.66336785	0.3527552	0	0.89876053
axo19785-f	ARID5B	3.861E-06	1.08E-06	1	3.58285337	0.97338879	0	0.99588243
axo19787-f	ASCC2	0.004044	0.00275196	-1	0.55006499	0.82833101	0	1.03413701
axo19825-f	WDR24	1.151E-06	1.31E-07	1	3.16978942	0.65810758	0	1.04036276
axo19827-r	ZNRF1	8.035E-06	2.46E-05	1	2.74476633	0.66518878	0	1.06250552
axo19858-r	C9orf89	0.001461	0.00032385	1	1.76214584	0.90240353	0	1.01331309
axo19874-f	S100A13	0.002683	0.00302219	1	3.63303098	0.50500569	0	1.25666345
axo19886-f	PDCD2L	0.000266	0.00014017	1	2.46362214	0.92040252	0	1.0155071
axo19895-f	CCDC77	0.001081	0.00117274	-1	0.38069732	0.48598206	0	0.85642392
axo19896-f	C3orf26	2.382E-06	1.73E-06	1	3.10977816	0.32804278	0	1.12317962
axo19900-f	FBLIM1	0.001331	0.00029085	1	2.32620676	0.14955235	0	0.78551805
axo19903-f	LZIC	0.003523	0.00167702	-1	0.66380635	0.58904356	0	0.94792796
axo19909-f	ELOF1	0.00005986	1.85E-05	1	2.60425972	0.78744434	0	0.96606533
axo19918-r	MKI67IP	0.000186	4.03E-05	1	1.8326526	0.66487548	0	0.96211861
axo19929-f	C2orf40	0.005877	0.00251919	-1	0.23262929	0.64831083	0	1.18604137
axo19944-f	EFCAB7	0.002643	0.00071209	-1	0.48166556	0.5188654	0	0.90400396
axo19962-r	HEATR7A	0.000659	0.00196965	-1	0.59684063	0.13152417	0	0.81604465
axo19965-f	STK19	0.00275	0.00051884	1	1.78623719	0.55374935	0	0.9321166
axo19968-f	PHF6	2.696E-06	1.08E-06	1	3.79297711	0.30128085	0	1.14725678
axo19974-f	MRPS6	0.00011	5.22E-05	-1	0.3970924	0.84274788	0	1.02810195
axo19980-f	DOT1L	0.000848	0.00040774	1	3.7254172	0.34480272	0	1.28363556
axo20005-f	GNPTG	0.001035	0.00391514	-1	0.72119752	0.1269234	0	0.86464311
axo20015-f	CFC1	0.000232	0.00159482	1	2.08722433	0.58874584	0	1.10008678

axo20020-f	SMEK1	0.001791	0.00097875	1	1.52022093	0.29406202	0	1.10547418
axo20032-r	USP32	0.003366	0.00298272	1	2.08056544	0.20005843	0	1.29309984
axo20048-f	PTP4A3	0.000261	0.00008576	1	2.24129777	0.51937832	0	0.91904432
axo20078-f	PPAPDC3	0.003127	0.00229905	-1	0.5685263	0.95663647	0	0.99230647
axo20101-f	ZC3H10	0.004331	0.00922108	1	1.34516082	0.7612792	0	1.02909512
axo20104-f	ORAI1	0.000613	0.00556052	-1	0.70692742	0.18430484	0	0.869212
axo20107-f	AIFM2	0.000029	5.79E-05	1	5.01815141	0.92056971	0	1.0249212
axo20138-r	CIRH1A	0.000514	0.00030135	1	2.75414529	0.86013577	0	0.9668853
axo20140-r	CIRH1A	5.121E-06	3.87E-06	1	2.88572936	0.70864575	0	1.04537387
axo20142-r	PPP1R15B	0.000658	0.0001713	1	2.80533294	0.58269163	0	0.90476707
axo20148-f	LIN52	0.00499	0.00765011	-1	0.7077408	0.64500497	0	1.05039746
axo20155-r	RAB2B	0.00192	0.00725393	-1	0.67519931	0.158253	0	0.83710104
axo20195-r	ARHGAP19	0.00001382	7.31E-06	-1	0.35631209	0.96481704	0	1.00547532
axo20228-f	TNFRSF6B	1.765E-06	1.89E-06	1	10.8780219	0.34539609	0	1.26833572
axo20240-f	CASP2	0.004784	0.00067649	-1	0.47753907	0.35365682	0	0.86285039
axo20250-f	NLRP1	0.00008346	4.32E-05	-1	0.42656544	0.20263821	0	0.84580481
axo20251-f	NLRP1	0.0001	8.39E-05	-1	0.46420587	0.44671481	0	0.90940531
axo20256-f	CSRNP1	0.000136	0.00005505	1	3.70346833	0.68518932	0	1.08444509
axo20277-f	ALG2	0.000554	0.00023791	1	2.72449781	0.72506011	0	1.06676446
axo20287-r	PNPT1	0.001888	0.00156508	1	3.03422765	0.4165909	0	0.80435187
axo20317-r	BAT4	4.579E-06	6.75E-06	1	2.17132698	0.37332638	0	1.08742281
axo20319-r	PIGS	0.004831	0.00098482	-1	0.57904806	0.45158797	0	0.91157167
axo20490-f	CIRBP	0.000112	5.68E-05	1	1.73107312	0.99636911	0	1.0003813
axo20591-f	CD63	0.00002361	1.13E-05	1	2.0130272	0.58938324	0	0.95312235
axo20609-f	RBMX	0.006027	0.00167608	1	1.98344721	0.52064301	0	1.11249589
axo20620-f	KRT8	0.003156	0.00080265	-1	0.46812855	0.37264422	0	0.86167755
axo20630-f	NASP	0.001388	0.00064189	1	2.26351573	0.34879514	0	0.84909978
axo20672-f	SFRS3	0.001123	0.00071975	1	1.7876075	0.96560044	0	1.00532827
axo20695-f	VIM	0.00123	0.00157662	1	1.54124904	0.4396131	0	0.92248882
axo20700-f	HIST2H2AC	0.00322	0.01203878	1	2.11580011	0.55038501	0	0.85994522

axo20707-f	RRP1	0.00223	0.00141234	1	2.19085803	0.55381822	0	0.89626815
axo20776-f	HNRNPAB	0.001178	0.00018199	1	1.62316913	0.70565019	0	0.96810135
axo20785-f	TRA2B	0.001971	0.0013208	1	2.54326243	0.91054117	0	0.97601823
axo20829-f	ATP5G2	0.002419	0.00263406	-1	0.72297951	0.62990153	0	1.04122847
axo20887-f	HNRNPM	0.000493	9.55E-05	1	2.4128786	0.24075625	0	0.83990613
axo20945-f	TNNT3	0.000037	0.00001705	-1	0.28511352	0.80416707	0	1.04208623
axo20953-f	EBNA1BP2	0.00091	0.0004379	1	3.07454161	0.79892743	0	1.05842668
axo21092-f	GADD45B	2.381E-06	1.041E-06	1	13.0260341	0.78754399	0	1.06916165
axo21205-f	RBMXL2	0.000327	0.00005127	1	1.72829162	0.92085868	0	1.00823131
axo21228-f	NFKBIA	0.000019	1.94E-05	1	15.4201761	0.31689586	0	1.45898596
axo21334-f	HIATL1	0.003522	0.00074222	1	1.75932457	0.09756879	0	0.80670121
axo21367-f	EXOSC6	0.000548	0.00027477	1	1.61981195	0.36313112	0	0.91989005
axo21480-f	PRDX1	0.00008544	0.00014258	1	1.68754191	0.89196779	0	1.01224136
axo21668-f	LOC1001761	1.224E-06	1.09E-06	-1	0.20893035	0.53077271	0	1.10099888
axo21717-f	acbd5	0.000445	0.00203024	-1	0.63139249	0.41678574	0	0.9104971
axo21901-f	NCKAP5	0.000209	0.00050793	-1	0.44345256	0.64795684	0	1.07839721
axo21940-r	SPF1	0.004964	0.00577849	1	2.37656303	0.69150773	0	1.10606001
axo22108-r	SPF1	1.64E-07	5.16E-07	1	9.40946698	0.0741289	0	1.47734421
axo22142-f		0.005634	0.00223094	-1	0.62247307	0.95002431	0	1.00746659
axo22246-f	ORF2p	0.000089	0.00050816	-1	0.22373135	0.47475417	0	1.24524044
axo22287-f		0.00348	0.00339518	-1	0.27417235	0.66935824	0	1.16010817
axo22665-f	ppan	3.46E-07	9.30E-08	1	3.69957958	0.73748194	0	1.03323374
axo22747-r		0.004797	0.0070909	-1	0.53654336	0.15782319	0	0.75498052
axo22792-f	LOC1001454	0.002414	0.0002885	-1	0.64014886	0.64483166	0	1.03951266
axo22829-r		0.003178	0.00067965	1	1.83528401	0.68552243	0	1.05333962
axo22851-f	CCNI2	0.00007269	4.33E-05	1	2.29859347	0.57093779	0	0.93213576
axo23066-r	TNIP1	9.584E-06	0.00020127	1	1.84285599	0.44703257	0	1.08807902
axo23068-f		5.53E-07	1.02E-06	-1	0.0621019	0.76827069	0	0.92404029
axo23213-f	pat	0.000863	0.00983074	1	1.34529634	0.2752939	0	1.11334264
axo23311-r	LOC1002288	0.000011	1.75E-06	1	3.39879104	0.14177612	0	0.82309926

axo23328-f	PRIC285	0.001557	0.00044585	1	1.98021306	0.60270799	0	1.07373055
axo23396-f	zgc:194655	0.000338	0.0005792	1	2.76205491	0.91628609	0	1.02223035
axo23479-r	tacc3	0.003673	0.06253681	0	0.83657763	0.00437654	1	1.36694545
axo23510-r	dlc	0.002468	0.00082517	1	4.45722256	0.44586735	0	1.28427842
axo23514-f	Pdcd1lg2	2.11E-09	2.93E-09	1	11.7388377	0.46046781	0	1.10000621
axo23580-f	LOC733758	0.004786	0.0031396	-1	0.56540243	0.20838067	0	1.2186003
axo23595-f	BIK	1.159E-06	2.37E-06	1	11.2495834	0.08345441	0	1.61661372
axo23634-f		0.00001425	2.09E-05	-1	0.26043273	0.99812661	0	1.00042799
axo23724-r		0.001459	0.00580516	1	3.58436445	0.50582452	0	1.28577363
axo23726-f	UTS2R	1.03E-07	8.49E-08	1	15.8136629	0.27617956	0	1.25667274
axo23747-r		0.00002242	8.17E-06	1	3.00403787	0.98836842	0	0.99805369
axo23749-f	pycard	0.000813	0.00036207	1	2.95246304	0.54373355	0	0.87966879
axo23760-f	wbp1	0.00092	0.00012959	-1	0.65326941	0.67241386	0	0.96988123
axo23804-r		0.000025	2.67E-05	1	2.84938849	0.72693706	0	1.05248646
axo23871-f	LOC1002258	0.000393	0.00029458	1	1.75595962	0.57731752	0	0.94227778
axo23876-f	c10orf26	1.375E-06	2.98E-07	1	3.42044097	0.6753343	0	0.95735064
axo23973-f	LOC1002328	0.001768	0.00131605	-1	0.42248939	0.29206915	0	1.24141229
axo23975-f	BEST1	0.001435	0.00274731	1	1.96126435	0.4091703	0	1.15765925
axo23984-f		0.00007028	0.0000373	1	7.23337526	0.50828091	0	0.82518243
axo24017-f		0.003428	0.00864598	-1	0.23645824	0.53699921	0	1.32612307
axo24032-f	IFIH1	0.000001	1.05E-06	1	3.48856247	0.07050179	0	0.78774467
axo24074-r	BEST1	0.00004	0.00028728	1	2.39094786	0.45198292	0	1.13276872
axo24100-f	LRRC32	0.000379	0.00016859	1	9.9086007	0.50493395	0	0.76301254
axo24105-f	igl	0.001506	0.00045474	-1	0.47327786	0.09856743	0	1.30340672
axo24112-r	Lynx1	3.32E-07	2.10E-07	1	5.05878176	0.48504284	0	1.09745871
axo24155-f	CACHD1	0.00001066	3.97E-06	-1	0.20020709	0.7275618	0	0.93900495
axo24159-f		0.00001424	9.78E-06	1	5.72610583	0.67072456	0	1.09686451
axo24192-f	zgc:113208	0.001702	0.00121728	1	1.47851102	0.71879925	0	1.03282846
axo24223-f	LOC557984	9.596E-06	2.26E-05	-1	0.02363569	0.92165805	0	0.95088031
axo24287-f	ASF1A	0.00008121	0.00020344	1	2.65986004	0.67748428	0	0.92942048

axo24309-f	ctnnal1	1.22E-07	1.22E-07	1	6.72677654	0.93569794	0	0.98830164
axo24343-f	LOC1001455	0.003921	0.00156462	-1	0.42152964	0.43816325	0	1.17522229
axo24375-f	Myo3	0.005692	0.00330524	-1	0.53187741	0.95627739	0	1.00926148
axo24377-f	LOC1000525	0.000779	0.00061333	1	2.37700236	0.97787789	0	1.00499338
axo24390-f	LOC1001706	0.000334	0.00028343	1	3.69607748	0.95172171	0	1.01491038
axo24406-f		0.000758	0.00043719	-1	0.58770741	0.40714611	0	0.91494695
axo24413-f	LOC443664	0.000266	4.9293E-05	-1	0.4654205	0.7542391	0	0.96462114
axo24432-f		6.63E-07	6.82E-07	1	5.29984806	0.05799909	0	1.37886703
axo24458-r	NCAM	2.243E-06	1.50E-06	-1	0.29651945	0.32652272	0	1.13078214
axo24568-f		0.001347	0.00304394	1	1.69163424	0.82809521	0	0.97040687
axo24579-f		0.005897	0.00171705	1	1.76760674	0.91129092	0	1.01538886
axo24588-f		0.0005	0.00055405	-1	0.5957982	0.33073339	0	0.89971418
axo24634-f		0.000318	0.00014468	1	2.1146306	0.45418695	0	1.10245008
axo24657-f		0.00008588	2.65E-05	1	2.51278633	0.97827747	0	1.00350166
axo24666-f		0.005333	0.00127007	-1	0.21132426	0.53303851	0	1.25259903
axo24716-f		0.00000731	0.0000034	1	4.73275093	0.96539801	0	1.00744681
axo24728-f		0.00078	0.00652762	-1	0.51594362	0.32888524	0	0.82050441
axo24777-f		0.003108	0.00068193	1	2.16456731	0.69667536	0	1.06569727
axo24805-f		0.00008	4.50E-05	1	2.08781662	0.92009776	0	0.98909889
axo24839-f		0.002076	0.00203672	-1	0.46065524	0.77761546	0	0.94728427
axo24847-f		0.000689	0.00080046	-1	0.37752829	0.81353924	0	0.95166651
axo24867-f		0.000602	9.06E-05	1	4.42424517	0.85193373	0	0.95608028
axo24868-f		0.003582	0.00686298	1	1.39286941	0.89619887	0	1.01310942
axo24886-f		0.001738	0.00038504	1	1.75160038	0.4757846	0	0.92413466
axo24915-f		2.30E-08	6.71E-09	-1	0.13652626	0.71232185	0	0.95932618
axo24936-f		0.004953	0.00411229	-1	0.21765942	0.98094173	0	1.01010271
axo25107-f		0.0032	0.00149651	-1	0.7452319	0.17366136	0	0.9056457
axo25120-f		8.33E-07	2.19E-06	-1	0.30858019	0.82622438	0	1.02744797
axo25121-f		0.0000968	3.14E-05	1	2.08192486	0.00330448	-1	0.67612599
axo25129-f		0.002183	0.00732369	-1	0.52025158	0.83403745	0	0.95910478

axo25133-f	0.002763	0.00205337	1	2.20051875	0.71284611	0	0.93052662
axo25173-f	0.000303	0.00028219	-1	0.31023972	0.40059383	0	0.82916397
axo25179-f	8.61E-08	6.25E-07	-1	0.21638669	0.32119988	0	0.8674034
axo25215-f	0.00008057	0.00085568	1	1.93196506	0.70980394	0	0.9479468
axo25225-f	0.000953	0.0036199	-1	0.33712573	0.3975832	0	0.77617973
axo25306-f	0.000754	0.00115816	-1	0.40940511	0.31924103	0	0.81275795
axo25310-f	0.00105	0.00013845	1	1.93065226	0.47727775	0	0.92239163
axo25337-f	1.04E-07	7.19E-08	1	4.20753907	0.79576155	0	0.97341259
axo25359-f	6.02E-07	4.40E-07	1	2.73218593	0.14378796	0	0.87222767
axo25360-f	0.000163	0.00011761	1	3.66956232	0.90628818	0	0.97475372
axo25386-f	0.00001626	8.14E-06	1	3.572065	0.73926587	0	0.94969796
axo25430-f	0.00035	0.00086432	1	1.52273406	0.7514696	0	0.97135507
axo25482-f	0.000316	0.0001179	-1	0.58637751	0.58914141	0	0.95262105
axo25538-f	0.000212	0.00039338	-1	0.24035205	0.25564852	0	0.7206232
axo25558-f	0.001487	0.00186204	1	3.75535944	0.6989423	0	0.88239509
axo25564-f	0.001067	0.00256056	-1	0.23466339	0.87763642	0	1.05877987
axo25571-f	0.00002315	0.00002014	1	3.6321304	0.90004879	0	1.02210022
axo25573-f	0.000262	8.34E-05	-1	0.51130569	0.51291676	0	0.93142532
axo25597-f	0.00009661	0.00011564	-1	0.48250585	0.5557265	0	0.93032089
axo25608-f	0.00001022	1.54E-05	-1	0.336595	0.1730145	0	0.81557512
axo25660-f	0.00301	0.010958	-1	0.48231547	0.52460598	0	0.85742378
axo25672-f	0.002026	0.00170539	-1	0.28713272	0.6952065	0	0.8887244
axo25673-f	0.005614	0.00117722	-1	0.68685208	0.53509943	0	1.05501292
axo25681-f	0.003606	0.00070698	1	2.05833718	0.35782003	0	0.86618174
axo25693-f	0.00065	0.00026696	1	3.11042992	0.37627724	0	1.209477
axo25716-f	0.000027	0.00001466	1	3.93933834	0.19113343	0	1.2762002
axo25741-f	0.00005935	5.58E-05	-1	0.47329514	0.4475965	0	0.91588096
axo25752-f	0.000715	0.00098593	-1	0.32222634	0.46075516	0	0.82864955
axo25775-f	0.00006548	2.20E-05	1	4.86284071	0.92533323	0	1.02041664
axo25840-f	0.000303	0.00017813	1	2.84668133	0.26825361	0	1.23453522

axo25922-f	0.000445	0.00053796	-1	0.46804355	0.51051667	0	0.90215652
axo25950-f	0.000171	3.87E-05	1	4.17751111	0.46023941	0	0.85544895
axo25958-f	0.000045	0.00004855	-1	0.41384402	0.95459287	0	0.99250343
axo25979-f	0.00445	0.00767043	-1	0.51174287	0.04469565	0	0.63043928
axo26014-f	0.005531	0.00074029	1	1.57841474	0.49520282	0	1.06942354
axo26092-f	0.004444	0.00233117	1	1.74056939	0.8607898	0	0.97578148
axo26099-f	0.00073	0.00010756	1	2.40847564	0.36442854	0	0.87407703
axo26126-f	3.543E-06	1.06E-06	1	12.1392552	0.70349267	0	0.91193022
axo26161-f	0.002986	0.00216025	1	1.68683516	0.85631847	0	1.02388357
axo26184-f	0.000248	0.00026576	-1	0.16196103	0.51883002	0	1.24628585
axo26199-f	0.000705	0.00086932	-1	0.3493286	0.93536112	0	1.01875749
axo26232-f	0.00142	0.00030573	1	4.38292502	0.70413768	0	0.89908328
axo26386-f	0.00001517	4.36E-05	1	2.58909398	0.84488182	0	1.02793117
axo26418-f	0.001282	0.00021864	-1	0.5055174	0.41358169	0	1.10807715
axo26600-f	0.000318	0.00032666	1	3.02063877	0.96110988	0	0.98978266
axo26678-f	0.000665	0.00038482	1	2.08017466	0.71499639	0	1.05365264
axo26838-f	0.000174	0.00012099	1	4.42681781	0.65436951	0	1.11877015
axo26850-f	0.002559	0.00073736	-1	0.49390666	0.37892568	0	1.14437588
axo26867-f	0.000271	9.2259E-05	1	1.89889833	0.53372056	0	1.06751475
axo26877-f	0.00003274	1.98E-05	1	25.1088608	0.52383255	0	1.32263288
axo26905-f	3.90E-07	1.93E-06	-1	0.24013063	0.49946365	0	0.90418591
axo26908-f	0.003002	0.00092636	1	2.03945405	0.97163863	0	0.99444581
axo26931-f	0.00005459	7.11E-05	1	2.51111018	0.09937456	0	0.77394985
axo27002-f	0.000607	0.00097219	-1	0.35997085	0.82830716	0	0.95208195
axo27008-f	0.004531	0.00288595	-1	0.33304015	0.83308823	0	0.94133382
axo27021-f	0.00521	0.00072038	1	1.7142276	0.522122	0	0.92868377
axo27094-f	0.001194	0.00025007	-1	0.20060985	0.88592295	0	1.04332502
axo27168-f	0.002461	0.0018517	-1	0.41348799	0.57269776	0	0.8849366
axo27174-f	0.000379	0.00074363	1	1.38830062	0.8182979	0	0.98404532
axo27182-f	0.000328	0.00102921	-1	0.43749729	0.08627737	0	0.70980075

axo27198-f	0.001265	0.00018238	1	1.68718636	0.82380597	0	0.97961946
axo27227-f	0.004472	0.00384923	1	2.2068634	0.63087787	0	0.90080745
axo27228-f	0.000579	0.00014396	1	2.46550583	0.86960735	0	1.02572311
axo27248-f	0.001218	0.00025298	-1	0.2691214	0.16283291	0	1.42708869
axo27263-f	0.005411	0.00105562	1	2.01886811	0.32750599	0	0.85380193
axo27294-f	9.867E-06	2.37E-06	1	6.06467079	0.43970513	0	0.86113719
axo27306-f	0.00003923	0.00014452	-1	0.57228538	0.29364798	0	0.90169827
axo27325-f	0.00005016	0.00008291	1	2.71803431	0.69177435	0	1.06572016
axo27403-f	0.005601	0.00112492	1	3.03850851	0.32587288	0	0.77628375
axo27412-f	0.000042	0.00005441	-1	0.46673978	0.22171895	0	0.86313732
axo27453-f	0.000842	0.00064425	-1	0.42088782	0.33003276	0	0.83478138
axo27454-f	0.000262	8.49E-05	-1	0.33500255	0.55257879	0	1.11086436
axo27455-f	0.000044	1.82E-05	1	4.03903879	0.82012888	0	0.95852923
axo27458-f	0.000455	0.00018959	1	2.15471491	0.41209064	0	0.892475
axo27516-f	0.001099	0.00028281	1	2.45018271	0.72757711	0	0.94313945
axo27521-f	0.00004768	3.50E-05	1	1.83154947	0.27513661	0	0.90647156
axo27542-f	0.004125	0.00461695	-1	0.46218457	0.57861782	0	0.88551992
axo27549-f	0.00002866	0.00001347	-1	0.33964833	0.75868776	0	0.95810322
axo27556-f	0.00000442	2.89E-05	1	4.0286794	0.59572864	0	1.11052638
axo27609-f	0.000262	5.16E-05	-1	0.40984416	0.93676003	0	1.01073631
axo27628-f	0.000906	0.00034466	-1	0.27778681	0.14222375	0	0.68230688
axo27635-f	0.000889	0.00072779	-1	0.3592548	0.46085528	0	1.17671151
axo27640-f	0.003222	0.0009374	-1	0.36488801	0.39063759	0	1.21451025
axo27645-f	0.000261	0.00017033	1	3.80211983	0.53687236	0	0.86429956
axo27663-f	0.00007871	4.2727E-05	-1	0.42274785	0.45228372	0	1.10173633
axo27672-f	0.000205	4.67E-05	-1	0.20396479	0.73571508	0	0.92289281
axo27678-f	0.00001053	7.21E-06	1	3.65394605	0.95516696	0	1.00876491
axo27709-f	0.000259	0.00006271	1	1.75500929	0.96375989	0	0.99605893
axo27711-f	0.001777	0.00047919	1	5.54003081	0.80466134	0	0.91845992
axo27742-f	0.00001625	7.32E-06	1	3.44282218	0.91905823	0	0.98503598

axo27747-f	0.002762	0.00183573	1	2.38801737	0.32256969	0	0.80671948
axo27758-f	0.000377	0.00027017	-1	0.39834637	0.47748746	0	0.8841514
axo27762-f	0.002177	0.00605482	-1	0.46827784	0.75222016	0	1.07331683
axo27849-f	0.005017	0.00278327	-1	0.52498593	0.55617526	0	1.10426442
axo27850-f	0.001867	0.00055348	1	2.61395203	0.74818819	0	0.93869237
axo27880-f	0.003696	0.0041811	1	2.05364954	0.959286	0	1.01021801
axo27885-f	0.000725	0.00073809	-1	0.55266545	0.34416525	0	0.88488446
axo27887-f	0.00007256	1.45E-05	-1	0.40937805	0.29333973	0	1.13372101
axo27892-f	1.99E-08	8.28E-09	1	25.735359	0.35542439	0	1.19348078
axo27896-f	0.000213	6.18E-05	1	1.97625576	0.93966243	0	0.99207746
axo27912-f	0.002938	0.00084076	-1	0.24552758	0.65950261	0	0.87385491
axo27930-f	0.002473	0.00101421	-1	0.48253316	0.44373393	0	1.1345488
axo27969-f	6.228E-06	1.13E-06	1	3.35608673	0.71946715	0	0.95839902
axo28004-f	0.00009189	0.00018285	-1	0.44917203	0.75896379	0	0.95744598
axo28009-f	0.00037	0.0003129	-1	0.62636521	0.06534963	0	0.83596976
axo28031-f	0.001539	0.0021588	-1	0.51414149	0.07243838	0	0.7225273
axo28043-f	0.003401	0.00153004	1	2.82360706	0.94705797	0	1.01643315
axo28076-f	0.003443	0.00153349	1	4.3951833	0.68862634	0	0.86855353
axo28085-f	4.78E-09	2.40E-09	-1	0.14226546	0.02994137	0	1.27645646
axo28107-f	0.005205	0.00646844	-1	0.50147372	0.47511002	0	0.8616455
axo28112-f	0.000998	0.00039161	-1	0.29711095	0.1648353	0	1.41420637
axo28176-f	0.001563	0.00387263	-1	0.62076638	0.53776763	0	1.08447415
axo28206-f	0.000196	6.21E-05	-1	0.30539203	0.37175862	0	0.84619574
axo28214-f	0.000143	0.00113126	-1	0.35303146	0.18402786	0	0.72029028
axo28249-f	1.908E-06	3.53E-06	-1	0.23491075	0.67724455	0	0.93528006
axo28253-f	0.003127	0.006865	-1	0.45598407	0.3155458	0	0.7836183
axo28268-f	0.00441	0.0024071	-1	0.54612231	0.55165336	0	0.91203599
axo28274-f	0.001308	0.12808503	0	0.87727922	0.00185331	1	1.39392313
axo28277-f	0.00008175	0.00119326	-1	0.67523113	0.01592739	0	0.77596133
axo28320-f	0.000271	0.0001084	-1	0.31851525	0.88917516	0	0.97397546

axo28341-f	0.000989	0.00345223	1	1.73614005	0.18492712	0	0.81412544
axo28362-f	0.00238	0.00074954	-1	0.3899271	0.68926944	0	1.08401797
axo28423-f	0.00002638	0.00004908	-1	0.08432485	0.32131013	0	1.45883596
axo28425-f	0.005197	0.00180507	1	1.85796673	0.40701405	0	0.88090804
axo28447-f	0.001621	0.0037712	-1	0.22184101	0.99491511	0	0.99739255
axo28501-f	0.005916	0.01210556	1	1.61082116	0.47147497	0	1.12340438
axo28512-f	0.002584	0.01062527	-1	0.48106909	0.51773038	0	0.85548927
axo28561-f	0.000388	0.00043911	-1	0.19687389	0.91659256	0	1.03431981
axo28620-f	0.0000563	0.0021023	-1	0.45435452	0.16004882	0	0.74829844
axo28680-f	0.00002426	2.00E-05	-1	0.12715429	0.87394292	0	1.04511618
axo28711-f	2.215E-06	1.66E-06	1	10.8442347	0.20144925	0	1.381367
axo28712-f	0.002119	0.00407093	-1	0.58099111	0.39316827	0	1.13911416
axo28718-f	0.001121	0.0024016	-1	0.65557527	0.2033921	0	0.8675908
axo28720-f	0.004402	0.01063953	-1	0.48555827	0.38605441	0	0.81188164
axo28724-f	0.003788	0.0009106	1	1.96425741	0.72702793	0	1.05321015
axo28726-f	0.000107	0.00032007	-1	0.55627647	0.22817532	0	0.8696885
axo28816-f	7.262E-06	1.87E-06	1	2.0221995	0.86510215	0	0.98775467
axo28821-f	0.001018	0.00037323	-1	0.47190445	0.53545569	0	0.91285904
axo28822-f	0.00194	0.00695392	-1	0.55115411	0.85828682	0	0.96837696
axo28839-f	0.00082	0.00056386	1	2.55892051	0.74015008	0	0.93792144
axo28854-f	0.00036	0.00024666	-1	0.14114807	0.49860066	0	1.27921433
axo28883-f	0.000253	0.00037516	-1	0.33555016	0.92040534	0	1.02140569
axo28933-f	0.001438	0.0002996	1	2.73296566	0.68168704	0	0.92494918
axo28985-f	0.000649	0.00020101	-1	0.32581133	0.4016206	0	1.18691559
axo29014-f	0.001123	0.00053008	1	3.21090875	0.52354737	0	0.8581672
axo29016-f	0.000309	0.00102104	-1	0.55847399	0.5067295	0	0.91647134
axo29042-f	0.00057	0.00401461	-1	0.66095426	0.33856813	0	0.89438568
axo29091-f	0.00075	0.0002024	-1	0.40391072	0.99373439	0	0.99872681
axo29150-f	0.003162	0.00522269	-1	0.3798685	0.90695477	0	0.96801658
axo29167-f	0.00000426	1.49E-06	1	6.10347413	0.72879946	0	1.06518696

axo29193-f	0.00399	0.00624389	-1	0.26734248	0.46027071	0	1.34009134
axo29212-f	0.001614	0.00130881	1	1.81674592	0.58382953	0	0.92665229
axo29216-f	0.001406	0.00094896	1	1.6252057	0.58266496	0	0.9423934
axo29241-f	2.89E-07	1.13E-07	1	5.76607526	0.67723376	0	0.9459265
axo29271-f	0.00112	0.00024614	-1	0.50949457	0.98409025	0	1.00247135
axo29288-f	0.000242	6.41E-05	-1	0.36437772	0.44310433	0	0.88527853
axo29299-f	0.002597	0.00091864	1	3.37144252	0.26226082	0	0.73391082
axo29358-f	0.00301	0.00417449	1	1.94409673	0.86577013	0	1.03159975
axo29385-f	0.00002757	0.0000547	1	6.73313473	0.55821088	0	1.18675599
axo29401-f	0.000218	0.00073472	1	5.7650695	0.99620629	0	1.00177349
axo29431-f	0.000447	0.00062422	-1	0.50088292	0.34239229	0	0.86949803
axo29490-f	0.002533	0.01108699	1	1.52106275	0.37013317	0	0.88139808
axo29526-f	0.000525	0.00044571	1	1.66987873	0.34522716	0	1.10344139
axo29527-f	0.001087	0.00053976	-1	0.47590604	0.05907553	0	0.72985241
axo29537-f	0.003119	0.00434461	-1	0.34329006	0.71385106	0	1.11579784
axo29561-f	0.00406	0.00520163	-1	0.56584847	0.46413341	0	0.88570756
axo29567-f	0.000164	0.00006116	1	4.14185869	0.72100099	0	0.92455646
axo29614-f	0.00048	0.00029957	-1	0.54757178	0.39906218	0	0.90711246
axo29615-f	0.000664	0.0026298	1	1.79504996	0.03259308	0	1.43925045
axo29664-f	0.000165	0.00036647	1	2.90262393	0.82516842	0	1.04665163
axo29672-f	0.000237	0.00013191	-1	0.28704185	0.29979426	0	0.79797032
axo29676-f	0.0002	0.00013821	1	1.95519522	0.54511456	0	0.9325541
axo29706-f	0.001455	0.00019438	1	2.07321777	0.95931529	0	1.00664384
axo29778-f	0.005855	0.0072779	1	2.26189402	0.61049784	0	1.13584502
axo29826-f	0.00008021	0.00005301	1	2.69702021	0.46602631	0	0.894901
axo29829-f	0.001688	0.00877992	1	1.98595739	0.81948267	0	1.05058872
axo29842-f	0.000005	1.12E-05	-1	0.19783222	0.70036077	0	1.08204463
axo29843-f	0.000417	0.00044726	-1	0.40743524	0.51382703	0	0.88886835
axo29862-f	0.000331	0.00025467	1	8.23333351	0.50921216	0	0.77139587
axo29867-f	0.001263	0.00078176	-1	0.37240818	0.37539869	0	1.2115103

axo29994-f	0.0018	0.00024892	-1	0.57611058	0.23922221	0	1.13181487
axo30009-f	0.003494	0.00149252	-1	0.41175717	0.34734804	0	1.22278351
axo30038-f	0.003078	0.00128784	1	2.15091324	0.2932194	0	0.82601888
axo30095-f	0.000658	0.00025439	1	2.49325999	0.30398841	0	0.83687458
axo30200-f	0.000252	0.00009573	1	5.4508794	0.54021214	0	0.84302364
axo30211-f	3.591E-06	4.89E-05	1	1.81663972	0.40654971	0	0.92714486
axo30381-f	2.05E-07	1.96E-07	1	11.1767177	0.75662407	0	1.06224754
axo30415-f	0.000896	0.00081157	1	6.8645826	0.23897864	0	1.66177685
axo30432-f	0.000136	5.86E-05	1	1.76216213	0.93001105	0	0.99240277
axo30439-f	0.001123	0.00021644	1	2.75620908	0.35133068	0	0.83981357
axo30442-f	0.000831	0.14581618	0	0.89920939	0.00422285	1	1.2826255
axo30482-f	0.004243	0.00072298	1	1.53459504	0.51827888	0	1.0611753
axo30572-f	0.000142	9.75E-05	1	2.43049304	0.73370451	0	0.95176172
axo30607-f	0.004064	0.00341167	1	2.60392038	0.62794563	0	1.13306111
axo30754-f	0.000276	5.68E-05	1	6.43022551	0.02975634	0	0.49497503
axo30795-f	0.00001875	7.71E-06	1	2.41572517	0.56738456	0	1.06333585
axo30867-f	0.002294	0.00308498	-1	0.65951041	0.8287054	0	0.97652891
axo30916-f	0.004711	0.00541669	1	6.06104267	0.28600432	0	1.77325047
axo31192-f	0.005007	0.00092168	-1	0.58634621	0.59666716	0	1.06451184
axo31214-f	0.005951	0.0059666	1	1.68766747	0.69031329	0	0.94032171
axo31223-f	0.004125	0.00481968	1	3.66135501	0.61334315	0	1.20573261
axo31234-f	0.00019	0.00010116	-1	0.57015233	0.34104297	0	1.09398225
axo31238-f	0.000014	6.88E-06	1	2.71718853	0.43775935	0	1.0984468
axo31256-f	0.001161	0.0001615	-1	0.3329319	0.82541275	0	0.95864772
axo31284-f	0.005017	0.00078867	1	2.01748041	0.30006238	0	0.85144131
axo31296-f	0.00000233	1.07E-06	1	10.8786855	0.56453232	0	0.8743988
axo31327-f	0.003401	0.00313696	-1	0.55380501	0.77220775	0	0.95573066
axo31329-f	0.000056	0.75020946	0	0.97184827	8.8716E-05	1	1.74155174
axo31347-f	0.00148	0.00633656	1	1.90203483	0.69575087	0	0.92777808
axo31376-f	0.000385	0.00050952	1	2.78269412	0.50992165	0	0.87111998

axo31398-f	0.005693	0.00291781	1	2.14780547	0.33960203	0	0.82260381
axo31403-f	0.00002274	3.70E-05	1	2.8042358	0.76309027	0	1.04620096
axo31404-f	0.002248	0.0009536	-1	0.41356366	0.55179801	0	0.88952805
axo31410-f	0.00008524	6.11E-05	-1	0.36838656	0.57080882	0	1.09190651
axo31416-f	0.001157	0.00066123	1	3.58391064	0.12345851	0	0.64435665
axo31429-f	0.00017	0.00008493	1	2.76968646	0.04017398	0	0.68637282
axo31432-f	0.00015	0.00011226	1	2.59099758	0.35198726	0	0.86021369
axo31461-f	0.00128	0.00093416	1	5.73011198	0.60344848	0	1.22262333
axo31464-f	0.003647	0.0092123	-1	0.45589484	0.89197442	0	1.03447133
axo31492-f	0.000365	0.0021233	1	1.78260987	0.81165347	0	0.96630554
axo31521-f	0.002164	0.01197055	1	3.13915805	0.41397655	0	1.37347438
axo31527-f	0.000108	7.20E-05	1	1.99245619	0.84635291	0	1.02123484
axo31560-f	0.000114	9.76E-05	1	2.19849411	0.94829016	0	1.00837179
axo31593-f	0.00008949	3.74E-05	1	2.84380919	0.07574814	0	0.74564474
axo31597-f	0.000321	0.0000798	1	3.24142082	0.39853335	0	0.85125956
axo31608-f	0.000149	0.00047884	1	3.51152328	0.3902994	0	1.24694111
axo31623-f	0.005748	0.00494264	1	2.62655841	0.40281403	0	1.26392337
axo31626-f	0.000121	0.00020321	1	3.00649592	0.67514217	0	0.92036243
axo31635-f	3.855E-06	2.13E-06	-1	0.28566179	0.07165869	0	1.29365955
axo31644-f	0.00002111	6.40E-06	1	4.09587494	0.62682652	0	1.08492251
axo31647-f	0.005216	0.00115078	-1	0.72574586	0.86614902	0	0.98781332
axo31649-f	0.000721	0.00707503	1	1.39884877	0.07322147	0	1.21978285
axo31677-f	0.004323	0.00870507	-1	0.62953013	0.3589185	0	0.87244333
axo31690-f	0.003897	0.00192664	1	2.13287389	0.98965022	0	0.99759767
axo31691-f	0.003607	0.00304085	1	1.76014384	0.04843685	0	0.72164339
axo31697-f	0.004812	0.00245582	1	2.34693302	0.97126192	0	0.99222096
axo31698-f	0.00008713	0.44920003	0	0.89543503	0.00003898	1	2.67889927
axo31705-f	0.001809	0.00105397	1	1.81478294	0.9234679	0	1.01290486
axo31716-f	0.000389	0.00019864	1	6.16501042	0.35853842	0	0.73717889
axo31729-f	0.000289	0.51504285	0	0.85282206	0.00012509	1	4.20661174

axo31731-f	0.003859	0.00087506	-1	0.40798971	0.31733776	0	1.22232478
